# Supplementary material for: Phylogeographic separation and formation of sexually discrete lineages in a global population of Yersinia pseudotuberculosis
Source: Microb Genom. 2017 Sep 18;3(10):e000133. doi: 10.1099/mgen.0.000133 (PMC5695210; doi:10.1099/mgen.0.000133)
Supplement: Supplementary File 1 [file mgen-3-133-s001.pdf]

Figure S1

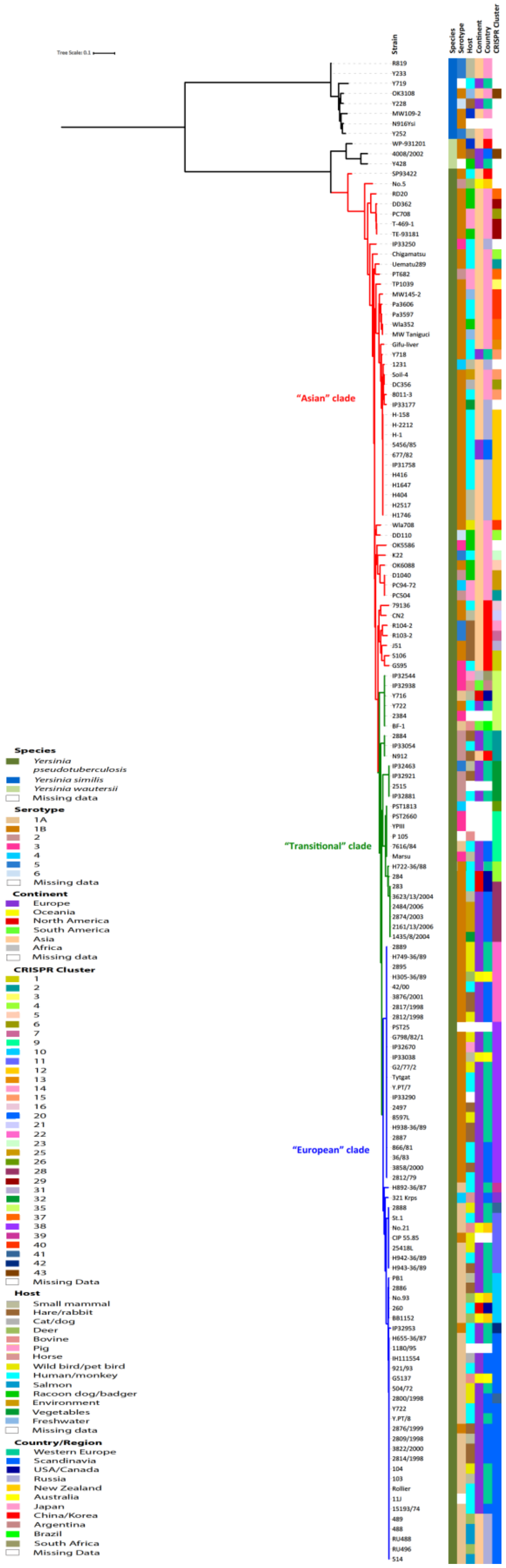
Tree Scale: 0.1
Strain
Species
Serotype
Host
Continent
Country
CRISPR Cluster
R819
Y233
Y719
OK3108
Y228
MW109-2
N916Ysi
Y252
WP-931201
4008/2002
Y428
SP93422
No.5
RD20
DD362
PC708
T-469-1
TE-93181
IP33250
Chigamatsu
Uematu289
PT682
TP1039
MW145-2
Pa3606
Pa3597
Wla352
MW Taniguci
Gifu-liver
Y718
1231
Soil-4
DC356
8011-3
IP33177
H-158
H-2212
H-1
5456/85
677/82
IP31758
H416
H1647
H404
H2517
H1746
Wla708
DD110
OK5586
K22
OK6088
D1040
PC94-72
PC504
79136
CN2
R104-2
R103-2
J51
S106
G595
IP32544
IP32938
Y716
Y722
2384
BF-1
2884
IP33054
N912
IP32463
IP32921
2515
IP32881
PST1813
PST2660
YPIII
P 105
7616/84
Marsu
H722-36/88
284
283
3623/13/2004
2484/2006
2874/2003
2161/13/2006
1435/8/2004
2889
H749-36/89
2895
H305-36/89
42/00
3876/2001
2817/1998
2812/1998
PST25
G798/82/1
IP32670
IP33038
G2/77/2
Tytgat
Y.PT/7
IP33290
2497
8597L
H938-36/89
2887
866/81
36/83
3858/2000
2812/79
H892-36/87
321 Krps
2888
St.1
No.21
CIP 55.85
25418L
H942-36/89
H943-36/89
PB1
2886
No.93
260
B81152
IP32953
H655-36/87
1180/95
IH111554
921/93
G5137
504/72
2800/1998
Y722
Y.PT/8
2876/1999
2809/1998
3822/2000
2814/1998
104
103
Rollier
11J
15193/74
489
488
RU488
RU496
514
"Asian" clade
"Transitional" clade
"European" clade
Species
Yersinia pseudotuberculosis
Yersinia similis
Yersinia wautersii
Missing data
Serotype
1A
1B
2
3
4
5
6
Missing data
Continent
Europe
Oceania
North America
South America
Asia
Africa
Missing data
CRISPR Cluster
1
2
3
4
5
6
7
9
10
11
12
13
14
15
16
20
21
22
23
25
26
28
29
31
32
35
37
38
39
40
41
42
43
Missing Data
Host
Small mammal
Hare/rabbit
Cat/dog
Deer
Bovine
Pig
Horse
Wild bird/pet bird
Human/monkey
Salmon
Racoon dog/badger
Environment
Vegetables
Freshwater
Missing data
Country/Region
Western Europe
Scandinavia
USA/Canada
Russia
New Zealand
Australia
Japan
China/Korea
Argentina
Brazil
South Africa
Missing Data

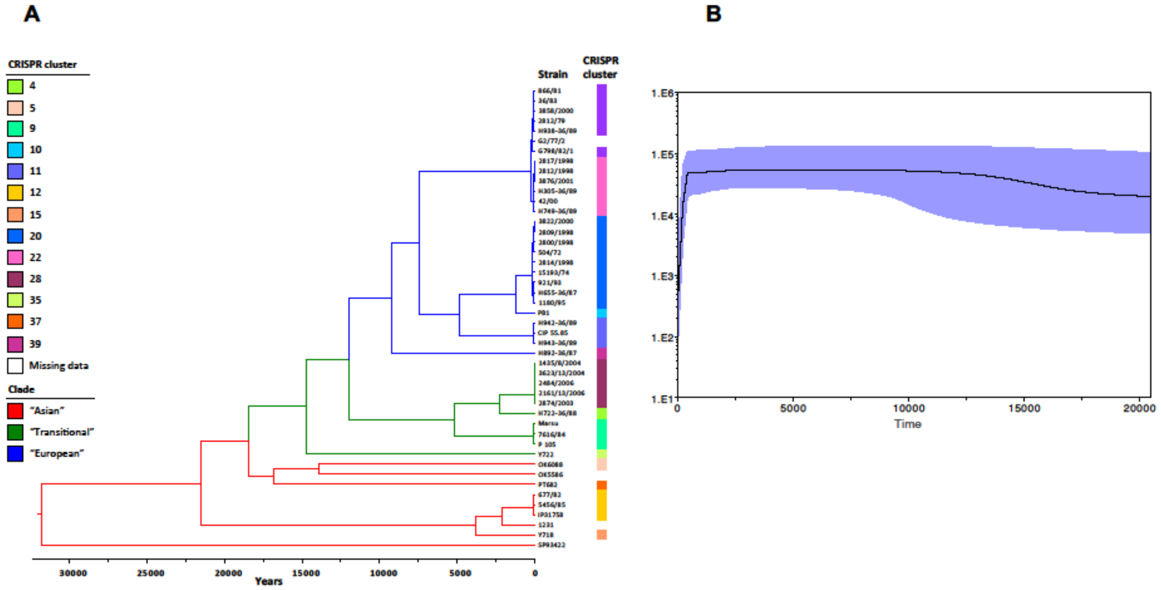
A
B
CRISPR cluster
4
5
9
10
11
12
15
20
22
28
35
37
39
Missing data
Clade
"Asian"
"Transitional"
"European"
Strain
CRISPR cluster
866/81
36/83
3858/2000
2812/79
H938-36/89
G2/77/2
G798/82/1
2817/1998
2812/1998
3876/2001
H305-36/89
42/00
H749-36/89
3822/2000
2809/1998
2800/1998
504/72
2814/1998
15193/74
921/93
H655-36/87
1180/95
PB1
H942-36/89
CIP 55.85
H943-36/89
H892-36/87
1435/8/2004
3623/13/2004
2484/2006
2161/13/2006
2874/2003
H722-36/88
Marsu
7616/84
P 105
Y722
OK6088
OK5586
PT682
677/82
5456/85
IP31758
1231
Y718
SP93422
30000
25000
20000
15000
10000
5000
0
Years
1.E6
1.E5
1.E4
1.E3
1.E2
1.E1
0
5000
10000
15000
20000
Time

Figure S2

Table S2. *Y. pseudotuberculosis* complex CRISPR spacers. The identification keys and the sequences of 2969 spacers are given.

Key	Spacer sequence
1	CTTTAAAACACAGGTTTTAGCGCCATCTGATAT
2	ATAATTGTTCTGCTTCAAGATTAGCAATTAAAA
3	AAGTTGACTCGTTTAATACACTCTGCAAGTTT
4	GCACTTACGCTCACTCGCAGTCGATGGCATCC
5	TAAAGACCGTTGAATTTAAACAAGGTATCAAC
6	TGAAAGGGATCGGGCTGAATGCGCAAGACGCG
7	AGTCAGGGCTATGAGGGGGCTGCAATGGGCGCG
8	GTTTTTTCGACCTTTTCCTCCATCAGCGAGGC
9	ACCGTTATTAATGGGTGGCGCTGGCGCTTCAT
10	ACGTGGCTAGTTATTACGATACAATTAATCTC
11	GCTGCTTTCTTGTCAATCCCTGACGGGTTCTT
12	ATCTCCGGAGAGTTCAACTGTGGGGTGGTGTA
13	AGATGCCGCATGAAGAATCACCCGATCAATCC
14	AATAAACATCAGCGGTCCCGCTGGATGAATAT
15	ATAACGAGCCATTATTTAGCACCTCCGAAATA
16	GTGGTATGTCTGGATAAGTTGGCTTTACCATA
17	TTGACAGTACCAGCAATAATCTGTATTCCAAG
18	TGTATTCTTACTAAAGATATCTACTCAGATAAT
19	TTAAGCATCACACAGCCGATGGTGTGGTTGAT
20	CCGGATAGCGAAACTTAATGAAAATATTATCGC
21	AACCACCGTTCATCACAATATCATCAACGATA
22	TTGATTGATGATGATAATTAAGTGGTGGCTAT
23	ACTCTTCTTCCCCTCCTCCCGCTGCAAGATGC
24	TTAATTCTCTTGCATACTTCATGCGCTGCAAGT
25	CCCGCCCGGCTTTGTCGGTCAGTTGACTTCAT
26	TGGCACATCAACACCAACCGTAAGGATATTCT
27	GAAGCGTTATGTGAATCCGTTGATATGGAACT
28	ATCGACTGGCTTACCAAGCGTCACGGTGAACA
29	CCAATAATTAATGCCTTTGTAATTGCTCTAAC
30	TTCCCCGCGAAGAACCCCGGAAGCTAACGCTT
31	CATGAAATACATGCCCGGTATGATGCCGGTAA
32	GATTGGCAAGGGGAGCGCTATCACTTATTGGC
33	GTCAACTTGTCAGTGCTTAGCCCCGACTCTCT
34	GTATTGAGCGATACTTTTATCATGCATATCCA
35	CGTTAAATCAATGACTTACGCAACACTCTATA
36	GATTATGGCCCTGTGCAATTTGTGGGCTACAT
37	GGCCTATTAACGGGTCGCGCCGTAGTGCTTGA
38	CCCCTCAAGTACCACCAGATTGTGTTCATAGT
39	CCCAGCGTCGGGTCGGATACTTGCCACGTAAC
40	TAGAGTGTTTTTTCTGACGATGTTCCTTGCTG
41	CGCATTTGCTCAACTTCTTTATTAGCATTAGC
42	GAATCAATATATCCAATTTTCCCGCCCCGTGT
43	TACTGCTCAGATATTTGGCGTGTCTTTTCTTG
44	ACCACCTCGCAATTCTTCCGCCAAATGTTCCC
45	AGAAATGAGTAACGTTCAATTAGCTGCGGAATT
46	AAGAGCCGCCTTCTTGTCTTCCGCGCGTTGTT
47	TGCATTACCTTCCGCGTTTAAAAGTGGGTTAG
48	GAATCGCAATCATGTTCTCACCAGCCAGATTA
49	GCGTCGCATTGCTGGGCGTCACATTAACACTC
50	ATTCCATGATGCTGTCAAACGTGGCCCCACTC
51	GATAAAGTGTTTATGGCTACTAGTTTAAGAGA

52	TTATACCGGCCCTGACCTGTTGATCATCGATGA
53	GTCGCCGGGTTTATGTTGCTCCGTTCTTACCC
54	TTGGCCTTTTTCAGCGTTGGAATATCATCAAT
55	AACCGGAACAGACAGGATTAAATAGCCATTGC
56	TTGCAGATTCATCCGCCGCGCCGTACATCATT
57	GCCGATAGCCTCGATGACGTCGCGTAATGTAG
58	CCGACTCACCGCCGCCCTGATCCATCCCCTA
59	ATGCGGGATACCCTGCAATGGCGTATTGCATA
60	GAATGCAAGTATATTGCAACCCATCCCAAACC
61	TAACAAACGCCGATTTCAGCATCCCAGTTGAA
62	TTTCCATCACGCCGCCTCATCGTGTTCAGCCA
63	GCTCGTCTGCATCTGTTATGGCTCATAGTTTT
64	AAGCCGTGCTTCTTGTCCCATCTCCATGACCT
65	TCAACGAACGTGATGTGCCGAAACATGATGTG
66	GTAAAGGGTTTGCGTCCGGTGTTGGTGCAGATG
67	AAAACGAGAAGTTACTCTGACGTTTTCGCGTT
68	TTCTGCAGGTTCTTCTGGTGCTCTTTCGACAT
69	GCCTCGCTGAACGTTGAGGGTGGTATGCACGA
70	TGTCTTCGTCCGGAGGGCTCCCCGGACGTGGC
71	ATCCCAATGTCTGGAAATGGATAAAATATTAT
72	CGCTTGTCACCGTCCACAATCACGAACACACA
73	AAGGGGAGGCGTGGCGTATAATGATTGCAGTTG
74	TATCTAGCACGGCCAGAGTACCTGCTATCTTA
75	AAGCGTGGGACGAATGGAGCAAAAGATTTGAT
76	CGTCCCCGCCCTGGCGTTGAGTGCCGCCTGGGC
77	ATAAATTGTTAGGTGAATATTGGGAGTCTATT
78	GCAGGCCAACGGGTGATGCCGGGCCATTCGTC
79	TGCTGATGGCCGGCCAGATAAGCCAATGATTA
80	ACACTCTTATTGCTAATATAGATAAAGATGGT
81	ATTTTTTGGATTTGGCATATCGCCTCTCGTTC
82	GTCAGGGCCAGCGCTTCGTTTAATCGTGCGCC
83	TACCTACCTCGCTGTTAATGATTCGGACTTAC
84	TATATTTAGCTTGGATAACATCGACTTCAGTT
85	CGTCTAAGATTTATGATGCGTCGGTTAGCGAT
86	TTATCAATTGGTTATGCCGTGCTGCGCATTCC
87	CGTTCACCATCTTCATCATCTGAGCGCCAGAA
88	CGTGTAAGATGATTGTTGCGCCCCTAGCAATT
89	GATATCCGATATGCCAAAGCAGGTATAATGTT
90	GACATATTCGCAAGGTTAGCGTTAGCCAGCGA
91	CTTTCTTGCCACCTAGCCTCACGCAGTACACC
92	GACCGACGGCTGCCAGGACTGCAAACCCTGCT
93	GAAGAAATAAACCGTATAGATGCTTATGCACA
94	GTGGGAGGGATTCAAACTACGCTACAGCTAAT
95	TGGAAAGAACACACGTTACTGATGATTGGAAT
96	AATCGGGGCGATGTCTAATAGTGACGTCAATT
97	GGATGCTTGCTGGTCAATCGTGAATCCTTTAT
98	TTCCTGAATTAGCATCACTTTTAAGACTAGGA
99	GTCATTACTGTTTTTGCAAATTCTTTTGCGATT
100	GTAGGAAATCACCCAGTGCATTTATTGCCGTA
101	CGTAGCCCATGTTGCCGGGTGGTTCTTAAGGC
102	GTAGAAGATGCAGCACGTTCAACGGTGGTGTT
103	GTCCCCTTTTTGCAGATCTGCAAATTAAAGT
104	TCTCGCGCGGCACCATTAGCAGCATGATTAGC
105	GATCAGGAAGGTGTCTATATTCTCGTTTACGT
106	CTACCGGATTGAGATTTAGATCACCTCCTGTA
107	TATCACTGAACTAGTCGAGTGGATAGCAGACT

108 TGCATGATCCGAAAGCTGCCATCAAGCAACTGG
109 TCAGGTGCGCCGGGAATGTTTTTGCCGAGGTA
110 TTTTAATATTATTAATTTCATCCCATTGCTGT
111 TACCTTTAAACCCTAATGGCGTTGATTATTCT
112 ATTTAAGTCGAGCGTGTGAAGTTTATGCTAAT
113 ATAAATGTATTCCTCCATTCTGTTAAGACTTG
114 ATCACTTATTAATTATAGTGTTATACTTATTT
115 GTCGTTATAACTTTGTGCTTGCGTCAATCCTT
116 CCATTAAAAGAAGATACGATTTAGAATCCATGT
117 ACCAAAATTCAAATTGAGTCGCGAATGTCTGC
118 AAAATATCAGCCATGAATTTATGACAATTGAT
119 TTCATTTCTTTGCGGCCAACCGGCTTGGCTCG
120 AAGCCGCCTATTACTCGGAAATTATCCGGGCC
121 AATATAAATACTAAAACCCCCTTAATCCCCCA
122 AAGCAGCGGCCACATTTTGTTGTTCTGTGGAA
123 GCACCATTAATCGTTGTGCGCCATTTTCAGCC
124 ATCGATATCAAGCTCGAGATAATCGCCGTAGT
125 GGGATGGAGGCTGGATTGTCTACTCTCGAAAT
126 AACTTACCATCCAACTTCCCATTAAACTTTGA
127 ATATTGAAGTCAAACGCTCAGGTGAACGAGGT
128 CATATGGCAAAGGTGCATTAACTACAGCATCA
129 ATCATTCATTAAGTCTTGGCCTTCGTCACTACG
130 GTGCAACAGCGTTTAATATCCCAGATTTTTTG
131 ACCGCCAGTAGCTCCAAATACTACCATTTCCG
132 CTCGACGCGTTGAAACAAACCTTTTGCCGTTT
133 TTTTATTTGGTGATATTTGAATTGATCGGCAA
134 ACGACGGTTAACAGCACCTTGCCCCAGTGGCA
135 GCCAGATACAAGATAATTGAGCGTGATGTCAA
136 GAGAACATACAAATAACATGTGAGTCCAGAGA
137 CCACACGCCACCCGTCCATTCGCCCGTTTCTG
138 TCTGCTGGTGATTTTATTGGTGCGCCTGATTG
139 ATGCGCAGCGCGTTAAGCATCAGTAAAGTGTC
140 TCTTTTAATGTCCCAGCAACTTCATTTGCTAT
141 TATAGAGATTATGCGAAATTAGAAGCCCAGCT
142 ACCGCTTTCATTGTCATCTGTTTAACCTCTCT
143 TCAAGTTGAATGAGCCGGTAATGATGAATGAC
144 AAGGTTAGCGTTAGCCAGCGCGGTACCAGATT
145 GCACTGAGCGGGGTGATGTCATGGTTCGCCGT
146 GATATTAAGAGATCTTTAAATTCATTGTTTGTG
147 GAAGGCCTGCGATTTGATGCATATCTTTGCCC
148 ATAATGGCTGCATTGCTTGGGTTTTATGTTGG
149 ATCTATAGCCGGTCAGAACCGCATTTTGCCGA
150 GCTACTAATTTCATGATTCGTTTAGGGAATGA
151 TGGCGCGCCGTCAATATACTTGGTTAGTTCTG
152 ATAGTAAGTGGAGGGTTTATTAAAATAAACAA
153 TTATCGCTAGAGTCAATTATAGTTTCGGTTTT
154 TCCGGTATCTCCGGAGAGTTCAACTGTGGGGC
155 ATGCTATGGACACAGTGGCGACCAACACAGAT
156 TTTCCTCATGAGCGCGTGAACTGGGTTGAGAC
157 GGTTAAGCTGTAAAAGCGTCTCGCCGCCGTAG
158 AGAGTTGCAGGGATGCAAGTCAGCGCGGGTTT
159 ACATCTGGAATCAAGGGGGTTTCTTGGCACAA
160 ACTACCAACTCTGATTTCACTATCCCTATCGA
161 TCCATTTTCTCTGCCCATTCCGCATTGTGATA
162 GTTTTAGAGGTGGCGGCGGGTTGCGCCGATAC
163 TTTACCGGCTGCTATTGCATCATAAATCACTT

164	TGATATCTTCAGCCTTTAATTGACGCTCAAGC
165	TGACCTTGCTTGCGCTCATCCTTGTCTTTCAG
166	GGAATGAGAAGAAATGCTCACATCTTCCATCT
167	TAAATGGCTGACATTCATTACCACAACCATTA
168	ACTGTTTTTGCAAATTCTTGAGCAATTGGCTC
169	GTAGATTGGCCGTGGGTCTGGCTGATTCGCTT
170	GTCCATCCAGACCGAGTACTTATTCTGGCTGA
171	ATGACGCCTGCTCGTTAGTCGGCGGCGGAGGT
172	AGCTTCGTAATGCCTTTAGACGTGAAACTTTT
173	AGATAGCAGGTAGATTGAATTCATCGGATTGC
174	ATGAGTGGTAAGCAAGCGGCTGAAATGGTTCT
175	ATTTCGGTGTTGACGCGTGCCGGTGTCATGGA
176	ACGCGGCACGGGCCAAATTCATGACACTGTCC
177	AATAGAAGGGGTTAACGAGGTTTTACGCTCCA
178	CTCGTATGCGTCGCGCATTTATCGATAATGAT
179	TTAAATGCTTCTTCTTCTTTAATAGCTGATTGT
180	ATACATAAAACCAGATAATGCGCCGCCAGATT
181	ACCGCCTTTACTCCGCTTTCTCTTATTGTTCT
182	ATTCAGAAAGCGAGTGATGGCGTAGATTCAGC
183	TCGAGGTGGAGCTGCCTGACTTTGATCCATAT
184	AAGCATTGCGCTACTAAAGGCTACCTCTCATA
185	ATACATTACTGCCGAGGTTCGTAATGGACATC
186	CAAGACATTGACCGAGATTAATTGTATCGTAAA
187	TCTCTGCTGACGGATTTGTCGGCGTCGATAGC
188	GTATATAAATTGATTTTCTTATTCCCCATATC
189	TTAGTGAGAAATGTTATTTCATCCCAATGTCA
190	CAGTTGAACCCGGCTCATTCCTGCTGGCTTAT
191	CTATTGGTGCGGGTTCGGCAACGACGGGGAAC
192	AAAGGATAATGAGGCAGAAATTAATTCTCCGT
193	ACTTCCGTCTTTCTTGACGGTGTAAACATTAA
194	CTGCCGATTCGCAGGTACCGACTTTTCACCCG
195	CTTGTTGATAATGCTAAGTGGCAGCGCGCAAT
196	ACTTGTTCTAACAATTGGTGGGATGTATGTAA
197	GGATGTTTTTGCGAAAACGCCGATTACCGGTA
198	GATATGACATTGATTCCAACTGGTGGCATGGGT
199	TTTAACACCCCGCTGGCCCTTCATCCGCACAA
200	TTGGTTTCCTATTTATTACAGATGTTCGTACA
201	AGCTAATACCATTCCATCCCAACCTATCTCTT
202	AACTAAGGACTCTCATGAGCATTAAAATTTCT
203	GAATACGGGGAACTTGATGACGTGTGTGTGAT
204	CCCAAAGCCAGGGCGGGTATTCAACACTGTGG
205	GAGATCAAGTAACTTATATGCTTATGTTGACT
206	TCTTCGGAGGTGCTGGCGCTTTTGGTGGAGAA
207	GAATGTGCATGTCAAAGATTTCAGATGATTAC
208	TAAGTTCACTAAACGAGAAGTGTCACTTAGAG
209	CGATCAGGGTAGATGAATGGATTGTCGAGGAC
210	GGATGATTGGCGTCCCGGAGGTCACAGAAGAA
211	CAGAACCCATCAGCATCGGCGTGCTGCTGATG
212	GGTTAAGGGATGCGCCGCCGCAACCCATCAAT
213	CTAAAATAGATGCTCTGGTTTCTTTTGATTCT
214	GTATGCCCTTTGAATGGGTTTATATTTTTAAC
215	TGAGTCTGGCGTCATTCTTGGTTATGGAAGTT
216	TCTTGAGCTACGGCGTACCCACTATCCGTAAT
217	ACAGACCTGTATTGGCTATTGAGTAACACGCT
218	TTTCTTCGGTCTGGCTGGCGGTGACTGTCGCC
219	ATTATCTGTATTATTAATGAGTGTTTATTGCT

220 GCGCTACCTTCTAGACGTTCCCGCGTTTTGAA
221 ATTACACTCACGCCGATATTGATTTTAGTCGT
222 TTTTCTGCTGGCAAAACTATCTGTTTCATCTG
223 CTCCTCATATTGCTCAATCTTTAATGTGCAACG
224 TATCTGGCGCTCTGAATGATCCTTGGCTGATG
225 GAAGCCCTCCGCGCTGCCGGCATTCGAATCAA
226 TAAGACAGCGTTCTGATGTTTTCGAGGGCAAT
227 ACGACTTTCAAAAGCACTATTCCGCCGCGAGC
228 GATTCGGTATCTTTTACCGTCTCCCAACACAG
229 TGGAGATAAACTAGACGGGTTGGATTGATAT
230 CTCCCCGATGATCTGGGGACTTAGTTTTAATG
231 TATTAATGCTGCTCGATCTGCCGGTTGGTTCT
232 TTAATGTCCAGCAAATAGACGGCTTGCCACTGG
233 TCTGCATTCTGCTCTATCAGTTCACCTTGTCT
234 GATGGGTCTCAAAATACGCACCAAAGGGAACG
235 GGATGAAGGTAATGTTATTGATATTAATACTT
236 GAACGCCACGCCATTGTTAATGGTCGGCTGAT
237 GTCTTGTGGAAAGAACACACGTTACTGATGAT
238 ACGAAAAACAAGATATAGAGATAGAATACGAC
239 ATTGCCGCCTCAGCAGCTTCCATTATTGCCAT
240 ATTCAATGAAAGAGTTGGGGTTTTCAACGGAA
241 TGTATGGCTCAGAATAAAGCACCGACGCTAAT
242 TACTGAGTTTAAATTATCCGATCAACGTGGCA
243 TGTCAGAGCATGTTTACGCGATTGCGCCAACG
244 TAACTTCACTTCCCCTTGTTCCCAGCGTTCCT
245 GAGTTGATCGGCATTGCGACCGTTGGAGCCTG
246 ATCTAGCTTCTCACCCTCTGACGGCTCAAGCA
247 TTCACACTGGCCCGCAAGGTGTCACTGATGAT
248 TAATAATGGCTTGGTTCGGTCAAACAAATTTA
249 TTTAACAGGCTCGCAAGGTCTTATAAAATTGT
250 TTCAAATTCTTTCCTATATTCAGCTGGCATAG
251 AAGATGCGCCTGTATTTGTGTATAAATAAACA
252 TGATTGGACGTGTGAAGGGAGTAATAGATGAT
253 TTACCTATATTAAAATCCTCTAACTTTAAAAC
254 AAGAATTTCGAGATTTAAATGTAGCCACACTA
255 AAAAACATTCTCTATATTCCTAAATTTGAATA
256 CCCCTCAGAATGTAGCGAATCAAACATAGATGG
257 TTCTGGATAGGACAAATAGGATGATTGTATCA
258 CCTAATCTGGCGTTGTTCAACTCAAGGGTGAT
259 ATACATTTACCGCAGATGTGATTGGTGTTGAT
260 TTGCTTATGAGAATGGATATGAATTAACTTTT
261 CTAGAAATTTAAGAACACATGTACCCTATCAG
262 AGAATATACGCATTTATTCAACCTTATTACTA
263 GGGAATAGTATCTATAAACTAAACTTCGCGTA
264 CTGTTCCCCATATTTATAAGCTGATTCATATAG
265 ATAGTGATGTTAAAACAATTACTTCAATGAAA
266 TTTCCGCTTTTTTCAATTCAGCTCTATTCCTA
267 AGAAGCTATTCGCGAGCTGGTAAATCGATTGG
268 TAAATTTGTGTAAGCCTGTAATACTTTTGATAG
269 GGTGAGACAGGGGTGGATCAGCCTACGTTCCT
270 TACCCTAAATGGTTTCGTGTTCCAGACCGGTC
271 CTACCGGATTGAGATCACCTCCTGTA
272 CCAAGTCTTTTCCCCATTCCATATCGTTCATT
273 ACCTTTCTTCAAATTCTTTCCTATATTCGGCT
274 CATTATATGCATTAAAAGCTGATACGGTGCAA
275 AGACCACCTGACGCATTCAAGTTAATCTGAAT

276 ATTTGCCCCGCGCCAATGCCCATTTTATTATC
277 CTTGCCCGTTACCAGTAACCATTATTTCAATT
278 TAATGGCGTTAGACAATTTCTTTGAGGCGTAT
279 CCGATGACCACCGTTGCAAGCAATGTCATCAC
280 TGAAGTAGATATGGACACGACGGGCTTAAGCA
281 ACGGTTCCCGGCGAAGAAGATTGGCTGGCACT
282 TAATGATGATTGTGCGGCACTCTTATCTGCTT
283 TGATGTGCAGCATGAAAGCTATATTGCCTCAT
284 TTCAGTTTTCATCTCTTACCCCTTAACTATGC
285 GCCCTGTATGATTTGAATGCTTTTGTGTCCAC
286 TATCCCATCCATGCGCTTCTTATGCCCATGGT
287 CAGGAAACCCGCCTCAGTGACGCCGTTGATGC
288 TCTGGCAGCTAAGCCGTGAAGCTCTACAACAA
289 TCGCCGCCCACCGCCTCTTTCATCTCCGGTGC
290 CATCGCCACTCAGTGAGTTAACAACTGACTCT
291 ATTACGCCATCAGGAGTGGTTATTGATTATGTT
292 CGGACGCTAGAAAAATGCTGGGAGACTTATCC
293 GCATAAATAGACCGCGCGCATATGAACTCGCT
294 GACCGGTCATATTGCGGGTATACAGTGCCAGA
295 GAAAATAAAGTAATAGAAGTCATTAGCGTGTCT
296 TGCATCCACCTCGATACAGGTTGTACAGTCGA
297 CTGAATGGCTTGCCTGCTGATACACCGAGGTA
298 GAATAATATGCAATTTCTTTACCTTTTTCACC
299 TGCTGCTGCATATGTTGCTGACATATTCGCAA
300 ATTTAACAAAAACAGAGGATTATATAAAGTTA
301 TATCTTTATCAACTAATAGTTGGCCTGCTGTT
302 GACTTTGCTAAACTATTCATTGTTAGTTCCTT
303 ACCCACGGTGAGTATTTCAGTGACGCAGAACA
304 CCTCGGTTTCGAATGCTAATTTATAAGTTGTT
305 GTGATGACAACAAAAACCTACTTACGGGTAGT
306 TTTAGCTTCAGTATTTCGCGCTATGCCGGTTT
307 AGAGCGGACTATGGCTACAGCGCGAGCATCAT
308 GGTTCAGGTTTGACAATAACAGGCATGAGAAG
309 GCCAAACTCATCGCTGCCGCGCCGGAGCTACT
310 GGGTTTTGAATACAAAACAAACGCTTTTACAT
311 ACCTCGGTTTTCTTACTGCGGTTCATCTTCAAT
312 CATATAAATCATCAAGAACCGCTTGCTGTTTC
313 TATCAGAAAGTATTTACGACGCAATCGCAGAC
314 ATCTATTAGCTTTTTGGGTTTAATGCCTAAAT
315 TTCTCCGATAACTTCCAGTCTAGCACTATCTT
316 CTGAATCTGATGAACGGACGTCATTTAATGGA
317 ATTGCCGCCACAGTGCTAGCTGGAATTCGTTT
318 AGTTTCACTGAGCAACTCATTTTGTTCATATA
319 ACCATCTGTTATTAAGTCGATTATACCGTGAT
320 AAAACTCACTGGATACAAATGCTATTTTGGGTG
321 ATTGCCGCCGGCATGATGAACAAGCTAGGTCA
322 CCGTTAATTCGGGATATCTTTTATGGGACAAT
323 CTATCGTAAACGAACTTAAACCCTTCCGGCGT
324 CAGTAGAGAAGTTTGGCGAGTCAACTGACCGC
325 ATTGGGAATATGAAAATGAAGGTTATGAACTT
326 GTGAGAATGGGAGGCCAACAAGCTCAAATATA
327 ACCGTCATAGATATAATCAACGTATTGCAAGC
328 TGACTCAAAAGCTGCCGTTCCTTCTTCTGCCA
329 AAATGGGCAGATAACAAGATGGGCATCGGCGC
330 CACCGGCGGGTTCTTGCTCTTCGCCATCACAT
331 ATAATTAATTGTTGTGCTTCATCCAACGTGTC

332 ACATCCATTAAATAATCAGGATTAGTTATTGTT
333 ACAATGCAAGAAATTAGCGTTGATGTTATTGA
334 TTTTTGGGGTCGTGTCTATCATCACCCTGATA
335 CATATGTGCGCGTTTCCGGGTCGTAATTATCC
336 GCGAGTTTCATTTGCCTGACGTTCGATGTTCA
337 TGATACTATATTCATTCAATAACGGGTAGATA
338 CCATATATGACTCGATAACTTCTTTAGTTGCT
339 AGGTGCAGGAAAAACATTCTCGACTTTAAAGT
340 TAAACATAGGGGGCTTAATTGCCCCTTTACACG
341 ATAAATAAAGTAGTCAGCCGCATCGTTAGGGT
342 CCCATGAACACGGTTAGCATCTTTTCGACACC
343 AACAAGACCTCCCGGCTTTGTGGGTCAAGTAAC
344 GCTACCTCACATAGGTGGCCTTTTCCTTATGT
345 AAAGCAGGGGATGCATAGTGTCTCTATCTAAA
346 CCGTTGAACGTGCTGCATCTTCTACCTATACT
347 GTTCGGAAATAATCTTCTTAACAATGTCCAAT
348 CAGAGTTTCTACCAGCTCGTTTATTTGCTGCA
349 ATTCCTTTATTGTCTTGGTGGCAATCAGTGCA
350 ATTGTGGAAACTAAATACAAGACAATTACTAA
351 TATTACAGCTATAAGGACCCAAGGACGGGTAA
352 CTGTCGTATTCGGTAGTCCAGGAACGGTAAAA
353 GGTACATGTGTTCTTAAATTTCTAGCGTTTGC
354 ATCGCAATCTATATTAGACCATCTTTCACAGT
355 TCTCGCTCCTCCCATTTATATTTAGCTGGACA
356 CGACTGACGGACGAAGACATTAATTTGATTTG
357 TTATCCAGTGTACGAACAATAGAAGAGATTAA
358 TAAAACGAAATCACCCTTAGAAATACGTTTAG
359 AACAGAATGTAACAGGGTGAGTGATAATAGTT
360 ATAAATCTTTTAATGTTACCGTAATTAAAACA
361 GGGTTCATATAAAATATGGTTTCGCCTGTATCT
362 GATTGTGATTCTTGGTTTCTGGCGAGGGCGTC
363 AAGAAGTTATCGGCAAAACTTTACTTGTTCGT
364 GAGCGGCGTCAACAGCAAGTTCATTGCTTCGC
365 ATGTGGAGTCCCTCTCCGGAGGACACGATGTA
366 TTGGGCCAGATAGGGGAGCGTTTACCGCCAGT
367 AATGTCTTTCAGTCGTGCCACTGCTTTGAGCG
368 CAAGATTGCACGCGATTTACGGACGGCAGGTG
369 ATGAAATTAATCTCTTCTATTGTTCGTACACT
370 CGGCACAAATGGATTACGTCAAGATGTCACAT
371 GTTTCTTGAGCTGGTTATACAGATTAACCCAA
372 TGGCATTGGTATATGTCCATTCTTTATCCGTT
373 TCATATTTCCAACATCCATTAAATAATCTGGA
374 CATGGCGCTAAATCTGGCTTGGGTATGTTCTT
375 TGTAAAACGTTTTTTTTAGGTCGTCCAATTGT
376 TTGGTGGTATGACACCTACCGCATTCCAGTTA
377 GCTATATTCTCTCCGAGAAATCATTAGATAGT
378 GGTGGCCCGGTGGCGATTCCATATGGTGAAAT
379 ACTGGCTCGTCGAATTTAACCGCTACGCCGAC
380 ATTTGATTACATTGAGGATGTTGTTATAACGG
381 ACCGGTTTTTGCTCCCCGCGATTTACAGTGCT
382 GCATCGACGTTAACGGTAACGTGCGGCGCATG
383 ATAACTTCAGTATAAAGAGCACAGCGTGTATA
384 ACCCATATCAATGTGCAGTTTACCGAATTACT
385 ATAAGAAAGAAGATACTATTGCTTCTACTTAT
386 CATCGGCTATTTCTTCTGCGTTTTTTATCGCT
387 GGGTTATTACTGCGTGATTACTTCGCAGCCAA

388 ATTTTCTTTCTCCGGCGCGGCGTAGAGCATGTC
389 TAAACGCTGCTTGGGCCTCTGCCATTCGCTGA
390 AAAAACGATTGGGCAACTTGATTCTTGATGAT
391 GAGTATTGAGCCGGTAAAAACTTACGCCAACT
392 AGTGAAACCGGATGTTTATTCTGGCGGGGAAT
393 GGTATGCTTTATTGGCCTTGTCAGCTAATTCA
394 GAAATGTAATCACACTCTGTAGTTTCGGAGTA
395 TTCCCTTTCTATTACTTGTGCGTGCATCCAGC
396 TATTGTGATAAGCGCTTGCCCAAAATTGATAT
397 CAAATCCTGAAAAGCTTGAGTCTGATTCTGAA
398 ATGAGGGGTTTTTCTTATTCCGGGATTGTTACG
399 AAATCCGATTGTGATTCTTGGTTTCTGGCGAG
400 TTCTGAGCCCGACAATCGAACAATTAAGCGCT
401 TGCATCGGTCGTAGAATCACTGTCTCCGAAAGT
402 TCAGGGGACTGGCGAACAATGTCTTTCATGAT
403 GAAAAGGTAAGATGGGCAAGCTTCTAGTAGTT
404 ATTATCTGAATGGCATTTTCTTTGGCGCAGAT
405 TCGCCATTCCGTGAACCTGAGCGCGTTCGCGA
406 ATATTCTCGAGCGATAGCAATAGCCATTCCAC
407 TCGGTCAAACAAATTTAGGCGACGATTTAACA
408 AAAAAGAATTTGGGATTAAAGTTACCCATCAG
409 TCAATGCCTGAATCTCTGGCGTGATAGCTGCGG
410 ACGTCATCCTGAAGGCTAGGCAGCTCGGCTTC
411 TCGCTTTGTATTTCTACCATAACTATCAGCAA
412 ATGTTCAGGCCGGATAAATGTAATGTCGTTAT
413 TTTTGGATAACTCAGTTTGTTACCGTTGGCGG
414 CGGTCATCCCGAGTATGATGTCGATACGTTAG
415 GAAATTGTGGGTGTAGATGTTGCAGACGCCTC
416 TCTGACGTTGCCTGTGTTGCCGCTCTCGTATT
417 AGTACACCAAGTAGGCCGGTTAGCACCACCAT
418 CATTCTTAACGCCCCGCTCTGTTAGTGACAAA
419 TTGTAAGCCTTGGAACTCTGGACGCATTTTTC
420 AGCAATAAATCCCAAGGGGACAGCATGCTATT
421 ACGGAGAATCAATTCCTACGTTTACTCTCTAAC
422 AGATCGTGATGATAAACACACTTCCAACACAT
423 GGGTTGTCATGCCAATGGCATTGCCCGGTAGC
424 CAAATTAGGTGCGTGGGTTCAAAGACGGAACT
425 GATGAGTAATGCCTTCAGCGCATTTCTCTTCA
426 ACATCACTAGCGGTATCGAACAATTGAGCGAG
427 TCAGTCCCGTTATGGTGCTGGTGTTGCCCGTAAG
428 AGAGGGTATACGTATTTCCCAAACCATAATGG
429 CTCGGCATTGGTTTGTGCCTCTGACACTATCG
430 TACATCCTCAATATATCCGTTTATACGTTTAG
431 TTATCCGTGACCGACTCAAATACACGCTGGAACG
432 TCACCAATGAGGGCGACCATGCCGAGGACTTG
433 TCGTCAATGAATTGCGGGACGTTCCGGCG
434 CCATTGGTCACAAGATTCCATACCTTCAGGGG
435 ATACTGGCGCGAGTACCTTCGTCCATTTCATCG
436 GTGCAGCTTTAAACGTGTTGAGGTTGCGCTGA
437 CGTATGGAGCGCCACCATGGTGATAACAATCC
438 TGCCAACCCTGATATGGTTGTAACTGCCAATA
439 GTGATTTGCGGGATTTCAATCCAACTTGCGA
440 CTGACCGTTTTGCTGTAGGTGGCGGTGTAGGG
441 TCGGCGGTTATGCGGATCATCCCGCTTGGGGCG
442 TTGAGTAGCGATTTTCTTTGGCTTTAGGCACA
443 GAACGCAAGAACCTTGGCGACCGCTGATGTGGAG

444 TCCAATACTGACCGTTTTGCTGTAGGTGGCGG
445 CATATTAATGGCTAATAACGACATTACATTTA
446 TCGTTCGTTAATGAATTCAGACCTCACATTGA
447 CCGAGAGTTTGGCTGGAACAGCTTTTTAGTGA
448 AAATATGCTGGTCAGGATAAAGTGAACCCAGG
449 AGTANCGGTTTTCGCGCCATCTTACCGTGGTGT
450 TTGGCGGNGTTATCCAAANGNTATCACCGCAG
451 TGAGTCTGCAACACGTTGAATAAATTCCTGAC
452 CCGAACAGATTACGGAGTTTATCTGCACCTGG
453 TCACCGTGAGGCATTCAAGGAGAATCCAAATG
454 GGTTTACGCCGCTGCAATGGCTCAACCGTTCC
455 AAGTTACCGGCAAGAAAATTGGTGCTGGTGAA
456 ATTCGCTATATGTAGTTCCCGCTGGAACTTGT
457 GAAAGCACCGACAATTGCTTCGATAGCTTTCGG
458 AAGAACAGAGGTAGATGCAGCCTGATCGCCAG
459 GCTCTGCCAAGCTGCAACAATCGCGGCCAACA
460 TGAAATAGCCAAAGAATTCCGCAAATGGGTGC
461 CGGTAAAAACAGCAACGCAGGCAACTGGCGAT
462 CGTGATTGGCCACTTGGCTGAGAATGATGTCA
463 TAAAATGGTTAAACGATACGATGATTGTGGAA
464 TTATCGGGGTCGGTTGCATCACTAATGACATT
465 TGTAAGGAGTGGCTGTGTTAACTGATAAACAG
466 TTTCGTCGTGACCCCACGGTGACTATCGTCGA
467 GCCGTGAGATGTGTGATCGTGAAGTGGTTTAT
468 TTGGGCTGAGGTCATCGTTGATCGCTTGAGTG
469 GTCCATTGCCTTAATCAGTAACGGCTCTGTTT
470 TTGTTGCTAGTTGCATGTTTTTTCCAGCTATTT
471 CAGGAATGTTGGCCGCGATTGTTGCAGCTTGG
472 CTAGAGAATAGCTTTAAATTGCTTATCGTATA
473 TCTGTACGCATACCGCCATCTTGCATCAGTCT
474 AGCAAAAATCTTAATTACATCTGATGATTTCGG
475 TTTACGGCACGGCGAAAGATTCGGTTCTTGTC
476 AACGAACCCACGTAGAATTGCCATCACCGCCGG
477 AGTGACTAACACGTCACAAATGTCGCCGTTCTN
478 TACGGTTGCCGTCAAGGTTTCAGTGGCAGCCA
479 GGAATGACTCAAGCGGGATATCAACTTCAACA
480 GATAGCATGGACGTCAAGGCACCTGCTGGCTAT
481 CGCACGCAATACCGGATAAACCGGCTGACTGT
482 TACCGGTGGGCGCTTTCGTGGCAACTGGCAGG
483 AATTTGCCTGTCTCCGTTGTTGGAGTGAAGAT
484 AGTAAAGGCCTTCATAGTCCGGTACCACATA
485 ATCGTAAGCGGGCCAGGCCACATGCGGTAACG
486 GTGATTTTATTCTGCGCGGTAACCTGCTCACT
487 GTGTTCGCCGGCCTTTGGATCGTCATGCAAAT
488 GGCATTTTTTGAACGTCTACGTACTGCCACTA
489 GAAATGTCGTCACAGCCATCCAGAACCGTGAT
490 TCAAACCTTGTCAGTCCCATTACGCCGGGAGT
491 ATCGTCTTGCTCGGTGAGGTATCGGGCGCACA
492 GGATCGGTCAGTCGCCATCTGGCCCCCATAAA
493 GCATCATCAGGATCTATTTCACCATGCTCGAT
494 TTAAAAGACTTACTGGACATAGGCATTACCAT
495 TGTTCTGAGAACGCTGCATGTCATTGCCTGGGG
496 CGGTTAATATTTGCTGTGCCTGGTTTGATGAA
497 CTGAGTAAAGAAGAATTGCAGGAAATGATAGT
498 CAAGCAAGTCGATGTTGATAGTCCCCTCTGCG
499 TTAACGTAGCCAGGGCGTGTGGAACATGCCTAGT

500 TTTTCGGCCCGACATTACGCCAGACGCGACA
501 CGGACGCGGTGAAAACTATCCTGCAACGATTC
502 TATGAGTGACAGCCGTTTTCACCACCGCCGTG
503 TAACGAGAAAGTCTTAATCTTTAATTCATCAG
504 TGGGACGCTTTACAGTCTGCACGTCTCTGAGT
505 TGGAGTGGCGAGTTAGAGAGTAAACGTAGGAA
506 CTGTTTTCGCGCCGCCTCGGTGCTATGCCTATT
507 GTTATACCCCGCGCAGGGAGTGAAGCGTTGAC
508 TTAAGTTCTTTTTGTCAGCATCTTTAATAAAT
509 CTGAAATACAAATAAAATAAATCGTCGAACATA
510 GAGGTGTTTAGCAGGGAAAATCTGGAGGTGCT
511 TAACGTCGTTTAATTCTAGCCAATCGCCATTT
512 ACAGCCCAAAAGGCGGATTTTATTCGCGCAAA
513 TACATCGCATCAACAGCTTGCTTGTTCACTTG
514 TTGGCAATCATGTTTGGCTGCGCTTGGTTAAAC
515 TGTCAGGCTGGGACTCTGATTTTTCAATTCGT
516 AAGGCCACCCTGCACAAATTAGCTGTTGCATG
517 GGCAATTGGAGCCCTCTGGACTGGTAACTCTC
518 TTGGCGTTGATAAATTCAATATATATGCAATT
519 TCTTTCAATGCTTTCTTTCTCGCCTTTTGTCC
520 CATTAATGGCATCAAGAGCGATAGATTCCGTT
521 TAAACTCTTTACTCATTAATCAATCCTCCGGT
522 ATTCAGACAAGATTTTCAAACGATGTGTTTTT
523 TAAATTTGCGCCATTCTTCTGCTGTGTGGTCC
524 GTATGGTTGATCCCCCACCGCCCCCTTGAGTT
525 AGAGCGACTTAATGATGAGGCAATCAGGTTAA
526 GCACCGGGGAAACTTGCCCACAATGCATCGCT
527 GCGTCAAGGCCACCGGCACTGTCACCCTGTCC
528 TAGACGCTATCGACGGGGATTGCCATTTTTTC
529 GTGGGTGGTACCCCGGCAGCAACGTTGCCGTCT
530 CTTTCTTCAACTCTTCGATGTTATTCATCAGC
531 AGATAAAGCCATTCTGAAATATATCCCAGTAT
532 TATAATGCGATGAGTGATTTTGAGGTTAGCAA
533 CTGATGTGGGTCATTTATTACCTCTCGAGTCGA
534 CTTACTGATGCCAGGAATGCCGCCGCCTGTCA
535 AAGTTGTGCTCGGCAAAGCCGTTGGTGTCTCC
536 TTTATCTGTGGTCGAGAGGTGCCAGCTATAAC
537 TTATCCACTCTGTGATTACCGAAATGGGCGGC
538 GGGCGGGTAGAATGAAATGACATTAACGAGAT
539 AACGATCGCTATGGAATGGGTAAGCGCCTAGGA
540 TTCTCTATATTCCTAAATTTGAACGGGTAAAT
541 GGCAGCAATGCTAAATAACGGCTGTAAATTCT
542 ATTTCAGCTTGTCATTGATGGACCTTTATCCG
543 TGCAGACGAGCCTACAGCTGGCCAACTATTAGT
544 GAAAAACATCTTCCATTACCTTATATTACAGTG
545 GAAAACGTTCTACAGTTTATTGAGTCTGTTAA
546 TGAGCCATTTGATTAGATGGATTATATAATCT
547 AATCTTTCTTTATGATAAAAATTGTATTCAGA
548 GTATAAAGTAACATTTCTCACTAATAAAATTT
549 TGTAATGCATCGGTAGGTGATCTTCCCTCTGA
550 ACACGCCAACACTGACTTGCACCGTGCCTCGTG
551 ACACGCTCTGGCGTTTCATTTTTTGGCGTCCT
552 TTTCCCACAAATGACTTATCCACATATCTACA
553 TCTACAGGATTGTTTTTTAATGCTTTTTATTT
554 TCTATCAGGTCGCTTTCCTCTTGTGTCAGAGG
555 AGTTGAGAGGCCATCAAGCCAACTCAACGAAC

556 GAGAACTATGTGCATCTGTTTTATCCGTCAGA
557 TTCAAAATCCCATCGTGGAGCGATTACCGCAA
558 CGTTAGGTAAACTGTTTTTAAACTCTCTATAT
559 CCAGAGCCTCCTGATACATCTGATCCCACAGT
560 CATCACAATGCTCTGGATGACGCAGTTCATCA
561 TGATTTTGATTATCCTATGCCAGCCGAATATAG
562 TAGCTATTTCACTTGTGAAACGTTTCTCATAT
563 GCTCTCCCTCCTCCTCCTCGCTGTCGCGAGGA
564 GAGCTGGAATTGATCGCTATCGCGTCCGGGCT
565 GGAACTGGCGGTTGAGGCTATGCATACTTATT
566 ATTTTGCTATCCGTGAAGACCGCCAAGAGTTA
567 ATGTCGGCGGCGGGATGAGTCACGAAGAAGCT
568 CCCTTCGGTGATACTATGCAAGAGATTAGCGT
569 CATTAAAAGCATTAAATCTAGAAACGGTGCAA
570 ATGGACATAACTAAATCGCGGGAAGAGTTTGA
571 CTCTCAAGCTCAGATATGCATTTATCAATGTC
572 TAGGATGCATGAGGCATGGGATTTCGCCAAGG
573 CATTGACCTGTGACTCCTGACGGGTAGCGAGC
574 TGCTCTTCCCACACCGGGATATATTTCAGTAT
575 TCTAAATGCGAACTGAAATGTTGTCACCAATG
576 AGTGAAGGAAGAAATCGAGGATGTGCGGCAGA
577 TGCGGCACTCCATCGCTCAATAGAGTTAGCTG
578 GAACTCTATCTGCTAGTTCATCGAATGATATT
579 TAGTAAACAATACTTTTAGTGCTTATGCAGCA
580 AAATATAAATGCTAGCAATCAAATGGCAGCAA
581 ATAGCAGTTCAGTTGCCATTTGCGTAATTATT
582 GTTGTTACTGATTCGCTGATTGATGTGTAATA
583 TGCGTATAGCTGGACTGGTACTGTTGATGTCG
584 GTGCGTAACGTTATACATCATTTTATTTACAC
585 GAGGTGTATACGCGGGTTTTTGCGCTCGATGT
586 ATCAAATGGCAGAGCTTGCTTGAGCCTTCCGA
587 GGGAAGTGTTTAATCATCACCGCTGATGTATC
588 AGCGCCGGGGACATTTGGCGGAAGAATTGCGA
589 CAGAAGAAATTTTGTAGTTTCATTTCCCTATAG
590 TTACACAGGAGAGATTGACTAGCAGGTAGATT
591 TTGATTGTTCATCAGACTGACGTTGCACAATTT
592 ATCATCACCATCACACACACCATCACTATGAC
593 ATAGCATGACGGATATGACATTGATTCCAACT
594 TCGGTCGTGGTGCTGGAGTTGGCGCTGCTGAA
595 GGAATGCAGATGCTGAGTAAAGAGCAGTTGGA
596 TTATAGAATTGCTTATTAATGAATATGTTG
597 AAGAGCACGATCATTTTAGCAGCGAGGCCCAG
598 GCCGCCAATACGTTATAAACTTCATCATTTAC
599 ATTGAGTTTCACTATTGTACCCCTGACTGGGA
600 GAATTAGAAGCCCTAAAATAAATTATCAGTCT
601 CTCTCCAGCCGCTCAAGTCGCTGGGCTAGCTC
602 TTTAGTGATAAGAAGCCATTTGGAACTGTAAC
603 GTGACACCTGAGGATTTTATCCTTGCCCGAAA
604 TTAAAGGTACGCCGGGGCCTTGGGTTGATGTT
605 TTCCCTGCAATTCTCGGACGTGAATATCCCGA
606 AGTCATTACTACCTATAAAAATAAAAGGGGGT
607 TCGGATATCACAAGTTAAAAGCGGATTGCCGT
608 CCGTTTTGCCCGCTCAGCAGGGTCATGCCCAA
609 CATGCCCAAGGAGTTCTGGCCCATCTGCAATT
610 TTGCGTTGACCGGACGGTTGTCTGCGGCGGTT
611 AATCAATCTTGTTGAAGCCATCTTGTTTTAT

612 GTTCCTGCTGGGTGTCAGCGGCGAACTCGGGC
613 GCCTGGGCTAGCTGAGTCTGGCCTGTCTGATT
614 ACAGGAAATCCGGGACAACAATTCATTCAGCA
615 AGCCTGCTCATATTTCCTGCATCGCATAAGCA
616 CAAATCATTATTAGTCCCGTGATTGTTTAATG
617 TTAGTGCCGGCCGCAGTGGTAGCAAAACCAAA
618 TCGGTAGCCAGAAAAAGCTTGCTGACGTTTGT
619 ATGCGGGTGAAATTGCGCCCGTAAGACTCTCT
620 GAACTACAGAGAGAAGATATCAATACTCGTTT
621 TCAATCAGTTCTTTCTGTGCCGCTTCCAGTTG
622 ATTATCTAACCCGCCAGCTGCCATTGCAGTGT
623 ATTAGAAAATTAACCGGCATGGATTTCCCGAT
624 CATGAATGGCCCATCGAGAATATCAGCATATA
625 GCTGGCGTTGTCCGTTTTTTACGCTGTTTTGG
626 ATTAAACAAAAATATGAATCAACACTTGCCGAC
627 GTCTCTCAATTTCCAACCGCGGACGAACTGCC
628 TCTACATTTTTACCGATAAATCCCTGGGAACT
629 ACCGTAGCCGTATAAGTAGCGGTCTTGCCGTT
630 CCGCATAGCCCGTTAGTCGCACGCTTCGAGGT
631 AATTTCTGCACGCTTAATGACTTTTCACCCTT
632 CCACTATACGCCACCCCACGGTTGAACTCTCT
633 TTTCTTGATGCAGCAAGAGCCGCATGGGGACCG
634 ATCTATCTCCAGTACTGACGTCTATAACATAAC
635 ATTATTAAAATGCAGAACCGCACAATCAGTAAT
636 GAGGCTGAAAACCTCACGATGACCGAGTTTCA
637 GGGGTGAAGCATGAAATCCGTTGTCACGCTGA
638 GCGCAAACCACTCCCCACATTCCGGGCATTC
639 TGAGCCTCGGCCGCTGATATCGCCGCCGCTTT
640 GTGTATGACGCAGTGGATGAGATGGCTGACGA
641 CCATTGACCAAAACAGTCTAGCTATTTGCTCA
642 CCAACTGATGCGACTGCTTAGTATTAATGCGC
643 TTTAATTATAAATAACATCACGTTACGCTTAT
644 CGGCCATCTTGGGTTGTCTGGGGTATTAGCAA
645 GTTGTCATGATTAACCCTGCGACTGTTTGATG
646 TGAATGTGTATGATTCTGTAGATTCAGATGAT
647 CAGCAAAACCGATTTCTACCAGCGTTCCCTCT
648 ATTAAATCTTTCACTTTCGTTAAACGGATGTGG
649 GCGGTTTATGCCCGCGCCAAGGCGGATTTGTT
650 GCGGCACACAGACCACCGCTGCCGGGGCTGAT
651 TTGGCGATGAATCGGCAGTTGATATGATTATC
652 ACTACATGGCTAGCGAAAACACACCAGCGTTA
653 CGCCAGATGATGACGATGATGATATGGAGTTA
654 AAGAAAATTACTCGCGGCATGAGGATTGATGA
655 GTAGTTGTCGCGCCTTCTGCAACAGTTCCAGT
656 GGTCGCGGCAGGCTAACATCAAGCATCATAGA
657 CGATCCCCGCACCGGCACCGGTGCAAGATGCT
658 AAGGTCCATACGGCATCTTCATGAGCTTCATT
659 CTATGACCATGAAGATGGCCCTGAGTGCATTT
660 AATGTACATTTCAACTTACCCTCGTTTGAATG
661 TAGCAGCGCGTGAAAAGCTGGAATGTGTCGTT
662 TCTGAATATGGGCGCATCAAGTTACGCATGAT
663 TTTATGATATTCGTGCTCGTCATCGTCTTTGT
664 GTTCTCCATTCTCACGAATGATTCTGGTCATT
665 TAAATGACGGAATAACAACCTCGGTGCCGTCC
666 TTTTACACCAGAATCAGCAAGCGCCCAAATCA
667 TTGAGGCGGCTGGCACTCCATCGGCAGATGCC

668 CATTGAAGATAGATAAAGGCGTTGATATTGAA
669 TCGAATAGTCAATTAGTCGCACGTTTCGAGGT
670 AGAAAATGAGCGAGAAATATTTACCGAATGTT
671 GACAGCTGGGTACTCGTTACCAATCGCTCAGG
672 TCGCCGAGCGTTTTCTCTTTGGCGTAGTACCA
673 CATTTTCTTTAGAACAAAGCTGGAGTCCTGCT
674 CGTAGAAGCGAAGCTATTCGCCGGGGTAAGTG
675 CGGCTGCCAATACTGCTTTAACAATTCCGTTT
676 TTTTACAGTTAGCGAACATCATCAAAGCAGTT
677 GCGCTTGCAGACATGAGCCGTCCGATTGACTG
678 GAATTAGGTCAGCCGGGCGAACCTACGCAGCC
679 ACTTGAATCATAGCCGTTAGAGCATCTTTAAC
680 GGTGACGGTCTTGTTTGCTGTCGGATACTTCT
681 GCCACCTGAGCATTGATTGACATAATTCATGC
682 ATTAATATGCAATTGACGACTGGCATTCTTTA
683 TAACTCTATCTACTAATAGTTGGCCTGCTGTA
684 GTTTCACATTTAAAGAGGCCCAATCTTCAACT
685 GGTAACACTAATACTGAGGTGATGGAATTTGCG
686 GTTACACGTAACGCTAGCAACGTCATTAAACA
687 CGCTTGCCGAAGCCCTCGCACACAAGGGCAGC
688 GTGAACGAGGTGGAGACAATAAATCACGTTTG
689 CTAGCCCCGTAGCTGATGGTCTTTCTTTTGAA
690 CGTAATGGCGGGTTTGGTGGCTTCGGTAACCGT
691 CAGCGCATGAATATTTTGCGGCATGTGATGTT
692 GTTAATATAAAGGGCGTTACGGTTGAATGGAA
693 GCCACTATCAGCACTTCAAGAAATGGGGGGGT
694 ACTCATAAAACACAAAACCCCGCACGAAGCGA
695 AATACGCCAAAGATAAGCAGGTAGCCCGCTGT
696 GTTTTTGGACAGCACAATCTTGTGCCCCTCAA
697 TGTATTCCTCCATTCTGTTAAGACTTGGGAGAC
698 ATAATATGATTCCTTTTATACCGTTATTAATG
699 TATTCCGGCGGTGGTCTGAGTCTGGGCGAAAT
700 GGACGTGATGTCCTGCGCACAGATCCTGATGC
701 GCGATACCAGCCAATACCTACGGGGCCATTAA
702 GCTTCTAGCCTTGCGTTTACATTATTTTGAAG
703 GTAAATGAGCGTTATTGACCCGACCTTGATAT
704 ACTCGATCTGCACACTGTTTGTTAGTCAACCA
705 AAAGTGGGCTTCCGTGGTGGATTATATGTAAC
706 GGCTTTCTATGTCGCAGATGGAGAAGGTCGGC
707 CCATCGTTGCAGCAACTGCGGCCGATGATTGA
708 TCATGCGTTGCAATAATGCTTCCTGTCTTCTT
709 CATTACAGTGCGACCCTGCTTGGCTCATGGAA
710 AACGCAGCCAGTGCCAGCCAATCTTCTTCACC
711 GTGCCACGCTGTGCCGGCATGGCTGGCACATT
712 TCATTTTTACCTTTTGATTCTGAGGGATATGA
713 TAGCAATTTAAATGTGTCTGACCAGCAGCAAT
714 AGTATTGGGAGTCTATTGGTGGTACTTGGGGT
715 TTTGGGTAAATGGGGGAATAAGGGTGTCTATA
716 TTAACTTCATCGCTATTAACTTGCGCTGCTTT
717 ACTTATTTCATCGTCTACATACTGTTTAACTT
718 GCTATATCGACCCAAGGACGCTGGCAGATGAT
719 ATGAATATTCGCCAAGCCGGTTTCACGGTAGA
720 ATCCCCTCAAGGTTATTGACGGTATCGAGTAG
721 CATAGAAGCATTATTAACTTTGCGAGTAATCAG
722 TTTGCGCTTGTCATCCAGCTTTGAGGTTGGGT
723 TAGGTGAATATTGGGAGTCTATTGGTGGTACT

724 AAGCCCAAGAGGAATTAGATCGGGCATCTAAA
725 TGTATCATGAGCCTTTAATTTCATGCCATCTG
726 CATGCTTTGATGTCCGGACGCATATATTGCTT
727 CTGCGCTGCGAAATCAACCAAGTTGAGCAGCT
728 TGGGTGCTATAGCAAAGCGCTTTCAGGCTATT
729 AACGGATCGCCGATGTGCGGGGTGTGCGGCGG
730 TAGCAACATCTGGTCAAGTATTCGCTGCAACT
731 TCTGTACTCGACACTCTGAAATTGATATATTA
732 AATCTAACGATGATAGTGTTATTAAAGCGTTA
733 GATGTATTTAGATATTCAAGAATAGACGCATT
734 GGTTATGCATATCTATTGCTCACGTTTTAAAT
735 ATATAATAAAACTCAATTTAAATTAACTATAA
736 AATCCTGTGCATTATGTGACGTGTTAAAATAAG
737 GTATTGATAACTTAAAAGGTATTACTGACATGG
738 TGTTAGTAAAACCTGAGCATAAGCGTCTACAC
739 ATGAATGTATTCCTCCACTCTATTAAAACTTG
740 TTTCACTTGTAAAACGTTTTTCATATGGTAAA
741 ATATTTATAGAAATATCCAAGTTTATCATGAC
742 ATTATCATATAGATAAACATTTATTTGTTGTT
743 GAGATGGAAACCATTTTTCTATTCAACACAAT
744 AAACTAAACTTCGCGTAGGTTAAGGTTAAATA
745 ATTTCTTGTTGTTAGAATTGACGTAATTCAGT
746 TTATTGGTTCAGCCTTTAGCAATGGCGGACTT
747 GCGGTCTAGCACTGCTGCGTAGTTGTCTATTT
748 TAATCGTACTGTTTGCAGTTTTGATGACGAGT
749 CTGATAAATTGCGTAAACACATGCGCGATATG
750 TCAGTCTAAGATAAGCATTAGCGCGTTCAATT
751 GCTTGGCGGCAATATAGCAAGGTGATCCCATT
752 ATGCAGCTATGGCTAAGTTTAAGTCATATGA
753 ACCAGTTGTACATGTGTCAGTTTGTCGACTCT
754 GTAACGAATGCGTGAAACCTCTAATCGTGCCT
755 CTGCGGAGTATGCGCAGATCCAAATGGTGATT
756 TATCTGAGATAGAATTTCATGATTCAGATTTG
757 GCTAATAACACGTCTGGTATTCCAGATATGGAG
758 TGGTCGAATGATATACGCCTGATGCGCGAACA
759 TTAAAAATCATAACTACCTCATTTCAATTTAG
760 AACCTGCAATACGGTTGATGTTTATTTGTCTC
761 AATGAGAATTTGATTTAGCTTTAGGTCTTGAT
762 CATAAATAATCAAGTCCAAAGCCTTTCCATTT
763 AGATTCCAAGAGTCTTACCGCGCTGTTGTCAA
764 AACAATCTAAAATAAACGCTCTAAAAGGTATT
765 TAAGCGCTGCCGCTCTTATTAGGTCACTGCGC
766 AGCTTTTTAGACTTTAAGCAAAGCCTTGACTGC
767 TTGGGCTACGTGATAGCGCTTCACGGTAAAGA
768 GCACTATCGTAGTTCATGTTCCGACAGGCCTA
769 TCACCATGCCACACAAATTCCGCGCATATGTT
770 GTAGCTCCACATTGTAACGTCGCCATATTCAA
771 ATTTAGTCAAGCTGACTCATGTTTGGCCCGAT
772 ACCATCTATCAACTCACATAAAGTCACAGCGA
773 CCTCCCATGCTTTTCAGTGCCAGCACCTTCAC
774 CGCGTCCGGCCCTGATCCATACTGCATACTCAC
775 GCCAACGCTTTCTCTGCAATGTCTGCGTGAGT
776 CCATGCATCAAAATCACTTCTCGACTTATCCA
777 CCCTTAACTGGGGCTTTTTCTTTTGTGATTGT
778 TTCTGACTTCGCCAATGCATCGCCTTGCATTG
779 AGACGTTGAGTATGAAATACATATTCACGATT

780 TGATGTTTGCCATTGAAACACTTCTTATCAAT
781 GGAAAGGATAATCAGGAAGATATTCACAACTA
782 ATTTCAATGGAAAAAACAGACGGAGAAAAAAC
783 CATATCATCACCGCGTTGTATGCACTCTTCGA
784 ACCAAGGTAGTGACATAACCGGCGCGAGCATT
785 CTGGGAAAACGTTTTAATCGGCGCTGGCAGAC
786 ATAAGCCGGTAGAGAATGAATACATCCGAATT
787 GCATGCACCCGGTCGAAGTCGCCATATCTATG
788 TGTCTATCCCATCGCAAGCCAATAACGCGGAA
789 ATGGACGGCGTTTAGTGCTGTTTATTGATGAG
790 AACGAAAAAAACGCCGGTAATGCGTCGATTGT
791 GTGATGCTAGTTACTGGAACTTCACCCTTTGG
792 GAACTTGCTTCTGCTGTGGTCGCGCCAGATAC
793 TAGAGGTAAAAATGAAGCCAATCACAAGTATC
794 CTTTGCTCATCGTCTTTCACCTCGTTCCTCTT
795 TATGTAGCTGAAACAACCCGCTCAATGTTTAT
796 TGTTTTTCAATCATTCGTGACGCATATCATTT
797 ATTTATTACAGATGTTCGTACAGGCGCACACA
798 ATTACTTGTCTGCGAGTTACAATAGCCCCCAA
799 ATTACATCAGTAGTGGGAACGGCTAATGCCAT
800 ACCGGTTTCTCTAACCTTGGTTTCCTTATTCA
801 ATCAGCTACGACCGCGTGACGAATCGTGCCAC
802 TGCCATCTGAATACAGTTTCTTAAATTGCTCT
803 CTTGGCGTAGATGGCAAGCCTGATACCATTAA
804 CCATTAATCCGGGATATCTTCTACGGCACAAT
805 CCGGCAATGAAGCTGGTGCGGCTGGTCTTGGT
806 ACTTACCTAAAAGAACGGCGCTTCTCTCAAAG
807 ACCTACCGCTTCTTGTGCAATCGTTGCAGCAA
808 AAAAAGCACCGTATAATCATCCTAAAGTTGACG
809 CTTTGCTCATCGTCTTTCACCTCTGCTTTCTT
810 AGTTCAGCATTGCGATTTTTTGCAGCAATAGT
811 TTTTACTTCCTCTTAATGATTTTTCGCCTTCC
812 AATGGTTAAAGGCACGGTATCTGGCGTTCAGT
813 ACGAATTGCTAAAGACATTTGATGTGATTGCG
814 ATGCATCCATTAGTTAGAGTTGGAATGGTGCT
815 CCTTTTTACAGAATAAAACTGATGGGGCTGCGT
816 TTAATCAATTCCTGAAATTCGGTCATCGTGAG
817 TGCCCTCAATTCATGGAGCAAAGTCGGGCCTT
818 ATGACTTGATTAATCTCAAATCCATTCAGCGT
819 GTGTTGCTCATAACGAACGTTGCCGTTAGGGA
820 ATTCTCGGGTCAGTATCGGGCCGCCCCGCCTG
821 AACTTGCTTGATGCTGGCGTTGAGCCGCTCAA
822 GCGCGAGCAGCTTGAGTGTGGCGTTGCGGCAT
823 TTTTGGGCTTAAGTATGTTGGTGTGGCTGAGT
824 GTTACGATCAATGTAAGTCAGTGGGTTCCAGC
825 ACACTAACACCTTTACCAGACCGCGTAGGTGC
826 GCGTTCAAAGCGTTTAAAAGACACAACTTATA
827 TTACCACAGGCGGCAGCACCGACACCCCCGCA
828 TAGTGAGGAACCGGAGAGCATTCTGCCTGGCG
829 CTAAACCATTTCCTTGAACATGTATTTCAACA
830 TCAAAGCCATCATCATGCCCTTCGTTGTCGC
831 AGAATTATTACATAGATAATATCATTGACTCT
832 GCATTATTGCGTGACGCCGGATTCCACGTTAT
833 ATCTCAATCATTCGGTTATGGCTCCAAACCAT
834 TTGATAAAGTAGGGTATGTATCAATCGGTAGC
835 ATCCAAGTTTATCATGATGGTATACATACATCG

836 AAGGATCATGATGATGTCTCCAGGCTGTAACC
837 CGGAGCCGGAAGAATTTAAATCCCTACCTGAC
838 CTTTAGCGTTAGGCTTAACGGTGACTCAGCAA
839 TGAAAACTCAGGATGGGAGCGCAATCGTGGGA
840 TAATCAGTCTCGGAAACGATAAACCCAATCAT
841 ATCTAGTGCTCCTTTTGGGTCTATATCCAACA
842 TACAACGGTGTTTTTAGGCGCTTGTATCTTAG
843 CATCAAAAGCTACAAATTCCGAACCTAATGCA
844 CTGAAGGTTCACTTGGAGCCGCATTTCTTCGC
845 CTGTTTGAATCGTTACTTGTTTATCTGTTGCT
846 TTGAATAGTCACCCTCGGCAAGTTGCACCACCT
847 TCTATTCTAAGTTCCTCGTTTAAAAACTCTAAT
848 CGGCACAAATGTATTTTGAGGATATGCCTCAC
849 CTCGGCCTCATTATCCTTTTCGATAATCTCCG
850 ATCATTGTTGAACTTGCTTCTGCTGTAGTTGC
851 ACTATGTCATGACGCAGGGCAATCACGGCCAC
852 AAAGGGCTGATTATTACAAGCGGTTCGTCCAC
853 ATCCTCCGGTGGCTGCGGCAATGGTTGCCAGT
854 TTTGAATATCCTCCGGCGATTCAGGCTGTCCG
855 CTGCCAGCATTACAAGATGATGGCTGGATTGT
856 GAAGATCCTGACGCAAGCGACGATGGTGATGAT
857 TGGCAGCGGCGGTATTGCTGTCACCGGGATTG
858 TAAGTGATGATGCAATTAGTTTATCTACGCCA
859 GTGGTGATTGAGAATAAGAAGCGGAAAATTAG
860 CAAATGGTCCGGGTCGTAAAGTAGTTGATAA
861 ATTTGGCGGAAGAATTGCGAGATGATCCCATT
862 ATATATGACTAATTATTCCGTTACTCAGGTTAT
863 AAAAAGAATTTCTAAACAAAATCGTAGCAGAT
864 ATAATCGTGAATTGCGAATAATCGGTTTCACT
865 GCATACCGCTGATGGGAACTCTGTATCACATG
866 GTGTCTCTATCTAAAGGTGAAATCCACGAGCT
867 GATATTGCAACGCATCTATCACTGGCCGAGTGC
868 CTCAATCAACGGATACGAGGGCAGATATTCGG
869 AACATCAATCTTTTTAGACCCTGCCGCTTCAT
870 GACATGATTGGCTTTAAATTCTTGTTCACTCA
871 TACCACGACACAGATCGATTGTCACATCCCCA
872 GATCGATGCGACTAAACTAAACCTCAGCACAC
873 AGCAGCCTGGGTGACATCAAACGTGATATCGC
874 CGAATCCTACCTTATGCTGGGTTTTCGTTGAT
875 ATGCACGAAAAAGAACACGCTGAACTCCGCAA
876 ACAATTTTCCCTACGCCATTGACTGAGTAGGT
877 ATTGAAGACCTTCTCCGGGATGGCCTGTGGTT
878 CTGGGCGGGGGATGCGGTCACGCGCGTATTAT
879 AAGAAGAGGCTAACTTTGGTGAGATTTTCTCA
880 TCTTGGGTATTGTGCCAACCGGCCAGCGCTTA
881 TGGTTCGGGGAATGCGTCTTCCATAACGCCTG
882 GCTTTAACATCGCGCTCAATTATCTTGTATCT
883 CTTCTACTTTCTTTTTGTCTTCTTCGCGCTTT
884 TCCTATTGACTAATGAGGCTCTTATCTCTAAT
885 TTTGCCGCTGATAAAGCGGCTTCTGCTTTCTC
886 GCGTAGAGCTTAAACACAACCAATGCAACATA
887 TCATCAATCAATCCTCCGGTAGTTTCGGCAGT
888 GTATGCGCTTGAAGATTCCCGGCGTAGTTAAT
889 GTTACGTGACAAAGCTGTGAGCCTGTCACTTT
890 AGTGCAGACCTTCTCTCTACTGATTCGACATA
891 GAGCAGTACCGGTATCTGATGACCATTTCAAT

892 TTTGAGTATCCACTGCAACGGTAGCGCCATCAG
893 ATCCGGGGTTCGAATCCCGACAGCCAATGAGT
894 CGATGGAATCAAGCTGGCGGCAAAGTGTTGCC
895 CGAATCCCCTGCAAACCCTTAATCTTTTGTTT
896 ACAACTATTTAGAATACAATTTATTCTTTAAT
897 TTAGATGTGAAGGCAAATTAATACAGAACACT
898 TAGCGTTTAAAGACAATCACGATTATTATAAT
899 AAATGGGTATACGCGCAAACTCAATTAATAAA
900 TCATTTGGTTTTATTATTGATGATGACACCAT
901 AATAAACAGGATAAATATGCGCTATCCAGATT
902 GCGTGGATGCGCTCTATTTCTCACATTAAAAC
903 GTGGCGAAACCAACCCGCCAGACTTGCACGGT
904 ATTAGAAATAAGGTCCATTTGCAGTGTGTGAA
905 CCTCAAGCCTCTCGGTTTCATCTTTTTCCTGT
906 AAATCTATTGCCGTGCTGATTGCATGTTTCAT
907 AATAATCAGGCAGCTAGCCGCCGAGCCGGAGAA
908 CCTTGCACACTACTAGCGTGATTAGCTAGCAC
909 CCCTCAGAGTGTAGTGAGTCAAACATAGAGGG
910 GTTGTAGCGTTATTAAGACCGGTGGCGCTAAC
911 TCGGTGTATTCGACCTCTTCACAGCCGAGATA
912 TAAAACGAGTCATTTTTGTAGTTGAATCGTGT
913 CAAATGGACTCAAAAAACCTGTTTTATTATCA
914 ATACAATCATTGCCAGCCGTAGCGGACTCTCT
915 AAAAATAGGGCCAAATAAAAATATTTCTGAGT
916 TTCTATCTGCGCTCTGTATTCGAAGCTGATAG
917 CACTAAAAGTATTGTTTACTACAGTATTTTTA
918 AACTATTCCTCAGCAGATTGACTGCCGGTAAT
919 GCCAATGTGATAACCCCGCATGACCATATCAA
920 GCGGGAGCTGGAATAGCGCCTATTCGTTACTT
921 GCATTGAGTGGCTTCAACGAATGCATGCGGAT
922 TATGTTTGCCTGAAGTAGCTAAGTTTGCAGCA
923 GCTATCAGGTCAGTGCACGAGATGGCCCAACCG
924 CATGGGCATCTAGAAATGTTTTTGATGTAAGT
925 TATGTCATTCATTAAATAATCTGGGTTAGTAG
926 GAAATATAATCGCACTCCGTAGTTTCAGAGTA
927 CAGTATCGATAAGTTTATCAACAGTAATAATT
928 GGCGCGGCTCCCTATATGCTGATATTCTTGAT
929 ATTGCAGCGGAAGAGGCTGGGGATATTCAAGC
930 CTACTTACGGGTAGTGGTGTTGTTTTAGAGGT
931 TCTTGATGCCTGCCTCGCCTGCTCGGCCTGCT
932 GCTCTGTCTCTTCCAGAGTGTCAAGTTTTCTC
933 GGTATGCAGTCAGAAGGCTTTGACCCGCTATT
934 ATCAATCACCAAGCCATTTACCATCATCTTCG
935 TTTTGGCTTCTGGTTCTTCCATTTTTATATTT
936 AATCCCAATTAGAATCATTAATTGATTGTCGT
937 TGTCCGATGTATGTCGAGGCAAGAGAGCAGCA
938 TGTGCTCCATCATTTCACTTGGTTTTTCGCAT
939 ACTCCCCGTCTACCGTGAAACCGGCTGGTCGT
940 GGTACTTCAGCCACGGCAGCGGCTGACGATAA
941 GGATTGTTCACCCGATTAATTTCTTCCGCAAA
942 ACTACCTTGAAGAAATCCAACACTTGCCAGTA
943 CTATTGAAGTGCAGAACGGCGCAATCAGTCAT
944 AAATCAGAGACGCTTGTTAATTCGGCGCTATT
945 TTATTTTGACCGCAGGGGCCAATCATCCGTTT
946 AGAGTTGTTGGTAACACTAATACTGAGGTGAT
947 GACAGTGGCGATGCCTGGGCCTCATGGAATGA

948 TACCGTTAAGCTCTGTCTCTTCCAGAGTGTCA
949 TTCAATCAGCCATTTTACGCGTGTTGGATCAA
950 TACCATATCCCGCGGCGAAGATTGTGATGATA
951 ATTTTATCTGAGAGTGATGTTCGCGAACTGATT
952 AGATAGCTCAGTGTGGCAAGCACAACGGTCTA
953 TGCGGCACACTCGGTGTATATCGCTGCATATC
954 CTAGTGATGTTGGCTACCGAGCGCCTAGAGCT
955 ATCGGGGCCACTATCGCCCTGGGGAAGTTATT
956 ACTGCGAGCTTTCGAAAGTGTGTTTTCAGACT
957 GGAACAGCATCATGATGTCGGCTGCCACTTGA
958 GATGTGAGCGGAAATGTAATAGCCCATCAAAT
959 CCGGAGCCGTCCAGCGAGGCCAGATTTAGACG
960 CCCCACCCCTCTATGGGCTAGCAGTGGGCTAG
961 CTGTTGGGAGGTGGCTTTGTCTGCCACTTCAGT
962 CTCGAAGTTTTTTAAGTGCTTTGGTTTTTGCCC
963 TATCTACTCCACTCAATCCGAATGCTTAACAA
964 GGGATGACACCTGCGTGACCTGCGCAGCTATC
965 ACAATATCAACGAGTGCATAGCGGAAGTGTCA
966 GCCCCGGCAGCAGCCATCCCCAGCGCTGCCAC
967 ACAGCCCCGCGACAGGGTCAGGGGGCGGTGTTT
968 TGAGTAGCAGCTTCGGTGGCGGGGCTAGCTCT
969 TTTCGTTGTCAATCAGCTCAACAAAAACGTCC
970 CACAATCAGCCGAGCACATGAAAGAAGTTATT
971 TTTGTGATATCTTTGAAGATGAAATTGTTAAG
972 AAAACAGCTACACCATTATTTGATTACATTGA
973 GGATATAGAGCATTCCATATATGCCGTTTACT
974 GGTCTGAGCATGCTCGGCCATGATCAACGCGT
975 ATATTCAAACTCATTTTTACCTTTTGATTCTG
976 TATATGCTCGTTACTGGCTTAATGTTTTCGGC
977 ATAATTAAAGACCTCAAGCGCTCATTCGAGCT
978 GTGCATGGATGCGACAACAGAGAACGCAATAAG
979 CGCCGCCGCCGGGTCGCGAACTTGGTTCTGTT
980 CTTCATGGGCTTCTAGAAACGTTTTAGGTACAG
981 TAAATGCAGACTAATGATACGGGGCCTGCAAA
982 CGCGAGAGTAGAATAATACCGCATCAGTCTCA
983 GTATTAAAGAATTTCACAACCTCATCCGGGGT
984 ATTGATGGGCATCACCGGTATCAGGGCGCATT
985 AATATCAAGAGTTTATGGCAGCGAAAGCCAGC
986 GGGGATCAGGTCATAAGGTATTTCACCTCTAC
987 AAAAATACAAGGCTAAAGAAGTTGCCCAGTCT
988 AAAGCGAGGTTTAATATCCCGCCCTAACTCTT
989 ATTTTAAATGGCAAACGCTTATTGCCGCCGCTG
990 ATGGCCGAGCATAATGTTGATTATTATATGCT
991 GAGGCAGAGTTCTGCGCAGTCAGGCCGGTATA
992 GTGCCGATTGTTTGTCATTTCGTGCTATTCAT
993 GAAACGAGAGGGTAGATATGAGAGATATTGACG
994 CAGTAGTCGGCGTAACTGTGATGCTAGTTACT
995 TACAATAAAAAGAGAGGCGTTAACTAAGCGTGT
996 CTCACGTCTTCTTCCGTCGCGGTCAGCGCTCA
997 GCAATCTCCCGAATTGCATCTGATGCCGGTCA
998 ACCATCCATCATGCGTAACTTGATGCGCCCAT
999 ACATTGATTCTGAGGATGTGGTACAATTTGAC
1000 ATGTCTCACGACCGGCGTTAATATCCCATTGT
1001 GTTAACGCCTTTCAAGTTGTTTTCACTGCGCT
1002 CAGTTGCTGTTTCTCGCCGTTGGTCATGTTCG
1003 TTAGTGATGACCTTGGTGACTATTTTCAGTGT

1004 AACTTCCCTTCGCGCAGCCCGCACCAGTAGTG
1005 ATTTAAAGGTACGCCGGGGCCTTGGATTTTAC
1006 CGTAACCCTAACTTTCGTGATGGGGAGATCA
1007 TTGGCTTTAACTGTAACGGCGTTAATTGCATC
1008 CTGATTTTACGGATTCAAGAGAAAAAACATAG
1009 TTCTACTTCGACTGGTTTGGACTGGTCTAGCG
1010 ATGTCAGCGTTTCTTTTTTGTATTCCTCTTGT
1011 AATTGTGGCTGGAGTCTGATGCACACGTTCAA
1012 GTTTTTGGTTGCTACGTTTGCGCCCCCCTTGAT
1013 CTCAATACTTGCTCACTGATTGCCGAGGCTTT
1014 TTCTTTCCTGGTTAGTTTTCTCAATATGCTGA
1015 GAAAGCTTCATTCCGAAGATATCAATGCTTCTG
1016 CGGCGAGTCTGCGTCTTGCGACCACTGAGAA
1017 GTATCTCATTATGGCCATGCATACATAATCAG
1018 ATGCTGTATCCTTTCGCCGTATGTGGCTGAAA
1019 TGGCCCTGGCTGTCGAGCGGCGTCCAGATCAA
1020 GCGTAGGGATTCCCGAAGTAGCTAAGGCTTGT
1021 AAACTGCTACTGGCTTTCGTTCGTCGTTTTCT
1022 GGGCAAGGACAGCATGTTAAGCAAGTTCTGCC
1023 TACTGGTTCCCATGCAACAAGATGCAATCCAT
1024 GTGGATACACAATATTAAACCCTAAATATGCA
1025 CACTTTTACATTTGTAATTTAATTATTTTATC
1026 AATTTATTAATGTACCTCATATCACTACAATT
1027 GAATTACGAGAAAGCGTTAACTCACGTCTTGA
1028 TTATTAGCTATTGTATCTGGAAGAATATACATG
1029 TATTCTCAGATTCATATAACCTTAAATACCTA
1030 GTAGAATACGTCGCCAGGATAGGCTTCACGAC
1031 AGTAGAGCCTTGCTTTTGTTGTTTCCGCTAGG
1032 GACCAGATATAACTGTGAAGGCAATCATTGCT
1033 GAATACCTGAATTATTGTTTGCTATTGTTTCA
1034 ATTGCATCAACAGGTAGCGCTGTGCCGGGTCA
1035 CTGTGCTTATGGAATGGACGGCGGGTGCTGCT
1036 AACCGGCCTGCCCTTGACGCCTCGTTCACGCT
1037 AACGACAGAGTAAGCCCCTGAGCGACTTACAA
1038 CAAGAATTTAGCCTCACGTTAACGCTTCAGCA
1039 GACCGGATATCTGATTACCATTCTTACCCACT
1040 CCGACAGTTGTCAGTTATTTACTCTGGGGATT
1041 TTGGGTTGCCGGTTCAGTGCCGGACTCCTTGC
1042 ACTCTGTTCAATAAGTTGGCGTCGCTTTTCAA
1043 GATTTACGCAAGGGTCTTTAGCTAAAGCTGTA
1044 TGATCAGTGGCTGGTTATCGATATTTAATTGAG
1045 GGTTACCGCGCTAAACATGCTACGCCGTGACT
1046 ACACTTAGCCACGTAGCAATGCAGCATGGCGT
1047 CGTATACCTGTCTTTTTCCCACAACGCCGCAA
1048 AATCCGCTCGGCAGTCCGTTGCGCTGTAAGTG
1049 AGAGCGGATTATATTTGCTGATCACTACGCCG
1050 GCTAATGAACCGGTCAATACCTTGTTTGCCGT
1051 GAAAATGGCATGAGCAATAAACAAATTACGCT
1052 AGTGTTTTTCATAGATCATTCTACCATTTCTT
1053 TGAAACCACACAGCGCGTGGGCTTGCGTGCAT
1054 TTTCTCAGTGATGCCGTTTTCCATCCGTTCCC
1055 CTGCCCATCAGGGGAGAGGGCCGGCGCTATCT
1056 ATGAGCAGTGGCCGGTGTTGCCTGCGTGACGT
1057 GAATGCAGTCAGAAAGTAAGTTTGCTGGTATC
1058 AGCCAGATTAGAGCGGTATGCGTCAGCATATT
1059 CAACGCCGGCAGTTTTGGTCGCGAAGGCAATC

1060 CGCAAGATTCAGCAAGTCATTTATCATGTACA
1061 TATGAGTATGTACGCTCTGGCGGACGTGGTTG
1062 ATCAATGGTGAATTCTGGACTGGCGGTGAACT
1063 TTGTCAGCGCGTGAGGATAATCCTCCTTTGCT
1064 GAGATATGGCCTTGCTCGTTTGGTGTTAATCA
1065 CTTAGCCGCCATTCTGAATTGATGAGGGGTCA
1066 CTTTATGACGAACAGCACCAGCCTGGCTATCT
1067 TCCAAGTGACTGAATCAGAATTTAGCGCCTCA
1068 ATTTTGCTTCTCAGCATCAGCTGGAGTTTTAG
1069 CGTCAGTGACTTTTATGTCCATTGCTAATCAG
1070 TTTTTAAACTTCCTCGCCTCATGTATCACACT
1071 AACTATGATTGATAAAGATGGGAACATGTCGT
1072 TCAGTCATGATTTACCACCATTGCACGCAGTG
1073 GCGATGTTCTTGAGTTTGATGCGCTGCCGGAT
1074 GCATTGCCGTTGAAGAGATCCGTCGTTGGCTT
1075 ACTTTAATCAATCCATCGCGCATAGTCTGACC
1076 GTGCAGCTCCGCGCCGTTGCTCAACACAATAG
1077 TATTGATGTAAGTGCGGCACCGGATATCGAGT
1078 TGGACGTTATCGAAGCTAAAGAATTTGCTGGC
1079 TAGCAATGTTCAAACAGGGTTTAATGCCTTTC
1080 ACTGAAACCTGCAATAAATAACTCTTCGAGTT
1081 TCACAAGCGACTTTTTCAGTCGCTGACTTTTC
1082 AGAGGAGGCGGGAGAGCAGGATGATGGGGAAT
1083 GTGCCGCGATGATGGCCGTAGGAGCTATGACT
1084 GGTAGCCTTTTGTAGTGACGATTGACCTTGTC
1085 AAGAACCCACCGGTGAAAGACCGCATTAACTC
1086 CCTTGATTGCATACGCAATGGGTGAGCTGGAC
1087 TACTTTCACTCAAATGGAAGGATGTGGATCTT
1088 TTGTGTTGCCGCCACGATACTCGTTTGAGTGT
1089 TTTTTCAGTTTCAAGAATCCACAAACCGACAC
1090 GGACAATCACAGTACCCCCGCGCGCCTGGCTTG
1091 GGTTAAGCCCGCTACTCGCAGATGTGCGTATA
1092 CACGGGCCGTGTAAAATCATTCTGCTGCTTGT
1093 TCGCAGTATCCTTCTGCGTCCAAAAAGTATCT
1094 GCCAGAACAGCCATCATCTGTCAGTGTCCTTG
1095 AATTTGGACGCAGACACGATGATCCGTATCAT
1096 TTATAATACAAACGGAATTGGGGCCAGCATTC
1097 TTAATAAACGTGATTAGCTCCGCATCCTCCTTG
1098 ATCTTTGACCCTTATCTCATGCCCGAGAAAGT
1099 AACGGCTTTATGAAAATGGATACGTGTACGCA
1100 AACGGTAAGTAACAGCGATTAAGCCGCCTCCG
1101 CAGCCCGGCATGTCAGCCGCTGCTGCCCTAAT
1102 CCATGGCGGTGGCTGGGGCGCGTAAGAATGGG
1103 GTGACCGCATGAATTATGTCAATCAATGCTCA
1104 CGAATTGAAAGTTGACGTGAAAGTGACCGTGA
1105 AGGCTGAAGTGATTTATCACGTTGATGAAGAC
1106 TTTGGTGGTGATTCGATAATGCCGAATTTAAC
1107 GTGGCGAGCTGGTTCGCGAGGCTTATCAATCT
1108 CCCATTCCGCATTGTGATAGGTTCTGCGTGAC
1109 CTGATTTGTTTACCGATAAGGACGTAGAAAAA
1110 TGATGGCTGGCTTACTGCATCCTGAACGATCT
1111 ATGCAAAGGTGGTGATTTCGCCGCCAGCCTGA
1112 ATATTGCCGGGCCACTGCCATCAGGTTTTTGA
1113 ATTTGGTTTGCTGATGCACCCTATTCAGGCAGG
1114 CATGTACTGCACTGATGCTTTAATCTCATTTG
1115 GTATTGGCACTGGTTGCGTCTGCGGCGTTGTT

1116 TCAAAAGTGGTTATCGGTATTGCCGGACCATCGG
1117 TTAAGTCATGATTGTCAAGTCAGGCGATAAAT
1118 TGCAGGAAGCAGCTGGCACTCAAAAATACGGG
1119 TCGTACAACCTGACCGGCATCTGAAGCAATGC
1120 ATGATGATGAGGGTGATCAGGTTTTTATCTGG
1121 GAAATACTTGATGAATTTACCGATACCGAATT
1122 ATTCAAAGTCAGTTTATGTTTATAGATAATTT
1123 TCACCTGTATCTGGTAATAATCCCTTAGTGTT
1124 TTGTCATTGTGACAACCTTCTGTGCCTTACTT
1125 TGATAGTTTTATTCCAACCTCATCCATGATTA
1126 ATGAGTGCGATACAGGCTCGCCATCTGAGATA
1127 TCTTACGTTTACGTTGGCGTGGCTCTTCGTCT
1128 TCAATCTCAGAGAACGGAAGTAGATTGGCTGT
1129 ATGCGTCAATTACGGAAACCACTGGCGTAAAC
1130 GTTATAGGCTTCATTGTCACCTCTGTAGTTAA
1131 CCATGCGCATAGCCTCAACCGCCAGCTCTTTT
1132 TGATGCGCATGGATTTGACGTCTTCAGCGGTC
1133 GGTGACTATTCAATCGGCACGTCAGCAAACAT
1134 CAGTCAAAACCACCGGCAATTTTATGTCATCT
1135 ACTGGCCCAGGAACGAGCTGAACAGGCCGCCA
1136 ATCAACTGTATATGTGCCTGTTTGTCGATACC
1137 TTCTGCTGTCATGGCTCACTCCTCCTCTACCG
1138 GTAATTACCGTTAGCGCCACCTGTTTTAATGA
1139 ACCTTTCTTCGAACTCTTTTCTATATTCAGTT
1140 ATCAGACGGTCAATGTGATATCGCTCATCTGC
1141 CGACTGCGAAGAATGCGCCCGGCACGCCATTT
1142 CCAGTAATCCAGTAGCTGGCTATCATCGTCAT
1143 TATGACGTAATGCATGTGCTGCCTGACCATCG
1144 TTACCGCTCTCACCACCAAGCCTGAATCCAACG
1145 GTGAGGCCAGAGAATCACTTCCCTACTTTGTG
1146 GATAGCCTTGAAGGCGGGAGTGCATCAGTGGT
1147 TCCGGTCATCGCAATTAACTAGCCCACTTAGT
1148 ACGTGGCATCAGGAGGGTAGCGCTTAATTGTT
1149 AGCTCTCGTCTCCCTGGCCGTCCAGCGTGTAA
1150 ATCCAGTGTCACTGCTGGGAAGGTTTCAAGTT
1151 GTTCAATTATCGAGCTATTGGACGGCGAGATC
1152 AACTGAGTGTATCGCTGCGATGAAAAGTCATA
1153 AGAACCGCTGCTAATGGTCTTGCTGCTGCGTA
1154 CAAACAACATTAAATAATGCTAATAATTATAC
1155 CTTGGCGTCACAGTAGGTCAACTTCGCGCAAT
1156 GTTGGGCACATACATCTATGTCTGGTCAGATT
1157 TACTCACCTGATGCCGTAACTTTACCCATCAC
1158 AGCACTAGATTATATTTCGTTGTGGATTACGT
1159 ATCTACATATTGAGCAAATTAAAACTCAGGCGGGG
1160 TATCTGATCGTGGTCGCCTCGCTGAACGTTGA
1161 TTGCCCTGCTCCATGGCACGTTTAATGTGGGC
1162 TGACCACACCATGAAGTTATAGTTATCATGCG
1163 GTTGTTGCATTACGTGAGCCGCCAAAACTATG
1164 TGGTGGAGTGTCGTCTTCTGGCTCCTCAGCTGG
1165 CATGATAACCATCATTACTCTCGCCAATCTTG
1166 GCGACGCCGACCTGCGCGGTGCCAACCTGAGC
1167 GCGGTTAAATATCCCCCTGCATTGGTGATGAA
1168 GAGGCTTTTTATTAAGGATTGAATATGGAAT
1169 GTTTATTTTTTAAGTTATCTGGTAGCACTGTT
1170 TGAGATATAATTATTTCAGCTGAAGAGGTGAG
1171 TCTGTTCTGAATACAGCTAATAATGCAGAAAT

1172 GATGTGCTAGCCGCAGCGGACGCCGGCTGCAT
1173 GCCCGGCATATGTAACCGCTTCAGTGGGTGCA
1174 AAACAGATAATAACTTATCTGAAATAGCAGCA
1175 CTGAAGGTGCAATGAATGGCTCGCATACGTCA
1176 TCAAAAACACGGGGGCGAGGCAACATATTACC
1177 TGCACCAGATGCATTGCAAACGAGTGTCTAAA
1178 TGTTGGGGCTTGACCACGCCAGCCGTGACCAC
1179 ATAAGCGTAATTCGCGGTATGCCTGGCGGGAA
1180 GCTGGAAAACTGGTACTTAGTCGATAGTGACG
1181 ATATGAGACGCTAGCAATCTAAATCCACTAAA
1182 GATACTACGATTGACAGCACTGGAACAGCC
1183 TAATGGTGAGTCGTGGTTCGGCAACTCGCGCA
1184 TTGTGAGTCAGTACCTAATTTGGGATCGTTAG
1185 CAACTCAGGCCATTAACGAGATAAAGGCTCAG
1186 ATAATTCAACTCAAAGTAATAGTTTGTCAACA
1187 CAATCGCTACACGAGTCTTATCCGCCGCAATTG
1188 GAAATGATGTTAACAAAAGACCATTTGACAAC
1189 CCATGGCCGCGCTGCACATCGGCAACCAGCTA
1190 GATGCATCTCAATGATATTGAATCCGCGCGCT
1191 AACTCCAATCCATGTCTGGCTCTTGGAATATC
1192 TTTGCATTGCCGATCAACTCTCTGGCGTCACT
1193 GTCCGCATTGCCGAGCCTCACCATGACGGCAC
1194 TTTTCAATTCCGGGAACTTTAACACTTGCGCC
1195 TAGCCACTTTAACACGTGGTGCGTTGGTAATT
1196 CTGAAGAGAAAATATTCTATGTCGCTGATTTG
1197 GTTATTATCGGATGAATATTATTGTTAATAAA
1198 CTCTACTGCATGATGTTTTTACAGAAGCTTAT
1199 CCGTGGACATTGCAAGCACTGCGGCTGTGCTT
1200 GAAGTGCTGGCCAAGACCACCCAGATATTCGA
1201 GAAAAGTATCGCTATGCCATCAACAATTTTAA
1202 TTGAGTTATTTAAAAGATCTTATGAATTTACA
1203 AAGTCCAGGAATTAAAAGCCGAGGTAGAAGAT
1204 AACAATCAGGTTTACGTGATTGAACTCCGCAA
1205 CTGGCGTGATAGATGGATTGATTGATATCAAT
1206 TGGCTGAAATTGCCGGGGAATATCTTCGTAAA
1207 GAGATGAGCGGTCATAGTGCCGATTACGCTGA
1208 GCCATGATTACCCCGTAATGATCGCATAGTCT
1209 CGTAGCCAGATTTAAAGATTGGAATCGGGTCGC
1210 ACATCATTTCGTGAGATATATTTATCTAATTT
1211 TAAAGCAAAATTGCATCTTTTGTGCACTCATC
1212 GCAATCGCAAATTCAGGATTGCGCTCTGGATA
1213 ATTACGTGGTGTATCCGCTCGCGATCATGAAA
1214 GTGAGGGTACTCGAGCGCTGCTGGCAGGCCAA
1215 AACACATTAAAGGCTCGGCTGATGATTTTATT
1216 GGTATTACGTGAACAGATAGGCCGAAGTTCGAG
1217 AATAACCGATGTCTATTTCTCGCTCGACACTGG
1218 TTTCGGCATCACCGACTGACACCGTTGAATAC
1219 ATGAGAACCAAGGGAGCTTCTCACGGGTTCCC
1220 TGACTACCAAATAAAAGAATCAAACCTCTAGT
1221 ATTGTGCCGCCGACAGCGCAGTGAGGCTCTCA
1222 GTTATCTGTGATTGATTTATATGATGTTAAAT
1223 TCTCGTTGCATCATCGGCGGCCTCAATACCAA
1224 ACCGGCCTGACCGCGCAGAANTCCGCCGCTCA
1225 TCAGTTGTAACTAACGCAGATTTCAGCATTCC
1226 CTTATGAATTTACAGGGTTATCTTTAGAGGGT
1227 CCCATTTTTTATTCTTCCAGTTATCCGGCCCGTCCCGT

1228 CTAAATATTCCTCCATGCCTAGCCGCGTGACT
1229 TACCATCATCTGCTTCGGATGTCACTGTATTA
1230 ATTAATATTCCCCCAACGGCATATGTAAATCA
1231 GGTTTCATTTTATGGATCGCTCAATCGGCATA
1232 TTACTGAACTTCGTTGTTTCAGAACGAGGAGC
1233 GAGCTATTTGTTGACTGCTCTAATATTCAATC
1234 GAACGCAATCAGGTCAGTAGCGCGATGTTGTA
1235 TCAAGGCGACTTACGGCTATGTGACAGCTCCG
1236 GCCCAACTCATCGCAGCCGCACCTGAGTTGCT
1237 ATTGTTCGCGCGCAAATTCCCTATCCTTTTCA
1238 GCTACTCCTTAAATTAATATTTGACTACACAA
1239 TGGTGGTAAATCATGACTGATATTGTGATGTT
1240 GTTTGAAGATTCGTAGCTGCGTGTACTTGTTT
1241 GACCTAGCTTAGTAGAATAAACAAACATATCT
1242 AACGCCGCGATGGTTGATCCAGTTCAGGCATT
1243 TTATCAGTCATTTTCACTCCCCCTCCACTAGT
1244 CAAGATAACCCTACTTCATTATTACCAAACGC
1245 ATTGTTGATTCAATTCAATGATGGCGGTAAGT
1246 GAAGACGCAAGAATTCATGATGGGGATGTGCT
1247 ATTGAACTCTCCGGAAATACCGGAAGGTTGGA
1248 TTGCTGCTCGTATTGAGTTGCCGCTACGTTTC
1249 GGTACAGCATTTTTCACCGGCGGCGCATCATT
1250 TTCCGGGAACTTTAACGCTTGCGCCAGTTGTT
1251 TAATGTTATTGATATTAATACTTGGATTAATT
1252 ATTCGCATCTTGGCATCCGGTGCCATCTTGGA
1253 TCAGGAGGCAGCGTGAAACCAACCGCATGGCA
1254 TATGACGAAAAAATCAGCCCGCTGATGAAGCAA
1255 CTCACAGCCTCAGCAATGAGGCTTTTTATTAA
1256 AAACAGCAAATACACGCCCAGGCCAACTGGCA
1257 GAGTTCGTGGCGCGTCGGTTCGGCAAGCAGAT
1258 GCGGTAAGCGTTTTCTATCAGTTGGCGGTTGC
1259 TTTTCACTGGCGGAGCATCATTAGCAGCTATG
1260 TCTAATGCAACTTGTCTAATCTCAGTAATAGA
1261 TTACCGGTAGTCGGGTCACTCTCATTGGCAAA
1262 TCACTTAGTGCTTTCCAGTCGCTATAGCTCTT
1263 GATCACAGGTGGCTCGCAAATTGATGGAGCAA
1264 AGCATATTAACCCGACGATGGTATTACGCTAT
1265 TACGCTCAACGCCGCGCAGTGGGGCGCGCTCA
1266 TACGCATATTTACTTCCTCGTCTCTTGAGACA
1267 TAACCACCCTCCTTGTTCCACTAGCTCTATCT
1268 TTTTCGTGCGACCGTCGATCGCGTTGACGATG
1269 AGTTGGAAGAAGTCACCATTCTTCCCGAACTT
1270 ACCTATCTACCGGCGTTACTGCTCATCATTCT
1271 AATTAGCAATAGATTTAAATATGTTTAAAGAT
1272 TGCTAAATACACCCCATTTTTGTAATCCGTTTG
1273 TAAAATGGCTGGTTGAATACTTTGTGGCAACC
1274 ACATACAGAGTTAATGGAGCGTAGCTGTTGTT
1275 GCCATAATCCAGACGAACCCATAATGTAGGTT
1276 CAGCATTACCCTCTGCATTTAACAGTGGGTTA
1277 ATGCTTTACCTCATCTGAGTGGTCTTCTTTGC
1278 GCTAATTCTTCATCTCTTAGTCTCCCAGCATT
1279 TTCTATAGAAGATGCAAATGTAACTGATGACA
1280 CTGTTTCTTTCTTTCGTCTCCGACCCTCAACC
1281 CATTACAGAGCCAGTAACCTCGTCTCTAATTC
1282 CCAGTATTTGAACTGTCATGTACGTTTGAGCA
1283 TCAGCCAGCGATAATATGCGGGTACTTCTAAT

1284 CGCCCCGATTTAGAGAATCAAGAACCATTAGA
1285 AGTGGGGAACCTACCGGATGGAATCCGTTTCG
1286 TTAATGGATGTCGGAAATATGAACCCTAATAA
1287 GCATCGATCATTACAGCCTCCACAATCCTCTT
1288 ACCTACCGTCGGGTTATAACTCAGTAAACTTT
1289 CGAAGCAGAAGTACGCCATGGGTTTCTACGCA
1290 TGTTGACGACTTACTAAAGTATACTATTAATT
1291 GTTTTATTGATAAGCCCGAGAAAGAAGTTGCA
1292 AGCACGGATTCGCGATGCGCTGCTTCTTCACG
1293 GAAGCCGAACAGGAACGCATTCGCATCGAAGA
1294 GACGGCACTGTCTCCGGGTTCGAGATTGCCCA
1295 GGTTGCCTGATGCTGATGAGCTTTACGACATG
1296 CAGCGGCGCTTATATAAGGTACATAGCATGAC
1297 ATAGAAAAATCTGCTTTTATTTCCTCCATATCT
1298 GCGGACGTGGTTGGGCGCATACGTCTATGTCT
1299 AATTGAGAGATTGCAGTGCTTGCAGCGGTAGT
1300 GTTAAGCGCCGCATAACAGCTAAAGAGGAATC
1301 TATTCATCGCCAATGGCGTCATGTTCGGCGTT
1302 TGGGGGGGTGATTGCTGCGGGTTTATTGCCTA
1303 CAAGCGACTTACGGCTATGTGACAGCTCAG
1304 TCCTGAGACGGCGCGGTATACGTTCCCGTCT
1305 TACAGAGGGTGGAGAGCTGATCCCCTCAGCAT
1306 CAGGGTTCCCGCGATGTGTGCGAGCGAATGGG
1307 TGCAGCGGATGAGGTTTCGGTTCCGCAATGTA
1308 ACAGCCAAGCTAAGGCCAGCGAAGCGGCCACA
1309 GTGTGACGGCGACATGCTTATTTGCTTGGAAT
1310 GATAAGAAAGCGGCGCTTGATGCTGACTCCGT
1311 CTGGAATTATATGAAGTTGATTTAAGCGCTAT
1312 TCAACTAAAAAGGGGCTTTCGCCCCTTCATTA
1313 GTTTGGTGCTTGTCGTTGGCGTTTTTGATGTT
1314 TTAAGTATCTATTATCAGCAATTGTGATGCTC
1315 TTGAATCCATCCCCTCCTTGGCAATCATGTTT
1316 CTCGTTTCCGCTGAAGGCGGGGGATCGGGGCT
1317 TCCTCGTCGCTGGTCACGCTGCGCCGGCTACT
1318 TCAGTGACTTAGGCATGATGCAATAGCCAGCA
1319 GTATTGGGAAAGTAAATCCGATTGTGATTCTT
1320 TAACTCTTCAACGCTGTAGCTGCGGCGCTCGT
1321 CAGTAAGGGCGCGTGACGAGCGCGGAATGCTC
1322 ATAAACAGCCGTTGGCGCAGATCAGGGAAGTA
1323 TTTCGTGAATGGCTGCACAGGTTGAACAAAAA
1324 TGTGCCGCTGAATGGGTGCCACATGAAACGCA
1325 GATGTTTGTATGTATCTCCTTCGCGTTCTAGC
1326 GGATGTAACCAATGAAGCAGCTCAGCTCGTTT
1327 GTAAATTTCATAACTGACCTCTGTTAGTTACT
1328 ATCACCAACTTTAAACATGTGAAATTTTAGAG
1329 TTTTGTTTCATTTTCATATATAGAAAGCCACT
1330 CCAATGGTGATTACATTCTTGAATCAACAGAG
1331 TTGAAGAGGCTCGATATCTTCGAGAATATTATT
1332 TTAGAGAAGCTACAAGCACTAGTAAGCGGTAT
1333 TCGGTGTGGGCGGTTCATTCGATCTGGGTTAT
1334 CTGCCCTGACTTAACAACGCTTTTCCTATCCT
1335 TGGAAAGCAGGGAACGCGCACCGGGCGTGAGT
1336 TAGCAGTATGGATTTATTACGTAACATCATTT
1337 TTAAATTCTGGAATGCATGCTCAATTATTTGAC
1338 GCCATGCTGATAGCGCAACGCCTCTGTATATT
1339 ATACAAGTGCGTCAAATTGACCTTGAGTCATCG

1340 CGGTGCCTTGTTCCTGTTCGTGATTGGGGCAG
1341 CTACGGATATGTTACCATAATTGCCACATGAT
1342 ATCCTCTGGTCAGATGGGTGATTTCGTGCTTA
1343 TCATTGGAGGAGCAGGAGCCTTCGGCGGTGGA
1344 TATCCAGCCAATGAGATTGCCAATTCTTATAT
1345 TCGTCAAGTCGAAAGATGGCGAGAAGGTATTA
1346 GAAACCAAAACGATTAAGTTCGCGGTGGGCAT
1347 CTGATGGCAATTCAGAATTTCGCCACACTCAT
1348 GCTATTCGCCAAGCCGATTTCGCGGTAGAGGG
1349 AAACAGATTGCGTGGTAGCAGTTGGCCATCCAT
1350 GCGAGCTGGTAACAGTTAAAGAGTCCGCTAGC
1351 TTCCGAGCGCATTGGATGACACTACGGGGGGC
1352 TGCAACGGGCGTGCGGGGTCGATACTGAGCGA
1353 AATGCTGGTATTGATTCGTTAGCCATTACG
1354 ATGAAATTTTTGAACGATGTTGTCCCTCCAAC
1355 CTGATAGCGATTAAGCGCGGTGATAAGTGGGA
1356 GCATTGGCTAATGCTAATCTTGCAAATATGTCG
1357 TTGACTGTTTCCTTGCCGCCTACCGTTTTATT
1358 TTGCATTAAATGGCACGGATGGCGCGGCGCTT
1359 TATCAAACACAGCAGCAAGCGCTGGATGATCT
1360 GCCATAGCAAACAGTAAGGGATGGACGTTCGA
1361 TGAGTAACATTACAAATGTAGCTATTGAAGAG
1362 CGGGGGTGGCTTGTTGGCCTCCCCGCTTCACT
1363 ACGAGATTTTATCAAATCATGCGGCACACTGT
1364 GAGGGCGACCATGCCGAGGACTTGCTCGATAT
1365 TTCATAGCTTGTTGTTTGATTATTACGTATTT
1366 GCATAAAGCAACTCAGTCATCGCATTGGTTTT
1367 GTCAGGTAGTCCTGATGCGTCTTCTTCACAGT
1368 TGCATTCACGGCCTGATTGTGTTGGTTACTGT
1369 GCGAACACCGGCGATGAAGAGCTGGCCGTCGA
1370 ATGCTGCTAACGGATTACCGGATAAAAAGGAA
1371 TCTGCTCGCGCACCGGGGGTCGGTGTTCTTAA
1372 TTACTGGCCTTTGATGCTGATGGTGTAGTGCT
1373 TTGCCAGACGTTGTGGTTTCCAAATTGAAGGG
1374 GCGGGCTTTATATCCTCAAACACCTGCAACGT
1375 ACGATAAACCCAATCATGGTACAATCTACGCA
1376 ACGCTTACGCTTGAACCTCGAAGACTCAGTTA
1377 TGCCTGTTTCTTAACCAGAACATGATGGCCGT
1378 ATTTCCGTTCAGCGGTCAAAAGTCTTTGGACT
1379 ATGGATATAATGGCGTTAGACAATTTCTTTGA
1380 TACTACCTCCTTTGGCAGTCGGATAACTCTCT
1381 GCCTTCGCAACAAAGGTCGAGGCCATTGCCTA
1382 TTGTCAACGCTACGACATTGAATATACACGGT
1383 GTGATGGCAATGATTCTGGCGGCTTCCCGATC
1384 GTCACAGTCGGTCAACTGCGAGCTATGGCGGC
1385 ATTATCTCGCCTGATGATTTGGAAATTGGCC
1386 ATCCGTAGACAGTGGCGATGCCTGGGCCTCAT
1387 TTGCTGGTTAGTCTCAATTGAATCATTCATAT
1388 GCTAAGCGCTCAACCGTAGCCTATAATTGTGA
1389 AATTACGTATAACGTACGCATTAGCCCTAGAT
1390 ATAGGTGCAAATGCCCATCATGTGGCAATTAT
1391 GATGTTACGATTATAGCGCCGGACGGCACCGA
1392 GTGATAAAGTGATTATTCTGCCCTCAATTCAT
1393 GAAGCTGGCGCGGCTGGTCTTGGCGGTGCTGT
1394 ATCTTCAAATACTGACTCTTCAATCTGCTCCT
1395 TCAGGCTTTGGCTTTCCGTCCAATTCATCAGT

1396 AAAGATAGACATCAGTTAATCCCACTCTAACC
1397 TTTCTCAATGCACAACTGATTTTGGTCATGTC
1398 GTGCTGCTCCAGTTCGCCCAGTGCCACTTTGA
1399 TTAACTGCCCGGATTATTCTCAGTGTTCGGAT
1400 GCAAAGGCCAAAGAACTCGCCCTTCAAATAGA
1401 TTCATTTTGACGCGCCCAAGGTTGGTTTCCTC
1402 TACAAGTAAACCAACAAACTACGATATTATGC
1403 CTATCCGGCCTTCTTCGGTTAGCAATGGTGTT
1404 ACAGCGGCCAATTCAGGGTGCTTGCGCTTATG
1405 ATTCATCCAGATGATTGGATTGCAGCGGAAGA
1406 AGTGAAGCTTATCTGCCAACGTTGCCACAATT
1407 CGTAGTGCTGTTTAACCGTTTTTTGTGGTCAG
1408 ACAAGTTATCGTGTTGATCCAGAGAACGTGGA
1409 GTTTTTAATTTTCCGTCAGTCTCACCTTTAAT
1410 ATTCAATTCGATTAGTGTTAAGGAACTTGCAG
1411 ATGGTTTCACCTGTATCTGGTAATAATCCCTT
1412 ACCCTCTCCCTTGATTCGAATACCGGCAGTGC
1413 TTGGAGTTAATAAGTTTGATATGTATACGATT
1414 GTTGTTTTTCCATTAATATCGACAACTTTTTT
1415 CCATCTATTTGATAAATTCCAGATTCATTTCT
1416 ATTAAGTAAGCGTGATGTGTACGGCGGATTCT
1417 TGTCATGGACGCAGCGAAGGCGGAGCAAGCAT
1418 CCGTTACGCCGACTACTGGAACTGTTGCAGAA
1419 ACTGGTTTGGTTCTTGCTCGGTCGGCTGAATT
1420 GATATTTCATTTGGTTGTTTATCTAGCTTGTC
1421 TCTTCATGATCGGCAGAGCTGGTTTGTTCGAT
1422 TCAATATACATACAGAAATAGTCCTCGTAAA
1423 GGGTTAGATTATATAATCAGACATTGGGTTTAG
1424 ATTTACGCTGAGATTCTGCGGCGGCGCGGTAA
1425 GTGCTGTTTGTTGATGTCCTGCTCGACGAGGC
1426 AATGCGTTGATGTAAGCCTCTTTCACCGTGGCG
1427 ACCTTATTTAATTGGAATGGGTGGGGCTTCAT
1428 TTTGACCTTGATAGCTCGATAGGCAATCAAGA
1429 GAGCAATTTACATTAGAGCTAAAAGATATTGC
1430 CGACCGAGGGCCAACTATTAGTAGATAGAGTT
1431 TGCTACAGCATTAAGTTCAACACTCTTTTTAG
1432 AAGTCGCAATCATAAAAGATGTTTCGCTAGAA
1433 ATATAGATCGAATGTTAATTAATGATTTACGA
1434 ATCTTGCCACAAGTCTGGTATGTCCTTTGAAG
1435 AAAGGCGGAAAGGCCATACTGAAATATATCCC
1436 CCATATAGCTTTCAATAACTTGCTTAGTCGCA
1437 CGCTGAATCCGTGCAGTTGGTCCCCGCGTTGT
1438 TGCCCGCTGCACCAACGGTTCAAATCCCAAGC
1439 TTGGGGCCGGCGGTATCGGCCCTTGGACTCGT
1440 TAGATCCATCTCGTGCAATGCTTTCGCGACTG
1441 TGCAGAAGCAGCGGCACAAGATGATGCGGATT
1442 TACCAGACGCCAGCGGCCAGCAAGGCCAGCGC
1443 ATGAAATGCGCGCAGAAACTATCTTCAATATA
1444 AGTAACAGCAGCTCGGCATGGTTTAACACCAA
1445 CCTTTGTGAGTGGTAAATAAAAAGCCCCGCGA
1446 CTTCCGCGCCGATGTCGTCCAGATATTCACTT
1447 ACGCAGGGCGGCAGAGGCTGCAATGTTCCATG
1448 TCCCAACTGGCAGCATGGGCGGAAGTGATGCT
1449 CAAAGCGCATCACGGATTTTCAGGGGGATAA
1450 TTCGAGTCAATCATGGGAAGACTATCTTTATT
1451 GCGACCACCCCAACGATGCGGCGGACTATTTC

1452 ACAGTACATCCTACTGGTTGGATTTTTGATTT
1453 AGAATAACCCCCTTGATCCAGCAAGCGTAAAA
1454 TTTTCGGATGATGAGGCGGTTATTAGCACTGA
1455 GGAATACCAGACGTATTATTAGCTATTACACTA
1456 CCTTGGCTAATGGCGGGGGGGGTTGTCGATTTA
1457 TTCCGTAAGTCAGAATCACTAACAGCGAGGTA
1458 AGGACCGTGATCTTATGCTTCTTCCCCATTCT
1459 TACCATCTGACCAGAATTCGGATTGGTTCAAG
1460 GTTAAGTTCTTCTAGCGCCTTATCAATCTTAC
1461 CTTGAGCATCTGCCAGAACATCATATGCATCT
1462 TGCATGCAGAAAGTGCTTGCAATGTCTAAACA
1463 AATCAGCCAGGCGTCGGACGGGTTTTCACAAG
1464 TTCGAATATAACGAGCTATGGAGCGGTGAGCT
1465 ATATATAAACCCTTTAAATCCTGAGCATTATG
1466 ATTGGTGAGACCATCAGCAGGAATGCTACTCA
1467 TCGGTGTTTTCGTTGTAATGCTTCTCGGTATA
1468 AGATGGCCTGATTGACCGCCTGCGTCATATCG
1469 TACCATCATCGGCCTCGGAAGTTACTGTGTTA
1470 TACAGGCGCACACAGGCCGCCGCTAAATCACA
1471 TAACAACGAACGCAAGCGGAAATCCGGCTGGG
1472 ATATCCTCCTGATGCCACATCCGCTATTTTCT
1473 ACTGTACGGACTGGAGGAAGGCTTGGACGTTA
1474 ACCAGTAGCCCTTTAAAGGCATCTTTACCGAT
1475 TCAACAACAGTACCCGACAAAATGCCGGGAAA
1476 GATGACGAGATGGCGCGGCTGGCTAAGTCAGT
1477 AGATGGTCAGTAAATTACCTTTCATTCCGCTA
1478 AGTAAAGCAACTATCACTGATTTCATGATGGA
1479 ACTAAAGGAATGTCTTTACGTGATGTTTCCCA
1480 AATCACCAGATGGCGTTCCGGGTGTATTCTTT
1481 AAAAACATTCTCTATATTCCTAAATTTGGACA
1482 AATCCTCCGCTGGCTTGGCGGCTGTGAATAGT
1483 GATGAGCGAGAAAACTTGCCATCGATAATGTT
1484 CCTTTTTATCCGGCAATCCGTTTAAGGCATTA
1485 ATATCAACGCAGCAATATGATCCGGAATTGCA
1486 TGGCCTGTAGCGCCGGAGTGATGGGTGGGCAGG
1487 TCTTCCCATGCTATCTTCGGACTTACCATTGA
1488 TTTTGTATCACTCAATGAATCTTTCAAAGATG
1489 ACGATTCAGATTTGGGTAAATGGGGGAATAAG
1490 GCGTGATTAGCTAACACGGTAGCTTCTAAATC
1491 CCGGAGTTATGTATATTATTAATATTGCTTGG
1492 GCTCAGATAGATATAGCAATAATTTATCTTAT
1493 TGTGATTCATCCAACGTGTTGGCATGAGATAT
1494 TGAATTAATTACTTTAATGCCTAGGTACTTAA
1495 ACTTTATTTGTTCGTGGTTTCACATTTAAAGA
1496 TGCCGCACTCCATCGTTCGATTGAATTTGCTG
1497 AATGTAACAGGGTAGACTATAATAACTGGCAT
1498 TGCTATTAAAGAAGAAGAAGCTTTTAATCAAG
1499 AACGGTCTATGGAACCAAGTTAAGCAGTATGT
1500 AAACTTTAGACTATCTACGTGTTAGAGTTGTT
1501 CGTGTGGTTTAATATGGAAATAAATGTAGTAT
1502 GTATAAAGAGTATAGAGAAATTATATGTAATA
1503 ATAACAGACGCAGACGAACCTACAGCAGGCCAAC
1504 CTGGCGCTTCTTGGGCTTCTAGAAACGTTTTA
1505 TTTCTCTATATTCAACACTTTGAAATTGATAT
1506 CATTTTTGTAGTTGAATCGTGTGGTTTAATAT
1507 ATCAAGTTGATAGAGTGGAGACTAATCTTCTC

1508 GCGAGTAGCGTTACCCTGACGCTCGATATTCAC
1509 TGGCCCGCTGTCGGTAGCTCTGCATCTATTAT
1510 TGATAGATGGATGCATGCACAAGTTATAGAA
1511 TCTAATATGATAATTAGTGGAAAAAATATTGT
1512 AAGATTAGCATTAGCCAATGCAGTGCCGGACT
1513 GTTTATTGCAAACACGCTAGGTGCCGGTCTGCG
1514 CAGGGCGTAGAGCGGGTCATTCGTTTTCCCTT
1515 GAAATATCTAGTCAGTTTTTAACTGCTGGAAA
1516 TAAACGTATTAATTTAGACTCACAAGCATTTT
1517 ATACGCCATGGATTCCGCTAACTCTTCTGAAT
1518 TAAAAAGTGTGATATGAGTAGTGTTTAAAGCG
1519 TTTTCGTATGTCTCATACTCTTCAGTTCTGAT
1520 GACTTTCTTTGCCACTGAAGCCCAGCGCAATT
1521 GAAACTGCCGACTCAACCCCCCACTGTTCCG
1522 ATGAACCGGTTGAAATAGTTGTTACTGGTAAT
1523 TTCGTTATATTCGAATCCTTCCAGCAACATCT
1524 TGGCTGCTGCTGATAAATCCGCGCCTCGTAAG
1525 TTCATTATCACCGGGGTAGCTGATTTTGGATT
1526 CTGAATACTCTGATAATTTAGGGTCACCGGCGT
1527 CGGCTAACAAGCTGGTAGTCGATTCTGGTTT
1528 AACATTTAATTAGACCATGTTGGGTGGCTGTC
1529 CTACCGCAAGCGGCGGTAGAAGTCCGTCACGA
1530 TAAATTAGTGAGAATTTCTTGGTGGTTGACTT
1531 TGGTACAAGCGGCTTTGCGTTGGCACTTTGTT
1532 ATTTGCGCATCAGCCTGCGCATTCATCACAAC
1533 GCGTGTTGTTTTGTTCCTTTGTTGCACGCTAA
1534 TGGATAGCAAGAAAGAAGTGCATCTGTTTGCCG
1535 AGCAAGGATTATGGCTACAGCGCGAGCATCAT
1536 TTCGCCAGTGCTTGAACCGGATCAACCATCGCG
1537 GCGGCAGTATTGCTGTTCTTCCCGGTGTCAAT
1538 AGTCGGTGTTGCAGACTTGTCTTGATGCTTAC
1539 CTTCGCTGGGAGCACTGTGTTTCCCAGGCAAA
1540 ATAGATACGATGGGACGTTGATTTCACTGGAT
1541 AGGAAATAAATATGCGTAACGCTTATCGAGGT
1542 GAGCCGCTGTGGCGCGGGGCCTTATCTATCGC
1543 CGACTGAGCAAGCAAATTTAAACGCTGCAAAT
1544 GTCAGTGAGTATTGCGAAGCCATCCAAATCAT
1545 TTGCGATCAAGTGTATTGCCATAGTTCAGTAT
1546 ACAGACGGTCATCCCCATTCAGCTCACTGGTA
1547 ATACTGAGTGAGTGCGGGGTCAGCTGTAAGTT
1548 AGAACAGCGGCGCTCAATTTGCTTTTTGTCGA
1549 TGTCTCCCGTCGTGTCGTCTTACTACTGTTGC
1550 ATGAGACGAACTCGAGCGCTTCCCCTGCAGTCT
1551 CATTTCCGTTCGTATCAACTGTAACACGTGGT
1552 TCATGCAGTCAGGTGCAGGCCAGCAATTAACT
1553 GTGTGACGGGCGCATTCTTTGGCGTAGTTCGA
1554 ATTGCATCTTTAGTACATTCGTCGGTGTATTT
1555 ACATGCTGTAATTTCATCGGCTGCAGCGGATG
1556 AAACTCGGCGTTGAAGATGTTAAATCGGCGAT
1557 ATCAATAGCATAGGAATGGTCCCGCTGTATGT
1558 AAGCAATTCGAGCCGGTTCAAAGGTGGTTATC
1559 ATTCACCCGACAAGGTATATCGTCACCATAAA
1560 ACGGTAAGGGGATTAGGCAGGCGGCATCAATT
1561 CATTTACGATAATTACTATGCTATCGGCTCAG
1562 CTGTGAGTTTTGTTCCGCTTCTGGCCCTACGA
1563 AACTTTATCGGAATGCGCAGACCAGCCGGAGC

1564 TTAGTTCCCTGAGACATGATTTCTTTTTGCAT
1565 AAGGGCTATAGATTCAATTGGTCGAAGTTATT
1566 ATTTCGCAGAGGAATTCGTAAATAGTTTCCTT
1567 ATTCATAGCCATTCTCGTAAGCAAAATGTATT
1568 AGTGAGGCTAATTGGCACGCCAAGCCAGTTGA
1569 GCTCTACGATGGAAGCATCGGTGTTACCATTT
1570 ACTATCACGAATACGGCAAAAAGGTTGCCAGC
1571 ATCTTCATTCCAGCGTTAACTTGGTCGATGCTG
1572 GTCATCAGCGCTGGAAATGCAGCCTTCAACTG
1573 CTTACAAAATTCACTATGCCATTTGTCGCAAAG
1574 ATATAAAACTCCCTAACCCGGCCCCGCTGCCG
1575 TTGATTCCGCAACTTGAGCAGCCCTGCTCAAT
1576 AAAGCACTAAGTGAGAAGGCTCGTGCTGACTT
1577 ACGCCACGGCCGTTGATGTCCAGCGTAAACAT
1578 ACTAATTAATCAGAGAGCTAATGAAAGCGTAGT
1579 CATTACCTCATTGAGACTCGGAACTTCATCAA
1580 TATCTTGACTCGCTTCCAGTTTATCCCAATCA
1581 TCTCCTTATAACAAATCGGTATAACGGAATTC
1582 AACGGTAAGCGCTGGAAAGTCGGCAGCGTTGA
1583 ATAGCGCCCGGTCTAAAATTATACCGTCCAAC
1584 TTTCCGTGGCTGCATTACTTGGTTTCTATGTT
1585 GGCGATTGCTGTTTATTGGGGTAACTGCAAGT
1586 ATATCCCCCCAAATTAACCGCCCACTGGGGTG
1587 AATGTGCATACATTTAAGGTCGTCGGTTGTTA
1588 GGTTGCCGCTAATATCAGCGGCGCTTATATAA
1589 CTCTGTGACGGTCACAGCCTTGAAGTTAAGCC
1590 GCTCACTGGGTGGCCAACTGAATAATTCATAT
1591 GCATCAATTATATGGGATTCAAGATATGAAGC
1592 TTCTAACATAGAAGACGATATGCTAGCAACAA
1593 GCTTAAAGATTCGTAGTTGTGTGTATTTGTTC
1594 TAATAATACTGTTGATGCGCGAATGACATCAG
1595 GTGGGCGGTGAAGCTGTAAACCTGCGTTACTT
1596 TATTTGCTCAATGCGAGAAATCTGGGCGGTTT
1597 GTTGCCAGTTGGCTAACTCGCGGCAGAATACC
1598 CAGTTAGAGCCATTACTAAGGCTTTAATTATT
1599 AAAGGCGGATGAACCTTGGCAGTTTCTAAATT
1600 TGGCAATAACAAAGGTTCGTTGTTATTCATCT
1601 CGTATCGAGTCCTTTAATAGCGCTGGCTCCTT
1602 GAGAACCGGATCACTGGCTGGAAATTTAGTAT
1603 TTCAGGGCTTATCCAATGACTGCCGTTTCGCG
1604 AGAATTCAGGGGCGTTCGTCTGTATCGCTCTC
1605 TATAATATCTCTGATGTGTACGCTTCCATTAT
1606 TTTATTCAGTTATATTAATGGGTTCATCTGTC
1607 AAAAACCTACTTACGGGTAGTGGTGTTGTTTT
1608 TTCTAGAGGCATTCAAATCTCTATGTAAAGCA
1609 GGCTTCTAGAAACGTTTTAGGTACAGGTGCAT
1610 TCAGGCTTCCCACCCTCACCGACTCGATACAT
1611 ACTGGAATCTCGCCAAATAATTATATGTATTG
1612 CCATCCATCCGTGGCGCTCATGACTGATTTCA
1613 GAAATACGTTTAGGGAAACCTTGACCGTTACC
1614 TGTTCTGAGTCATTTGATGATTTAGCATCAGT
1615 AGCTAACTCTATTTCTAAAGCTGCACAAGTTAA
1616 CCGGCTCCAACGCTTTCAACTCTGCAAGTTGCT
1617 TAGACAACGCCATTGCGATAGCCATTGCATCCG
1618 ACATTTTTTTAACCAAACATGCTCTTTGCTGT
1619 TTGCTGATAATGATTATGCTATCGCTAAAGTT

1620 ATGTAACGGTGTTACCTGAGTAACTGATAGAT
1621 TGGCAAAATCCCATGCCTTATGCATCCGAATT
1622 ACGGTTGAACTCTCTGGAGATACCGGATGGTT
1623 AGCATCCCCTGCCCGTTTGTCATGACGCTGAC
1624 CTTACTGAACTTACTTAGCTTTCTTCTATTAT
1625 ATCACCCATTCTTTTTCTACATACATTTCATA
1626 TGGGTACTGTACGGGTTCACTGTGTTCAGTCA
1627 GTCGTGAAGCTAAACTCCGTATCTCATGAAAT
1628 AGTACTGGGAAAAACTGGGTGGCACCTGGGGC
1629 CATTTGCAATAAAATATCCGCGCGCAATACCG
1630 ATAACTGTTAGTAGCTGACAAGTAGCTCACAA
1631 ACGCAGCCAGCGCCATCCATTGTTCTTCCGTT
1632 GTCATGATGGGTTAATCACGTCAATGCACGCT
1633 ATTTTAATGTAAGAATTACCGGCAATGGCGAG
1634 GTTATTATCCGTCACAACAGTCTGTTCCTGCT
1635 AGTTGCCGCCCTTATCCCATTTAAACATTCTT
1636 GATGCAGCGCCCAATTACATAGCTTCAATCAA
1637 CGCGTCAAGTTGAATTAGAGATGGATATGCAT
1638 AGGGAGACTACGAGCCGCGCTACACAATGAAC
1639 CCAATAAAAATCTTCAACATACTCGCGTAGCT
1640 ATCTTGATAGAACGCGAGACGGGCTTAAGC
1641 AGATGAAGAATTATTAGATGCTCACTACATTA
1642 TGACGATGCGATTAGTTTATCTACGCCGGAAT
1643 TGACTCGAACGCCGCCGTACCTTCTTCTGCTA
1644 GCCTCTGCCGATTGCTGTTCCCACCAGTCCAG
1645 TTGAACAAGATGTTGTTCGTTTGCGCATTTCC
1646 ACTTTTTGCTAAAGACAGCGGGTAGATTCAGAG
1647 TCAAGTTGAGCCTCGCGGCGCGCAACCTCTCC
1648 CACCCAATTCCATATCATCATCGTCATCATCT
1649 TGTTTTTTCCGCTTATAATCATGTTAGACAAA
1650 GCAAGGGATATTTCAAAATCAGTTCAGTTCCC
1651 AGGTTCGCATGGGAGCGTCACCCTGCGGCAAA
1652 GCTGGGCGTGATAGTGCTAGGCGTAAGATTCA
1653 ATTTCTCTCAGCATAGATTTGCTTCCAATAATG
1654 TATTCGCAGCTTTGCAACCTCTTCCTTGTCTG
1655 CCGTTGTGATGAATGACTGGAGCTAAGGACAG
1656 GTTGAGATGAATCTTTTTGCTCTTGCGGCTCT
1657 GCTACACAGATTAAATTGAGTGACGGCTCGCT
1658 CTTGGGTTGATGTTGGCGGGTGGGTTGATGCG
1659 AGCACGAGTTCCATATTCGTAAAGTGACTCCA
1660 TCAGCAATTCCTGAAGATGCCGGAAGCATTGC
1661 ACATGGGAATAACTAGGCATCTTGGTGCTGAT
1662 ATGAGCAGCGTTATAGCTCAGTATATGGCGAC
1663 TGTGACCGTCTCCCAAGTCTTAACAGAATGGAG
1664 AGTACAAAAACTTTTTTTTTCTGTCAAG
1665 CCGTCTTCTGCATTCACGGCCTGATTGTGTTG
1666 GTTAATGACATTATTTTTCGCAAGATTACACT
1667 ACCAAGTGGTTATCTTTCTCATATCCTTCATT
1668 TAGCATTGACTGAGTTAATTCTGTCTTTCACT
1669 CAAGACTTTCGTATCCAAATAGTGCCGTTGTTG
1670 TCAAGCGCAGGTTGTGGGAGGAGGTTAGTGAT
1671 GGCAATTATCTTAATCCACGCTTCCAGTGCAT
1672 CACTTTTTGTTAATAAGGCATGGATGCTTGTT
1673 AAAGTGACCGTGAGGTGGTGGGTATATCCATT
1674 TTAGTAATAGCGCTATGGAATCTGGCGGAAAT
1675 TGGTGACTGTAGCAAACGCAGAACCAACGATT

1676 GGATTTATCGCGGCGCAGATTGTAGCTATACC
1677 ACTAAATTACCCTATTTAATGACTCCAAGTAAC
1678 ATTTTCTACTGAACTTCGTTGTTTCAGAACGA
1679 TTATCTACGCCAGAATGGTATAAGAAGCAGTAT
1680 TTCTTGCTATACCATGCCCTGCATTCTTCTGA
1681 CCCGTCAGCAAGATGTTGATCACCTCTTTAGT
1682 TATCCGGTGGGGTTATCACGGATGCCATGATC
1683 AAGAGATATTCTCATTCATATTGACAGTGTTG
1684 TAGGCTCTAAAATGTTCAACGCTTTATCCAGA
1685 GATTTAATACGCAAATTCCTGGATATGATTAG
1686 TTAAGCGCGGTTTTTTGGTTCCAACAATATTT
1687 ATGAGGCGCAGACATTATTGGGGCTTGAGGTT
1688 ATGATGCTAACTAAAACCCCAAATTCCGGATT
1689 TAATGTCATTAACTTTACTTGTCCAATTACTT
1690 GCCGTAATCAAGACGAACCCAGATTGTAGGCT
1691 AGTTACTATTGCGAACTTGCCAATCGGGTATA
1692 ATTTAAAAATTATGCCGCAATCATCATTGATGA
1693 CTGATGTAGCTAAAGCGATAGCCCCAGCAAAT
1694 ACAAGCTAGGTCAGGCGATAAACTTTGCCACT
1695 GCTACGGCTACCAAATCTGGCGCAGTGGTTAC
1696 TCGCTCTGCTGTAATGCTGGCGTCCATCTCAT
1697 TCCAACCGCAGGTTCCCCTACGGTTACCTTGT
1698 ATCACCTTTAATCCTAGAAATAACATTAGCAT
1699 TTTGTGCGAGTTCAATTTAGAAGCATATATAC
1700 TTGCGTTGGTTGCTCATCAATTAACTCTCATT
1701 TTGGTTCCGCGTTCACTAACGGCGGCTTTGGT
1702 AGGTGGCGGCGGGTTGCGCCGCCACGGTTCCC
1703 TTTCATGGTCTCCCCCTCCCGGTCAGCGGGTT
1704 AATCACAGACTATGAATAGCCGCCACCTTACC
1705 GCTAACGCAAGCGTCTCCCGCGTCACGAGTGC
1706 CGCATTGCTCGTGGGCGTTGTTTACTATCAAG
1707 CAAAACAAATCATCACAGTGTCGGCGTGGTTG
1708 ACCAGCGGGATAGCATCACTGGACGCCATGCT
1709 AACTGCTAATGTTTCAGACTGAAATATCAACT
1710 ATCAATATGGGTTTACAATTTACTGCCGCACA
1711 GACTTGTTGATTTTCTGCCAATCACTGCGAGA
1712 TCCTCAAACCATTGCTTGGTACTGCCCATTTAG
1713 CTAATGGCTTATAAGGGGTCTGCTCACGAAAC
1714 CCCGCATTATCAACATTGCTGGCCCGTGGCCA
1715 ACGGCGGAAACCAGTTCCAGCGCCACGTTAG
1716 ATCGGAACCCCTCGATGTTACTATCCGACATA
1717 GCACATTGGAAAAGTGCTGGGAGACTTATCCG
1718 AATCTCTCCAGTTCCGTGTTTAGCCTCCCAGC
1719 CGGGTGCATGATGTTCATGCTGTCGAATGCAT
1720 AGCACATGTTATTGGTATGAAAATGGCTTGGT
1721 ATGGCGTGTTTACGCCCAACGGGGTTCAGGAT
1722 CTGCGTAAGTTGATACCGGCTGCGCTACGCGA
1723 ACATTGCTCACAGCCTTGTGTGCATAACTGGT
1724 CATAGATGAAGGTTTTGGCCATGTCCCTTGTA
1725 AATAAAAGCGACCTCCGGAGAAGTCGCTTATT
1726 GGGAAGCTTGATAATCGAATGGCTCTTGACAT
1727 CTCCGTCGGACGAGACTTGGCGATATGAAGGG
1728 ATGCCTACCAGTTGATGGCGTTCTCTATACGA
1729 TATTAATGAGTTAGTTGAGTGGATCGCTGATT
1730 ACTTACAGAAGGGATTAATAAAGTTTTCGGAA
1731 GTTAATCAGCGTGCGCCCGATATCTTTTGAGGA

1732 TGCAAGAATCCCTCAATAATATCTACGCGACTT
1733 ATAAGAAACTTGCACCTGATTGCTGGGTAACT
1734 CTTTTCTGAAGTTGTAGCCAGCCAAGCCGTAA
1735 GTGCAATGCTGCTATTAATGAGTTAGTTGCGT
1736 CTACGTGAAATCTGAGCGAGTGGCAGGGCTAA
1737 AAGGCGCTTAGTGAGAAGGCTCGCGCTGATTT
1738 ATGTGCAACCAATCATTATCAAAGCTGCAAAC
1739 AAAGCCGGTGAGGATTTCTTTGATGTATTACC
1740 CTTCGAAGTCACCGAGCAGACGGTCAATAATT
1741 ATATACCCATAGCCATGGATGGTAGCTGTTCT
1742 AATGTTAAGTTCTCGCTCGCCGAATGCTTCCG
1743 ATACCAATGCTCTTTTTTAATATTAGCGGTCA
1744 GTGGTGTTTGACCGTATTATGGTACCGAGTAT
1745 AAGAAGATACGACCGAAACACCGATGTATGTT
1746 TATGCGACGTAGCGACAGCAAAGGTGGTTGAT
1747 TCATCCCTTTATTCACGGTTTTTCCTCCCCGT
1748 TTCGATTGAGCAAAACAACATTTGCCCGTTGC
1749 TCTTCCCCTCTTCTCTTTTTGTTGGTTCTTGT
1750 GTGAAATCAAAACTTTAACTTTCATTATTACA
1751 AGTGGTTTCTCCGGAGAGTTCAACTGTGTGGT
1752 ATCCATCCCCTCCAGTTGGTCAGCATTGATGG
1753 GAAGAGAAATAAAGACCGTTGAATTTAAACAA
1754 TAATACCTTTTAAGTTATCAACACTTTCAGCGCG
1755 GCAATATCCGATACTGAGCGTTTTACTTTCTT
1756 GGATATGATTAAACAAATCAAGCGCAGACTGT
1757 ATGACAGACAAAAAGATGGCTGAGTTCTTCGA
1758 AGCACGACGCTGATGATATTAAAGTCGTCGAA
1759 GGTGGCGTCGAGTAGCGGGGAATGCTGCCGAT
1760 CTCGGCAGAGGTCAGCGATTGATGGAAGCAAC
1761 ATTCCGCTTTATGATGAGTTGGGATTGTCTTT
1762 GCTGAAATCTGCCGCGCTTACTGGTCATTGCT
1763 ATGATAACGGATAAGGATGTGGTGCCCGCCTT
1764 ACTTACCCCGAATTGATGCCTTAACCCTTGCA
1765 TGGTAGAGATTATCGAAGCCGCTTACTGGTTT
1766 CGCAGTTTTCAAAAGCGAATGAGCAAATCATT
1767 ATCTGCCGCTGGTTCAGGGTGCCACCCTTTAT
1768 CACTACCGCAAATCCGATTTTTGTAAGCCTTT
1769 TCGGCAGGCTTGCCAATTGAATCAACGCTGGT
1770 AAGCAATGGTATCTTCTTTCTTATTGTCCACT
1771 GCGAGAAAACTTACCATCGATAATGTTGGCATT
1772 CTGCTGTATTGCGTACAACTCTAATTTGAAAT
1773 ATTGGATTTGAGCCAGTTCACTTACAGAGTCA
1774 ATTCAAGGGTATAGCGCTGGAAACGTCCGTTA
1775 AGGTGTTGCTATCGGACCTGGTTCTCTAACAAC
1776 ATTGCGGCGCGAAGGACCGGCAATAACTTTAT
1777 ACTGATAGCGTTGATAGACTTAGCGCCGAACT
1778 AGAATGAATTCAATCACTCCCCAGCCAACAAC
1779 ATTTCTTTGAGTAGCGCCGGAATGATTCCCTG
1780 AACGGCTTCCAAGTTTGCATATTTATTTTTAA
1781 ACATTTAACGGTCTTGAAGTTGGCGCATATGA
1782 GGCCAGTGACACGTAAGGCTGGGCGGCATTCAG
1783 TGCATATGACCCATTCATTGCCCCCTCGGAGA
1784 TTATGGTTAAGCTGGTGGCTGATAGCATAGAA
1785 ATAATTAACTGTTGTGATTCATCCAAGCGTGTT
1786 CATTCTTCATTTGTTCGGTTTCCTCTTGCGTT
1787 GTGATTGTTGCTGTGATTGTCCTGATGTTTGT

1788 TTCCGGGCGCAGGTCGCCCGCCGATTGCGTA
1789 AGTCAGCACTACCCATCAATACAGCGCCATTA
1790 GGTGACCGTACCACGCTTTATCTTGTTGATGA
1791 TTTACCAATACCGAATATGCTCAATCATTGGC
1792 TGATCGATGTAACTAATTTTTTCAAAACTTCG
1793 TAACCGGTCATGTTTGCTCTGCGTTAAACTCA
1794 TAGCACACTTGTCCAGTGCAGTTTGATACCCAA
1795 TGTCCATCCCTTACGAACGCTGAGAAGTAACC
1796 TCCAATTCTATTATCAAGCGAACTAAAATATT
1797 TGATTAAGTCAAGATGCTGCTCTATATAGCCA
1798 AGGTTAAGATTTTAAGTAGCAACCTTTATGCC
1799 AAAGAGCGTACGATACCACCGCTCAGCAAGTA
1800 TCTCAAATACGGG
1801 TGTTAGCGAGAAATGTAATTTTATCCCAATGT
1802 ATGTTAGTTCAGATGAAGGCTGATCTAGCGAA
1803 AATTTAGCCACTAGAGGTTTGATTCTTTTATT
1804 AGATAATCAGCCTCGAATATCTGGGTGGTCTT
1805 GCACTGGCGGCGGAAAGTGCTATCCCGACTGT
1806 GCGTTGTTGCTTGTTTTCACCATAATTTATAC
1807 ACAGAGTGCCGCCTTAGCCGCCGGCGCAGCCT
1808 TTCTTTAGGTCGTCCAATTGTGGTGATATGAG
1809 TATTAACAAAACCTGTGCATAAGCATCTATAC
1810 AAACATCAGCGGTCCCGCTGGATGAATATGGT
1811 AAAGGCTGATGAGCCTTGGCAGTTCCTTAATT
1812 AAAGTGGGCTTCCGTGGTGGATTATATGTGAC
1813 AATACATTAAAGGCTCGGCTGATGATTTTATT
1814 AATTAGAAGCCCTAAAATAAATTACCAATCTG
1815 AATTTATTAATGTCCCTCATATCACCACAATT
1816 ACAAGTAAACCAACAAACTACGAGATTATGCG
1817 ACATCAATTATTAATTATAGTGTTATACTTAT
1818 ACTGAAATACAGATAAAATGAATCGTCGAACAT
1819 ACTGCTTTCATTGTCATCTGATTAACCTCTCT
1820 AGAACTATGTGCATCTGTTCTACCCGTCAGAG
1821 AGGAGCAGGGAAAACATTCTCGACTTTAAAGT
1822 ATAATATGATCCCTTTTATACCTCTATTAATG
1823 ATAATTAACTGTTGTGATTCATCCAACGTGTT
1824 ATACATAAAACCAGATAATGCGCCGCCAGACTT
1825 ATATGATTCCTTTTATACCTCTATTAATGGG
1826 ATATGATTCCTTTTATACCGCTATTAATGGGT
1827 ATGCTGACATTCTTGATGGGCCATTCATGATT
1828 ATTATCTCGCCTGATGATTTGGAAATTGGCCG
1829 ATTTACGAGGGCTATTTCTGTATGTATATTGA
1830 CAAATCCTGCGCATTATGTGACGTGTTAAAAT
1831 CACTAAAAGTATTATTTACAACGGTGTTTTTA
1832 CAGAGTTTCTACCAATTCGTTTATTTGCTGTA
1833 CAGCTATTAAAGAAGAAGAAGCATTTAATCAAG
1834 CAGGGGACTGGCGAACAATGTCTTTCATGAT
1835 CCATATACGACTCTATAACTTCTTTAGTTGCC
1836 CCTCGGCTTTCTTGCTGCGGTTCATCTTCAGT
1837 CGCAACAGCGTTTAATATTCCAGATTTTTTGG
1838 CGTTAGGTAAACTCTTTTTAAACTCGCTATAT
1839 CTAATATTGAAGTCAAGCGCTCAGGTGAACGA
1840 CTACCCGTTATTGAATGAATATAGTATCAACA
1841 CTGATAATGATTATGCTATCGCTAAAGTTGGT
1842 CTTGACCGTTACCAGTGACCATTATTTCAATG
1843 GACTCGAATAGTCAATTAGTCGCACGTTTCGA

1844 GACTCGCTGTGACTTTATGTGAGTTGATAGAT
1845 GATCTTATGAATTTACAGGGTTATCTTTAGAG
1846 GATTCATATAGAATGTGGTTTCGCCTGTGTCT
1847 GATTCATATTTCCAACATCCATTAAATAATCT
1848 GATTCATATTTCCGACATCCATTAAATAATCA
1849 GCGGACGAGCCTACAGCGGGCCAACTATTAGT
1850 GTATATAAATTGATTTTCTTATTCCCCATATCG
1851 GTGGATACACAATATTAAATCCTAAATATGCT
1852 GTTGACAGTACCAGCAATAATCTGTATTCCGA
1853 GTTGGGCACATACATCTATGTCCGGTCAGATT
1854 TAAATGACGGAATAACAACCTCGGTTCCGTCC
1855 TAAGCGCTGGAAAGTCGGCAGCGTTGAAGACT
1856 TAATTGTTCTGCTTCAAGATTAGCAATTAAAA
1857 TACCTTTAAACCCTAATGGCGTTGATTATTCTG
1858 TAGCAGGTAGATTGAATTCATCGGATTGCGGT
1859 TATCAATGTTCAAACAGGGGTTAATGCCTTTT
1860 TATCTGAGATAGAATTCCATGATTCAGATCTA
1861 TATTAGTAGAACCTGAGCATAAGCGTCTACAC
1862 TATTCGAACTCATTTCTACCTTTTGATTCTGA
1863 TCGCCTGTGTCTGGTAATAATCCCTTGGTGTT
1864 TCGTCTTGCTCGGTGAGGTATCGGGCGCACAG
1865 TGCAACCGCGTTTAATATTCCCGATTTTTTGG
1866 TGGCAAAATCCCATGCTTTATGCATCCGAATC
1867 TGTAAAACGTTTTCTTTAGGTCGTCCAATTGT
1868 TGTCTGAAAACTCAGGATGGGAGCGCAATCGT
1869 TGTGCAACAGCGTTTAATATCCCAGATTTTTT
1870 TGTTAATAGAACCTGAGCATAAGCGTCTACAC
1871 TGTTAGCGAGAAATGTTATTTTATCCCAATGT
1872 TTAGAAGCCCTAAAATAAATTACCAGTCTGGC
1873 TTCCTCATGAGCGCGTGAATTGGGTGGAGACA
1874 TTGTGTTGCCGCCACGATACTCGTTTGAATGC
1875 TTTAAAACTCAAGTTTTAGCGCCGTCTGATAT
1876 TTTAAATTGTTGGGTGAGTATTGGGAGTCTAT
1877 TTTCAATGTTCAAACAGGGTTTAATGCCTTTT
1878 TTTCACCGGTGGGTTCTTGCTCTTCGCCATCAC
1879 TTTCACTTGTGAAACGTTTCTCATATGGTAAA
1880 TTTTAATATTATTAATTTCATCCCATTGCAGCT
1881 TTTTAGGGTCGTGTCTATCAGCCCCTTGATAC
1882 AAAATCAGAGTCCCAGCCTGACAGGTGGCTAA
1883 AAAGTGCTCAATGAGAGTGACGCAGAGCTATCGG
1884 ACATCTGGCCCACGACAAACATCGCGAACCGT
1885 TTGGAACGGTTGAGCCATTGCAGCGGCGTAAA
1886 CAACAGCATTGGAAACTGCCGTTACCGCCACT
1887 CATTTCTGTTGTCGCTCAATGGAAACATTAAT
1888 CTGATTCTGCTGGAAATACTTTGATTTATGAA
1889 CTGGCGCGAGTACCTTCGTCCATTTCATCAAT
1890 GACATTACATTTATCCGGCCTGAACATGGGGC
1891 GAGAACCTTTCACGGTATCGGCATATGGCCTG
1892 GATCAGGCTGCATCTACCTCTGTTCTTGGCAT
1893 GCCACCTACAGCAAAACGGTCAGTATTGGATG
1894 GCCGTACGACATCGTGCCGATATCGGTCAGTT
1895 GGTCCCTTCAGTTTTTGAAATTTTACTTTCCT
1896 TCGTCAATGAATTGCGGGACGTTCCGGCGGT
1897 TCGTCAATGAATTGCGGGACGTTCCGGCGGTT
1898 TGATCGCCAGTTGCCTGCGTTGCTGTTTTTACG
1899 TTTCTGATAGCAGCAATACCTCATTGGGTAAGA

1900 TTCCAGCGTGTATTTGAGTCGGTCACGGATAA
1901 TTCTGGATAGGACAAATAGGATGATTGTATCAG
1902 TTCTGGATAGGACAAATAGGATGATTGTATCAGG
1903 CATTGGAACAGGCTCAATATGTTTTTTACATT
1904 TTTTCTTGCTGCGGAGTAACGGCCTTGGACGC
1905 ATTCGACGGTCATCAGTGACAACCTTTTGTAA
1906 TTGCAAATACAGGGACTAACTTAGCGGGAATA
1907 AACAAATTTAGGTGAGGATTTATCTGGTTCTCA
1908 CTATGGCTGTGTTATAAGCCGTTTGTAATTCT
1909 GTATCCTCCGGGCTAACGAGGTACCGCTCCGG
1910 TAGATGGAATACTTGATGATTTCATGCAGTCA
1911 CATAACTCCGGCCATTTACCTGTTCAAATTTA
1912 CCTGCGCTTCCGGCTTATCACGCCATATTTCA
1913 CAGTTGTTAATTGTCATCTCTGAGACACCAAA
1914 TAATTATGTAGCAACCCGTGGCGTTGACCGGA
1915 AACAAAAGTTCTAATGGTGCGAATGTTAGCAA
1916 TTAGTTAATGACGCCGGTTTTGTCATGAATAT
1917 AAGACCACGACCCCAAAGCCGCCAGGCCATTT
1918 ATCGTGCCCCATTAGAGTTCCGATACGTGTAT
1919 TTACCCACATCGGCATTGATAGCACCGGCATC
1920 CGGTGCGCTGGCTCGCTTGCGTTGCGCGACGT
1921 CTCCGACCAATGCCATTGCAACTCTAACTATT
1922 GAGGGCCCGCGCCTTGATGCATATCTATGCCC
1923 ATAATTATCTTAATCATATCCCAATGTATCGAT
1924 GATACCACGGAGCATTGCTTGCAAAGTATCGC
1925 CATTACTGAGGCGTTGAGTCCGGCATGCCACC
1926 TTGTGGCACAGCCACATTCTCCGGCTTTGGTA
1927 ATTTTCAGCAACACCTCCACCGTAAATATATT
1928 TAGATGTTATTGAAGCTAAAGAATTTGCAGGT
1929 AAGGTCGAATGTATTTCGAAGAGTTCAAGTTA
1930 CACTGCGACCGCCGGTCGTGCGGTAACTAACG
1931 TGTACGTTAAAAATGATTTTATTATGTGTGAT
1932 TTTTACAGTTCGATGATGAAGTTTATGCCTAT
1933 CTGGTGGCGGTTAAGCGCGGCGATAGACGGGA
1934 ATCTGCGCTTCCTGAGTAACATCCCAGCCGAT
1935 TATTGCATTGGCCTGTTGCTCGCTTTTGAATG
1936 GACAAATTAATTGAAAATGATGATACGATTAT
1937 ATATCAATCGGGCCTTTCAGCATTGCTGAGCT
1938 AGGGGGCGCTGTTACTCAAGGGTCTGCAAGCA
1939 ATTTAACTAAATCTGAAGATTATAAATTGTTA
1940 GATTAAAAGGTAATGTTCTTTTATTTTCTGGC
1941 TTGATACGACGTCATTCTACGGGTTTGTGACT
1942 TCCAATCAGCGCCGCCCTGCCCGTTTTCAATG
1943 ACAACCGGGTAATAAGGGTGGTTTCTCCATTT
1944 ACTAGAACAAAATCACCTTTATTTAAAGTTAG
1945 GTTTTCACTTTCCATTTAACTATATTTATCAA
1946 TAAAAGCGGTGTCTTCTTTAATTTGAGATTGT
1947 CCATAATCTTGTATAACGCGCTTACGAGTATT
1948 TTGTAAATCCCTAGATATACTCTCTTGATTAC
1949 GACGTCAGAACGCGTTGTGAACATTTTGCGTAG
1950 ACAACTCGACTAATTTCATATCCATTTAGCGT
1951 GGGTTGCTGGTGGTGAGGTTCCGGCGAATATT
1952 GATATCGCAAGTTAAAAGTGGGCTCCCGTGGT
1953 CATATCATCGTATGTATTCGTGCTCGTGAAAA
1954 ACTTTTCACCCATAATCACGTAAGTGTGATCA
1955 TTGAGGCGGCAGGCGCGCCATCCGCAGATGCT

1956 CTTTCTTAATTTGCACCCAATGCCATTCATCT
1957 ACCGATGGCAGTGAGGTACCTGTAGCCATCTT
1958 GCTTATTCTTTTCTCCATCATCACATGTATCT
1959 CTCCACACACTTAACTGACGAGTGGCTGCACT
1960 TGGTGGCGGTGCATATTAATGACCTACTCGCA
1961 GATATATTTCGGCATGATGTGTACCCCTTCGA
1962 ATGCTGAACAGCAGCACACCGCCGCCTTTCTC
1963 AACAGCATCACCAGAAGATGTTGAGCACGTTAG
1964 GATTGCATCGACACCCGATGTGCGCTCTGCCT
1965 ATTTCGGGGGAGGTTGGGCGGGAAACTTTGAAG
1966 TTTTGGCTTCTGAATCTCTCCCTGCTTCGGCT
1967 GGCTTTAACTTGTTTGAGCATGTCGCGATATA
1968 TGGATGCAATAGAAATCGAGTTAATTTCCGGT
1969 TTTCAGATGAAATTATCGTCTCACCTTTGATT
1970 ATGACTAAAGACGGATTTGTATTCCTCGTTAT
1971 AAGATGGAAAGGGGAACTGGACTGTTTGCCGC
1972 TCGGAATTTGTGCAAAATACCGGGTCGCGATAA
1973 CTATCAAGACTTTCCGATGCGAAAACTTCATCG
1974 ATATCTATCTGACAGCGGCGGGGTTGGGGTACG
1975 TCGATATTTCCGGATAAATGGTTATTGCATGGC
1976 TCAGCACCCCACGCATTAGGGGTGCTGTATGG
1977 TTCCATCCCTCCAGAGAGTTCAACCGTGGGGT
1978 ATTTGCACCAGTATCCTGTTGTTGGTTCCCCT
1979 GCCCCTTGGGGTTCGTTAAGTTTGGCATTATT
1980 TGTTGTAATGGTGTTGAACTGCGGGGCATTGC
1981 GCTTCCACTCGCGGCCTGATTGATAGCCTGAT
1982 TAACAGCCGAACCGGTGTACTGGGAGATTGCT
1983 GTAGTCCTGATTAGCGCTGGCGAGCGCACTCA
1984 CTTTCCCTGCCCCGCCGCACACCCCGCACATCG
1985 CTGATCACTTTCCGCGTAAGCCTTCTCAATGC
1986 GGGAATGTCACCTCGGATTTCAGCAACTGTAA
1987 ACTTAATCAGCATTTTCAGGCGCTGAACAATA
1988 TTGTATATCGTGCAGATGGTGAATTGTCATGC
1989 AAGGAATGGCCGATGCTGGAGGGTATCCCTCT
1990 AGCGAAGGCGGCATGTTCCAGTTCCCGCCAGA
1991 ACGTAATGTAACTAATCAGGCCAGTCTCAGCA
1992 CTTACCGGTCGCTTTGTCCAACTTCCCCTTAA
1993 ATTTGTTCTCGCAGCCGTTCGAGCGCCAGATA
1994 TAACGCTGAGTAGCGGTTAATTAGGTTCTTTC
1995 ATGTCGCACCATCCGCATTATTGGCTTGTACA
1996 CTAATGTCATCAAGAACGACTTTAAGCGTCTT
1997 AGGCAAAGAATGATATCCGTTATTACCTGAAT
1998 ATTATTGTTGATCCTGACAAGGCGGTTCACGAT
1999 TGGCCGAAGTGATACAACGAAATGCTATTGCT
2000 CTTCCAGAGTTGAAATTGCTGAATGACAGAGT
2001 GAGCAATTATGTTCGTGAGCCAATTGCATGAA
2002 TGACAGAGGAAGTACACCAAAAGAGCCGCACA
2003 GTAAAAGAGAGAACCGCTACTCATATTGTGACG
2004 CTTAATGGCCCGGCCTATTCTTGTTCGTGACT
2005 GACGGCAGGTTACCTGTTGTAGAGCAGCAATT
2006 TACGGGTGGCCCGAAAGTTCAAGTCATCATGG
2007 CAAAACGTAGAGATAGAATGCAGCCTGAACAT
2008 ATTACGCTGGTTATAATTGTGCAAGTTTGCCA
2009 ATGTGGTTTCACATATTGGGAGTCTATTGGTG
2010 GTACTCCTCTTTTTAGAGGCTTATTTTGTTCA
2011 AACACTCAGCTGGAATTCTCCATCAGCCAATT

2012 ATATTGTCCGGCTATTTCAAGCCGCCTTTGTT
2013 ATATTTATTCCGAAGCAGGACGGAGGCTAGAT
2014 ATGCTGTTCGCAGATACTGATTACAATATCAG
2015 ATGGTAGCGTGGTTGAAGTGCCATATTATTCC
2016 GGTATTGCCATCACAACACTCATTTCCCCACA
2017 ACCAGCGTCATTAAGAACGATTTCAAGCGGCT
2018 TGACCATACCGTGCCGTTTAATAATCTATCGC
2019 GTTTATTTGTGTCTGAGTTTCTTGATCTTGATT
2020 CCCAATATTCACCTAACAATTTATAATCTTCA
2021 ATGTTACCGTAATTAAAACAGGTGGCGCTAAT
2022 TAAGCAGTTCTGCCAGCGCGTCGCGGGACTCG
2023 ATGTTCAATGAGAACTTGGAGGCGCTCAATAA
2024 ACCGACTAACTTCAATCCGCTTCATTATCACT
2025 TTTCAATCAGCCTTTCTATGCTTGCTGGATTAG
2026 CCATTAATCCACGGATGTGATACGTACGCGTA
2027 TTGTTGTGCGTGATGGAATACAGAGTTGTTCT
2028 TTCGTTATGCATTCTCATACCACCTCCCCAGC
2029 GGTTTGTCATCCCTGTGACAATTGCGGATTGT
2030 ACACAGCACCGGTCAGCAGCACTGATGTATTT
2031 CCAGAACTGCTGGCCCGCTGGGTAGATCTACA
2032 AAGAGCGGTGCGCATCGTACTGATGTGCTGTA
2033 GCTTTAGCCCTCTTTGCAGCTAACGCGATTCT
2034 TCAGTATTTCGCGCCATGCCGGTTTGGGATGG
2035 ATTACCTCCGCCAGTGCTGCCGAAGACGCATT
2036 TACGTAATAAATCCATACTGCTATTTATTTCA
2037 CCATAGAAAGTGATTTAAATCCGGCAGCTAAT
2038 TGAATTGCAAAATCAACCAAGTTGAGCAACTGG
2039 TAATTCTTCTGGAGTCATTTCTGTAACCTCAT
2040 GCTGACGTCAGTTTCATCCTCATAACACCTAA
2041 AACTACAGGAAGCTTCTTAGGTGGGGTGTCAG
2042 GTATCGGCTGGCGGGGCACTGGCGATAGCGGT
2043 CTTACCGCCAGCCTTTCGATATGCGCCGGTCT
2044 ATTTCTTTAGCGAATGGCTCGGCACGCCCACC
2045 AAATGGTCGTGGGCAGTAATGTTATTTCTCAA
2046 CAATAGGGTCAACACCCACCGGTGAGATGTTA
2047 GCATCGAAACCAATGCTTATCGCGCCCACGGCG
2048 ATTAACTACGCAAATAAAACGGCTGGTGGCAA
2049 CACTTCTGCTGTGTGATATCCACCCTCAAATG
2050 TATTACAACCAGCAAAGTAATAAACACTCCGT
2051 TTCGCAGCCATCGTTAAGGCCCAGCTGTTAATT
2052 TGAAGCTGACTAATGCCACCAAGAATCGCATT
2053 GATGTGACGCTGGCGGTAAATCACACCATGTC
2054 CTTTTTGCTCTTGCGGCTCTGGCGCTTTCTCT
2055 GTGCCATTTAGAGTCGCTCGGCCAATATTAAT
2056 CCGGAATGGAATCTCATCACATCGCCGCCCAT
2057 GTATTGTACTAGATTACTGTATATATACACAT
2058 CCCTCAACCGGAATTGTAAAATCAGAGTTGGT
2059 TCTAGGCCATTAAAATCATATTGGAACTCAGA
2060 TGTAATGCAAGTGCTTCTGCCAATGAACCCTT
2061 CTGTGACTTAACCGTGTCGCCGCTAGTTTCAA
2062 GTTGGTAGCGAATCAGACAAGATTGCCTATAT
2063 TCAAACCGTCATTTATCAACATGACGCGCATT
2064 AATTAGTTTAAATTTAGCTCGCGTGGTAGCTA
2065 TACCCCGGCCTATTATCTGTTGCAGCAACC
2066 TGAAAATCACGAATGGTATAAGTCAGAGGTTA
2067 AGACGTGGCCTTATATCGTCGCTATCTACGAC

2068 CCGGAAGCACTGGAAGTGTGCGCGTTCGATCA
2069 GGTTCGTCTGGATTATGGCCCTGTGCAGTTTG
2070 GTTAAGGAGTTATTCCTATCCATTCCGGCGCA
2071 CTTCCGCCAAACGTCCCCGGCGCTGGTCGACC
2072 TGCTACGGATACGTTATAGCTGCAGTGAAGAT
2073 GATCACCTCCTGTATGGAAATGAAGTTGTGAT
2074 AGAATGTAACCGTTTCCCAAGTTTTAACAGAGT
2075 AAGGCATTAAGCCTTGTTTAAACATTGACAGA
2076 TGTAATCCGGCAGGCCGTAGACTTGCTGTTGT
2077 TGCTTACTCCAACTGTAGAAGTTATTGCTGAT
2078 TCGTTGGCCTTTACCGTGGTGTTGCGGGTTGT
2079 CCAAGGCAACCGAGGATTTCACCCCTGGGCGG
2080 ACGTTCGGATTTGTCACTGCCACGGGAGGCAT
2081 CAGTATATGAATATGTTCGCTCTGGCGGTCGT
2082 CGGTTCAGGTAAGTCCAAAACGATGCTTTCAC
2083 AGATTAGGGTAACTTTCGATAATATTTACGAA
2084 ATTAGCCAAATCAGCGCGTGCTTTCTCGCTCA
2085 TGAGCGGTTTCTCCATTCATTATTCTGAGAAG
2086 AGATCCATCATATAATCTGGATTGGTAATTGT
2087 ATATTTATCAGGGTATCAAAGTTTATCACAAT
2088 TCCATATACCCCTCAATAACTTGTTTGGTAGCA
2089 AATTATTTAGATCCATCATATAATCTGGATTG
2090 GCATTAGCAAAACTACCAGAGGCTCACCTGGT
2091 CCTGTAGCCAATTAGCCACTATTAGAGGGTAA
2092 GTGTTGACCGTCTCATCTCGATGCAAATCATC
2093 CAGCTCGCAACAATGGAACGCCCGATGCATAT
2094 TTTTTCAGTCTCAAGAATCCACAATCCAACGC
2095 CGCCACCCGCATGAATTACATGCTTTCCATCT
2096 AGGGGTCAAGCTCGGGTTCGTGATAGCGTGTC
2097 GGCTATTGGTACTGGCCCACCCTGCCCAACTC
2098 TGACCACGTTCGCCATCAGGAAGAATTACAGA
2099 ATTCATCTGGCGTGCGCTCTTCCACTTTTACCG
2100 GCCTTTGTTTATAGCATGGGCGCTGATATGGA
2101 TCGGATGTGTGCTTTCATGGCTTCTCCGGCGC
2102 GTTCTTCATGTTCTCAGCCTTCGTCGCCATGC
2103 TTGGATGTGGCCATTGATGGGGTCATGTCGGT
2104 ACAGACATACCCTCGACCGTGCCGTGCTTCAT
2105 TAAAAGAGTTCTTTCTGAGCTAATCGAATTAT
2106 TATCCCAATCGCCAACCGCGCCTATCGACTTAG
2107 CAGAGAAGAGCGAGCAATGGCATCCGGCAGCA
2108 TCGGATTGCCCGGTACCGGCAAAACAACTCTA
2109 CAGATAGTACAACTAAAAACGCTTATAACACT
2110 TATCAGTGCGCAAGACGTTTTGACCGCGCA
2111 TAGGGAAGTTAATCCATTTTGCTTATGAGAAT
2112 TCAGTGGAAAAATGAATCGGCATATCTTGCGT
2113 CACTCATGCGGAGTAAGTGTGTGGAGTTGATT
2114 GCCAGCTTGTAGCGTCGGCGCACCGCATATCA
2115 ATAAACGCCGTTGCCGTCTCAGCATTGATAGA
2116 AGGTTATCAGCTTCACCTTGTGAGTTCGATAT
2117 TTTTCAGCGTAGGCCTATGGGCAGTCGTCTGT
2118 GAAGAGTTTCAGTCGGCCGGTGTAAAGGTTTT
2119 AATTTGGATGGCAAGCCTTATACGGCGCGTGA
2120 GCGCTGCAATCGTCGGTATCGCGTTCTGGACG
2121 ATGCATGCGCAGCTATTCGATGGTGACAAGGT
2122 TAAGCAATAAAGACATGCAATCTGGCGGCGGT
2123 CTCCGCTACCGAACACTGAACGTAACGCCTCT

2124 TTATACCACGGCCACCAAATAAAAAAGCCACCC
2125 AAAATAACCACGCCAAGGATGGTGCATTAATA
2126 TTGGAGGCACAAAGATTGGTTACATTCATCTT
2127 GCGATCAAAACGGCAGTTGTTGGTTTGCTATA
2128 TTGTCACAGAGAAACTTCTACCGTATGCAGGT
2129 TCTGAACGAGGCTTATAAGCCACCATTGCTAT
2130 GAAACCCTCTAAAATTTCACATGTTTAAAGTT
2131 GACGGGGCGCTAATTCCAGCGCGCGTTATTTT
2132 TTCCATTTATTAAAAAAAAGCGCCTAAAAGTA
2133 TTTAACCAACCGTTCTATGCGTGCTGGATTAA
2134 TACCGCAAGTTCATACTTGCTTCCAAATCTGCG
2135 ATTTGGAGGAAGAATCGCAAGGTGATCCCATT
2136 AGCTTGTCAAAGTTCGCGCTGGTCGCCCATAA
2137 TAAAGCAAAATTGCATCTTTAGTACATTCGTC
2138 AGTATCCAGAATCAGTAGTTAACTCACTGAGCC
2139 ATATCCGTAGCGAGTCGCACGTACTCTGTGAC
2140 AATGGGAGGTGATGTAATGAGCAAGGATGATT
2141 GCATGAGTGTCGTGCTCTAGTGGCTCGAAGCT
2142 TTCATATATTGTACTGATGCCTTAATCTCATTA
2143 ACTGCCTGAAACATGAAGCTAGCCCGGCAGGG
2144 AAAGAATATCAGTCGCTGCTGTACAGCATACGT
2145 GTTATTCTAGAATATTCATATATTGATTTATC
2146 TATGACTAATCTCGCAGATGTCGCCAACTTTG
2147 CATCACTGAGCTAGTGCGGTGGATAGCGGATT
2148 AGTTGGAGATTATTTCGAAACTTGGTGATGGC
2149 TATCATATTCGCGGCGTAATGATGAAAGTTCCG
2150 ACGTATTATTAGCTATTACACTAGGGAGAATA
2151 ACAGCGAAAGGTCGAGCGCGGATTGCATCACT
2152 TAAAATATAAAGTTCTCTATAAGTTGTTTAGTA
2153 CTTAAATTTCTAGCATTTGCGGCGTTAGTTGC
2154 CTTTATTTCTGGCTTGAACAAATTTCCATCGG
2155 TGGGGTTCCCTCGCCAGAATCAACAGGCATTA
2156 TAGCCAACCCCCTTGTTCTACCAGCTCCCTCT
2157 TATAGCTGATGATAACTGCATGTTGTTTATAT
2158 GTAAGTTATAAAGTTCCTATCCGCAATATTCA
2159 TCTACCCGTTGATACTATATTCATTCAATAACG
2160 TTATCAGTGTAAGTGTTAGCACTGTTCTCTAC
2161 TGGCTCGCTTGCGTTGCGCGACGTGGTGGAGA
2162 TGCTTTCCTTGACAGTTTTTGTACTCCATTTG
2163 TGTCGGCGTGGTTGGGTGAGCGGGTGCCGGAT
2164 CAGAGCTGCTGGAGGCTTTGATTAATCTTACT
2165 ATACTGCCGTGTATGTGTGTATATATTAAAAT
2166 AATACTGGACAGTTGCCAGCTGGCTGACTCGC
2167 TCATGGAGCCGCTGAGCCTTTGATTTTTCATT
2168 CTTCCCCTATCTGTATCACCACCCCACCGTCG
2169 TATTTCATACTCAATATCTTCAATCCGCTCTT
2170 GCCTCGCCGTAACTTCATGTGAACTAATGGAT
2171 CATTCATCACAGAGAGCCTTTGTTGCGCCGCC
2172 ATTGGTGGTCACTGTGTGCAGGGGGCCATTCA
2173 AATTAATATCTACTTCATATACTTTGCTATAT
2174 GATTGACGATGATTGTATCACATTCAACTGGA
2175 TAGAAGGATTCGAATATAACGAACTATGGAGC
2176 TTTTTTGCTGGTGCTAAATTGCTGCGGTCAGT
2177 GGCCAGCAACACGCACAGGGGAAGCAGCAGGC
2178 CACATCATTGAGATATTCATTCGCGACAAGC
2179 TATGATCACAATGTTCACGCCAGCAAATTGAA

2180 ATTCGCGTGATTAACCAACTTTATTAGCACCG
2181 TTTCAATGATTTACAGTGGCTGGGATGATCTA
2182 GAAAAGTAATTAGTAATAGCGATATGCAATCC
2183 CTGCTCTATCTGCGCATCAGTAACTCCAGCGC
2184 GCTAATCGCGCTGAAGTGGCGGCGGTCTATAC
2185 TTGCAAGCGGTTAAGAATATGATTCTTGGTCA
2186 GGGCCATTACCAACAATTGAACGGATTGATGA
2187 ATCCACCCTGTTTGCGAGATGATTGCGGTTCA
2188 TCGGGGTGGGGCGGGTGACCGGCAGTCATGAT
2189 TTGTCTTCTCAATAATTATCGCTAATGATTCT
2190 GGGACGCCCTCAACGATAAATTGCGCCGTAGA
2191 GAGTTAGACAATATAATCAAACTCTGGGTTTA
2192 AAATCTGTGTAAGCTTTTAATACTTGTGATAA
2193 GTTGGCGAGGCTGCTCTTCGTCTTCAATTTCT
2194 GAAAACCCATCACGAGCAGCCGACAAAACGAT
2195 ACTTACATGGGCCACAAAGACGCCGCCAGTAC
2196 TAAATATCAAGTGGGCCGGATCAAGGAATATAT
2197 CAAGTTCATGCAACGGAACATCTTGGCTTCAT
2198 TATCTCTAAATGAATCAGCTAATGTGCTGCAT
2199 ATTTTGTGTTCCTTTCTGCGTTCCGTTTCGAT
2200 AAGCTGGCTGACGCCCACGGTAAATCGAATGC
2201 AAGAAATCAGAGAAATGCTTGATTTCATTGAT
2202 ACCAATCCCTGACGGAACGAGAGTTATGGGTGC
2203 CCATAGCTAGCCACGCGTACACGTGGTTCGTT
2204 GGATCGGTTTGATTAAGCGGCCACTTTAACCA
2205 TTGATGAGGTGACGACGCGGGCTAAGTTTGTC
2206 TTTGATTCATAGAAGTGTGGCTGCGTCTCGCGT
2207 CTCATGGGGAAGGCGCTTTGACTACAGCATCA
2208 TAAGCAAAAATTAGTTTTACAATAAACCTCAA
2209 TATCAAACACGTCCCCAACCTTAAATTTTGGT
2210 ATTAAAAGCAGCGGCAGACTCAGAAATTGACT
2211 TTATGGAAACCACGGGTTCGGTGGTGCCGTCA
2212 TACTTTTGCTTGAGTTTTGCGGCCTCTTCACT
2213 CTTTTAATTGTCATTAGGTTCTACTCCTAGGC
2214 CTCAGTGCGCCCATTCTCCGGTGTAAATGTGA
2215 ATCAGAAGTTGGGGCATCATGTTTACTGTGTT
2216 GCACCGAGGCGACCGCTGATATGCGCTGGGAA
2217 GCGGTGTATCCAGATATCCACGTATCATAAAT
2218 AGCGTGTCTATTACGAGGACGGCGTTCTGTAC
2219 TTATCCTCGGCTTTTTTGTTTGTGCGATTTGT
2220 CAGTTGATTTGGTTTGCTGATGCACCCTATCA
2221 CAATCAGCATCCGGGCGAGCCACTGCGCGACA
2222 CACTGGTACACAAATCGGGAAGCTGTATTAGT
2223 CTATTTCCTGTAATTTTTCTTTTTGATAATCA
2224 TCTTTGCCGATGTAATACATGCAGTTTTTATAT
2225 TGATACGGTCGGAGGTAATACTGCTCCAACAG
2226 ATGTTGGCATTGCTATTTCAAACTATCGTGAC
2227 ATTTAATGTCCAGCAAATTGACGGCTTACTAC
2228 TATGGCGAACCAACAGCTGCCGCTTTGATCGT
2229 GCGGTTGCGGCAATCCGCTTCCAGCATTGGCC
2230 CCACTTATCTGATCCACTCAACGTAATGGTCA
2231 AGATCGGATTATCGTCCCGCGAACTGGCGGGG
2232 GTCAGTCATTACCTCTCCGGCATCTACTGCCA
2233 GGTTGCTGCGATTATGGCTTTGCTGCGTGGCT
2234 CGCTATCGACGTGTTACCCACCGGTGCTGACT
2235 AGTTCCCATACTTATTGAGCTTTATAGCTTTT

2236 CATAAAGAAACCTTTGACGGGCATTGAACTTA
2237 TACTCTGTCGAATTTTGCTTGCAACAGCCCAT
2238 TTTTCTTGGTTTTATCTGCGCCGTTGCTGGCT
2239 TACTGGGGCAATCAACAAGCCGCATGACCGTT
2240 TTATATCGCATCGGCACACGCAACATTCGAGGT
2241 GAAGAAGACAAGTTAACACCGGTTCGCTCGCT
2242 TCTGAGTTACAATGAAAAATGAAGCGTCTAGC
2243 AAGTTAGGCAATACAATCAAACTTTGGGTTTA
2244 CATATCATCACCGCGCTGTATGCACTCCTCAA
2245 GTCGACTGGCGCTAGCGCTGGCTGATTCCCTT
2246 AAGTATGCTTTATCCAGTGCTTCAGCTCCCCAT
2247 TTATATGTTTGCTGCATTGCGTAATCACCACT
2248 CATGTGGTTTTTCTCTTCATAATTTTTCTTAT
2249 TATTGACTATTAAGTAACGTGCTAGTGTCAATT
2250 ATTCAACGTAGTAACCCACGCCAATCAAATCTGG
2251 ACATCTGTTCTGAGATGGTTGTGACTCCGGAG
2252 CAGGAGTTGTCTTGTTTGTCCGGGTATTTACA
2253 TTGGCCGTACGGTTATGAGCTGGGAGGCTGGT
2254 CCTTGTTCATCCACGCCGGCAATGTGATTTTC
2255 ATTGGTTCATTATATGCGTTAAAAGCTGACAC
2256 ATCCGCACTTCCTGAATTAGCATCACTTTTAA
2257 GTTTTCTTCACTCCTTTTAAAGCATCTACATA
2258 GGGTAAAATCATATAACCATACATTTTATGGT
2259 GACATTTTGAGTTAATAAATTAATAGTAGTTT
2260 TACCGATCATTCATAATGAGGGGCTTCTCTTT
2261 CGTAGTACATTTACTTATGTTAAAACTCGTGA
2262 CAGTTATCCGAAACAGAATGGGCTTGCCAAGG
2263 TTTGTACCGCTGATGTTCTCCCGATAGTAGCT
2264 ATGCCATCCGTAAAAATGGTTATTATGAAGTT
2265 AAAATATTGCCATGTTGGGTTCGCCGGGAACC
2266 TGCTATTCATCATAAAATGGCTGGCAAAAGTG
2267 ATAACATGCTACCTTATTTACCTTATTTAATT
2268 ATTATAAAGAATCACAAAATTGGCGTACTAGA
2269 TTATTGACTCAATTTAATAGGAATTATTATAT
2270 AAATGACAGCTTCTATGATGATGTGGAATGAA
2271 CGCAGATGAGCTATTATCAGTCACAAATGGAT
2272 ATCTTTCCATCAGTACGTCGCATTTTTTTACGG
2273 TGGTTGCCCTTAACATGACCCGCTTTCTTCTT
2274 AAGCAATCATCGGGGTTGAGATTGCGCAAGCT
2275 TCAGTTTCGAAGAGGCCCATGGGGGATTTAAC
2276 AAGTCAAGTATTATTTCCCCTTATAATAAATT
2277 TCTGCCAATGCCGGGAACTTTCAAAACACCTA
2278 TTGCGTGGTGGTTCATCAGAGCAGCCCGATGCA
2279 AATTTAAGAACACATGTGCCTTATCAGGGCGA
2280 GTTGAAAGAACACATGTTGATGATGATTGGCG
2281 AGTAATACAACATTCACAGTTTTCTACATTCA
2282 TTCTGGATTGGTGAAATCGGATGATAGTATCA
2283 TATAACATTCAACAATTAAACTTTTAATATAT
2284 AATTTATGAGCCACCTTGTCAAGATTCGCCAC
2285 GACCAGATAGATTAAGTAAGCGTGATGTCCAT
2286 TCAAGACAGCATCCTATCTGGCAGCCCAGACT
2287 CGGGAGATGAGGTTTTTGTTAGGACCTTGGAC
2288 GCAGTCAACACCCAACCCGTTGAACCCGCGAT
2289 TTGTTATTGAGCCTAATACTCATATCCACTCG
2290 TTACCGCGGGTATGGCTGCATCCACGCTCTCA
2291 TTTTGGCCTCTCAGTACTTCCATTACCATTTT

2292 ACTCTTTTTAGGGTCGTGTCTATCAGCACCTT
2293 ATAAATATATGGAATATAATCTTTTCTTTAAC
2294 AATAATCCAAAATCAGATTGCCTTAGTCTTGT
2295 TTGGTGGTACTTGGGGCGGTAGATTCAAAGAT
2296 CCATTAATTACTACAAAACTCTGTTGGGACTT
2297 CTGTTTGACCGCCCGGCAGAAATCCCCATCAC
2298 GCATCAGTGCGAACGGTGCGCCATTTTCGACC
2299 AACATTGATGGGGTTTGTTCCCGCCGAGATGC
2300 GGGTTTATTTAATCGACTCGTCCAATGCGCTT
2301 CCTTTTCGGTAAGGCCGCTTCGTTTTCGATGA
2302 TTGAAATGGTCAATAAGTATGCTCACCTGAGC
2303 ACCCCCCCCCGCAACGTCTGCGATAAAATCGAC
2304 ATCACTCACGTTATGAAGTTTTTTGAGCGCGT
2305 GATACTATTCCTCCACCGGCGCATGCTGGTCT
2306 TTTACAGAGTGCGCGAGACATCTACAAAGACA
2307 ATCCATTTCGAGCTTTACTTCTAATGCCATCA
2308 GCGGCAACATCACGCTGACGTGTTTGCATGTC
2309 ATGACCAAGCTGACTGGGATAACTTTCAGGAT
2310 GCCCGAGCACAGCTGGGCCAGCTTGTTCATCA
2311 CTGTGTTAGCTAATCATGCTAGCAGTGTACAA
2312 ACATCGTGAAGATTAAACACAGTTCCTAGTGT
2313 CTCCACCGAAGCAGGGGGACCGGGTATTTATT
2314 TTGATGCCAAGTCGTTTAAAGAGGTCACTGCG
2315 ACTTGATGCGTTGCGGGAACTGGGTGCATCAGG
2316 TTCCCAATGCCGGGACCAGTACGGGGGCCTGT
2317 TAACGCTATTTATCACGTCATCATTATCAAAC
2318 GATGCGACGCCCTCATGAATATGGAGTCGCGT
2319 GTGCAACGTCAGATAACTTCTCAGCTAAAAAT
2320 GATGAATGACTTGGCATGGACGCCGAGGGAGT
2321 TGAGTGTCGAGGTACTCATCATCCCACGAGAG
2322 CGTAGAACTGACTTGATACGTCGGCGTTAAAAT
2323 ATCACCGCGCTGTGTCTGGCCTTTGACGATAT
2324 GTTATCTATCGGAGGAATGGCTGATTTACTCT
2325 CCAGCGCCGCGCTTGATGGGCTGATTGCCAAG
2326 CGCTGCATCATCTAGCGTCTGAAAACGTACTT
2327 TCGTTTCAAGGTGAAGCTTATTATCATCGGAT
2328 ATGGTTGGCGTCTTCATCCAGTTCTAAATGTT
2329 TTCGCCCAGGGCGGCAGCAGCGGTAATAGCCT
2330 ACAGATTTTTTAAAGTGCCGTTCGCGTCAAAC
2331 TTGGCTTTAGCGCTGATTATCTTGTGCTTGCA
2332 AGAGTATGAACAGGCGATCACATTCCATTCAG
2333 TTCGGGAGGCGTTACGCCAAGGCTATTCGGTA
2334 GGAGGCAACACGGCGCGATGCAGAACAGAAGC
2335 CGACTAGCTGTACAACGCGCCCAGCGGCTTCA
2336 AGCATCTTCTTGCGCCATTGCATTCGGTCTTT
2337 CCATTGCGCTTTGATGAAGAATTAAGCCGTCA
2338 GTTCACTAGGTGCTGTAGTTCTTAAAGGTGAT
2339 CAAACAGCAACAGCCCAGCAGCGGCGCGGTCT
2340 GGTTGGGCCTCGGGCAGCGTTACCCGCATTTT
2341 GGACCTGCACTTGTCGTGTGGTTCTATACCAT
2342 GTTTCTTGCTACTCGCTGCCTAGTTTCACGCT
2343 AATTCAATGTATCCAGCACAAATGTATTTTGA
2344 TACGGGGACAACATGATAATACACTTCCATTA
2345 GATTTGCAGATCTGCAAAAAGGGGACGCTGTA
2346 TATCAAAGCACACTTATCTGATGTTTGGTGGC
2347 AGAACAAAGCCGATTAGCATTAGTCCTCCACT

2348 AATGGGCGGTGGCGGTGCGGTTGGTGTGGGTT
2349 AGCCAGCGGCACGTTTATTGATTTGTTTGCCT
2350 GATACCTGCAATGACGGGTATTGAGCTGGAAA
2351 ACTGCCACCCAGCGCGGTGGCGTTTATCTGAT
2352 TTGAGCATTCAGAATTTTGATTGTGGCTTCAC
2353 TTTCTCCCAGCCAGTCACGGGCTGCCCGGACG
2354 ACGCCTTCATCCAAAACAAGTTCACCCCTAAAG
2355 CCTTCGTGCTGATATCGCTAATGGCACTAAGCG
2356 TTCGCTGGTTTGTCATTCGGGTTATCTGTCCA
2357 ATGCTCCCCAGGGCAACGTTAGTAGCCTGCTC
2358 TTGCGTAGTTTCCTTTCGTTCTCAAGCTGACGC
2359 GTCTTGAAATGCCTGATGTACAGCTTGGCGAA
2360 TGTAATCCGCCGGATTGTAATGTTCCGTTTTC
2361 GATCACTGCTCTTGTCATCGCCCCGGCACTAT
2362 AGCCAGCGATGGGCGGTATCGCGAAGTAGATC
2363 GTTACCGGCGAATGGGCAGGCGGCGTGTGGGT
2364 ATCCTCCAATGCCAGTTGGATTTACAGCTACA
2365 CCTGCTATGGAAACAAACTCATTTGTGGCAT
2366 AATGTCGTCAGTATTCTCATTCCTCTCATGGT
2367 ACGGCTCCTTGCACGTAAGGATAATTTCGAAC
2368 AAAACTGCACCCCCGGCTCCTGACCGTACTTC
2369 ATAGATTTGTTGGATCGTGATGGCGAAACACTC
2370 TGGCCGATACCCTGAATCGAATTAGCGCGGCA
2371 AATTCAATCGAACGATGGAGCGCGGCAGCTAC
2372 GGTAAAATTCCAACTAAGAAGTATGATTATGT
2373 ATATTCGTTACTATCTTAATGGCATTTTCCTT
2374 GCGAGGATTTAACATGAGTGAACAACGCAAGT
2375 GATGCTGGTGTCGTTCCGGTTCAAAACTCACT
2376 TCGGTGCTGATGGAACGCTAGCAGCAACTAAC
2377 GGGCAAAGTCTGGCCTTGGTTCGTTTCTTGTT
2378 ACTTGATGGCAGGACCATTGCGATCAGTTGTT
2379 ACGGCTCTGTTGCATCACCTGCCAACTCTACT
2380 CACTTATGTGGGCTTTTTTATGTCTGCGAATT
2381 ATTATCTAACGCCATTATATCCATATCAGATG
2382 CAAAAGTTCTCTGCGATGAATGGCTGGTTACC
2383 CTTCAAGCCATACTCGCAAGGCCGTGCACGCT
2384 ATCAGCCATCCAGCCGGGTGGCTTTTTTATTT
2385 TTGGCTACAACGTCAGTAAAGTAACTGCATTC
2386 AAATTTGATGAGCCAGTAACTCGCACCGTATC
2387 ATTTATCAGTGGTTCGACATGATGGCAGAAAG
2388 ATGAGATTGTAGGCGTATATTCCACACTAAAT
2389 TTAATCACGACACGGACGAGCTGGTCGTCTGC
2390 AGCACTATTTTTCCCATCAACTAAACGGCGAC
2391 TAGTCAATCTCTTCCTGAGTCGCTTCACAATT
2392 GTGTCGAGATTCTCAACCTGCTTTTGATACCA
2393 CTGACGTTATAGTCAACGGATTGCTATCACAG
2394 TCCGCGCGTTGTTGGATGTCGAATTCTTTGCC
2395 ATGCATTACCTTGAGCTTTGCCAGCACAGAAC
2396 GGCTTGGTGTCAGGGTTTGGGCCACGATGAAA
2397 CGTAAGATTCAGCTCTGAGCGCTTTTGTTTGA
2398 GTAATATCGGGATGAGATAACGCGGGTGTCAT
2399 TCGCATACGTGCAATTGTTTCTATTGCACTAA
2400 TCTTGCAAGTGATTAACAGAAATATCCGGCAC
2401 CTTGATGCTACAGCAGCATTGTATGGGCGACT
2402 TGGATTGCAGCGGAAGAATCTGGAGAGATTCA
2403 CTCCAGAAATTGGGGGTTGGTTCGGTAATTGA

2404 TACAGTGACCTGACTGACGGGACCACCATTCC
2405 CAATTTGAAACCTTTTTTTGCCCATTATTATG
2406 GACCCTCTTGTACAAGGGCGTTAACGTAAGCA
2407 ATTAATGCCGTTATTACCGCCGTTAATTAGCTG
2408 AGACTGGTCACCCGTGGCTGACGATGCAGACC
2409 AATTGTGCCTCACCGCTGATTTTGGTTTCACA
2410 TTACGGTCGCCCGCATTGAGTTTTCGGTAAAA
2411 CCATCAAGCGCGGCGCTGGCCTCGTTTAGTTG
2412 ATGGCATTGATATCACTATAGCCATGCCTGAT
2413 ATAGCATGACGGATATGACATCCGCTATTTTCT
2414 TTTTAGGGTCATGTCTATCATTCCCTTGATAC
2415 TTAAGTCATTCATTTATTATCCTTGTTTACGTA
2416 AAAAACCGACAGAGAGTCTGTATTAATCGTAC
2417 GCTGGGGGATAGATTACAAGCACAGCCCTGAC
2418 GTTTGATAAAGAATTAAAACTTATTTCGATAA
2419 CATAACTATTCCTCAGCAGATTTGCTGTAATT
2420 TACAGCCGCCCAGTCTCGCGCACTACCCTGCG
2421 CCCTTCTCTCCGGCAAACAGTATGGCCTTGAT
2422 GCATTGTTGCGCGATGCTGGATTCCACGTAAT
2423 TGGAAAATGAAGGCCGTAATACCTCATGTGCA
2424 TTCCATTATAGCCGCCGCCGTACTCATATTGT
2425 TTCGAATGCAGCGTACCAAGTCGCGCCGATTT
2426 GTATAATAGGTATAGTCAGGTTTCTCGCAAAC
2427 GCGGTGAAGTGTGTAAATGATGCAGGGGTGAG
2428 AAAAATCAACAGTGAATTCGTTTTCACACTCAG
2429 ATCACGTTCGTTGATAGTCCCACTGCGCGTCC
2430 TGCCAAAAGATTTTAGATGCGGTCATTTAGGTA
2431 GTTAGAGTGGGAGGAAATCTGGCTTGAAATGG
2432 AGTGACATCATGATTATTGGACTCTTCAGCAA
2433 TAGCAAGCATCTTACCCTCTACCCAGCAATCA
2434 ACCTAACCTTAGGTTCACAACCTATCTCATTA
2435 ATAGTTAAGTTCACTAAACGAGGTATCAATAA
2436 GCTGTTATAAACATCTTTGAAGCTGTAAGTAT
2437 TACGACTTCCGCCCTGCAACTTATGGCTTTCA
2438 ATTCACACAACGAACGCCCACCCAACCGGGAG
2439 GCCTCGGAAGTCACCGTATTGGGTGAGAAGTCA
2440 GGCGGGGATTGGGGGTTTTGGCTTGGTTCGAT
2441 GAGTTAATAGAACGTCAAGTTGAATTAGAGAT
2442 TGATCGGCGACACCAACAACCCAGCCCGGCAA
2443 TTACTTTATCCACTAATAGTTGGCCTGCTGTAG
2444 GTGGTAGTGAAGCGGGTGCGGCTGGTCTAGGT
2445 CTTGCGATTGCGCCATAGTCTGACTAGCCAAT
2446 GTGCCTGAAGATAGGATCGTCGCTAATGAGTT
2447 GAAGCAACGGTTGATTACGCGTTCCTGAGTG
2448 TGGCCGGCTCACATATTTATGCCTACAGCGCT
2449 CCAGCGCCAAGGCATCAGCATTATCGGCAAAA
2450 CTGCGCCGTGGCTATTTCTTTGGCCGCTAACT
2451 ATTGCTCTGGTGCAATTGTAGAATCACCAGAT
2452 TTTTGATGTGTCAATATCAATGACTTGAATAT
2453 TCGGCAAAAGCCTGGCTGAGAACGGAATAATT
2454 ATACTGTATACACGGATAACGGTAAAACTTTAT
2455 CTTTTGTGATTGTCGCGCCGCCGCTCTATGAC
2456 TAATAAAGGGTTCTACGACTATAACTGGCGTG
2457 TAGCGATTTCATCTATCATTGCGCCGGTTTTG
2458 CCGTTTGGTACGCCGATTTCACGGCACCGGAC
2459 CCACCGTGGCTACAGTGACCTCTGGCAGCAAC

2460 ACCAGTATCAGCCCATCCCTCTTAATATCTCT
2461 CCCTTAACTATGTGGTGGGCTTCTAAATAAGC
2462 TGACCGGTTGCCAGTGTTGGTTGCCGCTGACC
2463 TTCACCTGCTGAGATAGCCATCCTGCCAGATA
2464 AATCCGGTTAGTTTCGCCTTGCTCATTGTGGC
2465 TGTCCATTACTGGAACGTGATATTGTCTGTCC
2466 CTTAAAAGCGGCTTCGGGCTGGAATTGTTCTT
2467 GGATCGGGATTTGCTACTCGTCAGACTCGCTT
2468 TTATCAGAAAACGATACGTACTTACATCAAAT
2469 ATTTGCCCGGAACGGGAACATTTATTTTTCTT
2470 GCTATTTCGGTCTGGAAGCGTTCACGCGTGAT
2471 GAAAGTATTAATCGCAAAGCTAAAGAGTTGCT
2472 TATCTGTACTGTCGGCACATTTGTAGTGAATT
2473 ACGCTTCAATAGAAATGCTGGAGAAAGATGAT
2474 GCTCTCTGGCTACGGAAATTAACAATCCCTTC
2475 CCGAACGGGCAAAGAGTTGCGATTGAAACAGA
2476 GCACTACAGGGTTACACGGTTAGTCCGCCGTT
2477 GGCGTTGATGCAGTTTGATGCGGTACTGAGTT
2478 TTAATGGCCGGCTGATGGTTGGGACAACAGGCA
2479 GTCCCATGTCGGTTACACCATCATTTATTGCC
2480 CTGAACTTGCAGAGGCTATGCCGACATCAGTC
2481 GCAGTGCAGTTGGCCCATTCGTGCTGGCGAAG
2482 GCCACAAAGGTAAGCGCATTGCCGCTGGCAGT
2483 GGCAGGGTTACGAGTGGCATATACCGGTGGTG
2484 GTTGCAGAGATGACCACTTGGCGCGCGGCACT
2485 TAAATTCCTAAAAACAACACCCGACAATGCCG
2486 GCTTTGGCACTGATGAAGACGTTGATTGCTCA
2487 GCAATGAGTGATTTTGAGATTAACAAGGCAGT
2488 GCGCATCACTTGAGAGGGCTTTTAATGGTCGC
2489 TCACCGCCCTTCGGGTCGTAAGCCAGCGGGCT
2490 CTGGTCACAACTGTCGGGTCTGATGCCTGGAT
2491 GGGCCATAACAGACGCAGACGAACCTACAGCT
2492 ATTTATCTATACTAAAAATCTTTTTGCTACTT
2493 AGTTGGTCCCCGCGTTGTGGGGCAACGAAGCC
2494 GTTGTTGTGTAGCTGCGGCACTCCATCGCTCA
2495 ATGGCTGGTGAGATTTATTTACCCTGAACAACC
2496 GCCTCTAGCCTAGCATTAACATTATTTTGAAG
2497 GTAAATTAATTCAGAATACTGGAATCTCAGCA
2498 GGGATTAGTGCTCAGGCAGGGAGCTTTCAACAG
2499 CTGATAATGTCCACACTGATCGGCGTTTCATT
2500 CCCACCCCCCCAGTTCCTTCAGCACCATTAGC
2501 GTCAATATTGTGGCGGCCCTGTTATCTGGAAA
2502 TAAAAAACCCCGCACGATGGCGAGGCTCAGAT
2503 ATTGATGAAATGGACGAAGGTACTCGCGCCAG
2504 CATAAGAATCTCAGCTTCTTTACGTTGATTGT
2505 ATCAATGATGAGGAAAACCTCGGCGTTGGCTA
2506 CCCCTGCCTTTTGCAGCCAGTCGCGCCACTCT
2507 AGCCCGCCCCGCACGATAAGCATTGAACAACG
2508 CACTTGTTGATGTGACTCTGACAAATGGGATAA
2509 TGAAGGTATGGAATCTTGTGACCAATGGGTTT
2510 TCGCGCCAGTATGGATGGACAAGTTCCAGCGGG
2511 TGGCTTTATTGTGGTCAGCTTTGTCGTATCCGG
2512 TTTTGCATTTGGATTCTCCTTGAATGCCTCACT
2513 CAGCCAGATAGCCGTTTTTCACAGTATTGATA
2514 AGATCATGGGGCCGAGTTAGAACATCAAACAT
2515 ATAGGCATAGCACCGAGGCGGCGCGAAAACAG

2516 AGGTGCAACAGGGACTTTAGGATAGAAAAGTCC
2517 ACTTAGGGACATTAGCTTGGGATGTGAAACAG
2518 GTGTGGGTTTCGACATCCAACAACTGCCAAAT
2519 AAGCTAAAGGCCCGCCGTTTGTGGTGGTACCA
2520 ATGTAGTTCCCGCTGGAACTTGTCCATCCATA
2521 CCTTTCCCAGTAGAGCTGAACCATCTTTATCA
2522 GTTTGCTACCATCACCGCCAGTAGTGTATCCC
2523 CTTGACCCTCAAATTGAGTGTAAAGGGGTTTGG
2524 GGTAAGCTCTGCATTTAACGCTGTTTCGACGG
2525 TGTTTCCACGTTGCAATTTTTCACCATGCTTA
2526 ATTGATGACTAGAATACACTAGTAAGTAATAAC
2527 GCTCTGCGTCACTCTCATTGAGCACTTTAACC
2528 TGCGCCATTGGCGTTGGTTTTCAGGTATTCCAG
2529 GAAAGAATAAGGATTTATAATTTATGACCACA
2530 ACCAAGCGGAACTGCCTCAAAAGCACCGGTTA
2531 GTGGCGATAAACTTAAGCTGGGTCAAGATTAT
2532 GCATAACCGCCGAGGACGCTGTTAAATACTTT
2533 ATTTATAGCCGGTCAGAACCGCATTTTGCCGA
2534 CGTTCTTCAACTCTTCGATGTTATTCATCAGC
2535 CGGATGTGGGTCATTTATTACCTCTCGAGTCGA
2536 TTTATACGTGAAGGCGTTCATCACAATGCTCT
2537 ATCACCTACTTTCATGTCTCTAATATCACGTT
2538 TCAGCTTGGATGTGGGCCAGTGTTGCCCCTGC
2539 GACAATCACAGTCCCCCCGCGCGCCTGGCTTG
2540 GTACGTACCCGATTGCCATTCATATTTAGAGC
2541 GTTGACTCTTCATGCGGTTGGTAGCCGTCGGCC
2542 ACCATATGTTGATATGGCCAGCAGGGCTAAAT
2543 AAAGGATAATGAGGCCGAGGTTAATTCTCCGT
2544 CATGTGAAGTGCTATTGAACTGCCGTAACATC
2545 AGCCTACGCAAGTCGAATTAAGCCAGTTAGTT
2546 CGTTCCATACATCACCTCATCTAGTAGTCTTA
2547 TGCATGATGCTTTACCTCGCCGTCGTAGTGAT
2548 TCTATCACCCGACCGTCCACTGTTTCACCAGA
2549 TTTAAATAGATACCAGCCTTAGTGAAGTTATC
2550 GTGCCATGACGGGAGGGTTATCGATACTTGAT
2551 GCAACTGATAATACGGTAACTTCAGCGCCAAA
2552 GCCACATCAGACGTGAAGGGTTTGACATCA
2553 AGTAATTCCACTCCCAAGAGCCGTTTGCGGTT
2554 GACTTTGGTTTCTCGGTCGACCCCACCGCAAT
2555 ATTGCTCTGGCGCAGTAGTTGAGTCACCTGAT
2556 ATTTTAAAGGGGAACCCGATATTGAGGGGAAA
2557 CGTCCCCTTTTTGCAGATCTGCAAATTAAAGT
2558 CAAGCATATAAAGCTGGCCGTGAAAGCATCGA
2559 ACACGCCATGGATTAATCTGACGTTGCTTAAT
2560 GTATGGCGATTCATCGCGAAACAGCAGGTAAT
2561 CTAGATGTCTGATTTTCCCTAAAGATTATTAA
2562 TTGAAGAAGCCATTTACCTTCGTGAATATTAC
2563 AAGTTGGCGATGTTGTTATCACCGCTTATCAA
2564 CCAGCAACTAAAGAAGTTATCGAGTCATATAT
2565 ACAATGCAAGAAATTAGCGTTGATGTTATTGAG
2566 TTACGCGGGAGATATTGACTAGCAGGTAGATT
2567 TCTGATAATATTCGAAAATTAGGCGCTATGAT
2568 ACTGCTTTCATTGTCATCTTATTAATCTCTCT
2569 TTAATGGATGTTGGAAATATGAATCCTAATAA
2570 TTTACGATGCGACTTGACTAAAATGACAATTA
2571 ATACCAATGCTCTTTTTAATATTAGCAGTCA

2572 AGTTGCCGTCCTTATCCCATTTAAACATTCTT
2573 TCTTTCTGCCGTCTTTCCGATCCTTTATCGAT
2574 GGTATGTTCCAGTTCCCACCTGAGGGGACACT
2575 ATTATGATATGAATGTTACAGGCATTGTTATT
2576 TTACGCGGGAGATATTGACTAGCAGGTAGGTT
2577 GCGTAAGGATCTTTATGAGCCAGTATCTTTCC
2578 ACATAGGGGTTTAACTGCCCCTTTACACGGAT
2579 AAGTAAAACCACTCGTAATCACCTAAGCCACT
2580 GTGGTGAGGGATGCATGATAGACGAAGCCGAT
2581 CCGTGATTTGCGGGAGTTCGCCGCCAGCCTGA
2582 TCGGGAATCGTGGCGCTGGCGTTGCTGCTGAA
2583 CTTATCCTGGAATCGTTACAAGCTGATGCGAT
2584 CTGTGGTGGCAGTGATTGTTGTTGTGATTGTC
2585 TCATACACTTCTTATAACCGGCGATTGTGCTT
2586 ATCGCCCGTTGCGTGGCGCAACCGCTTTACTG
2587 GAAGAGCAGATTAAAGCATCCACATTCAATAC
2588 ATTAAAGCAGCCGGTTTCACTTTCGCGGTCGA
2589 GTAATTAAAGAGATTATTCTTACCCGCTTGTAG
2590 TGATTTCATTTCAAACCCGGTCACTTGCACCG
2591 AGTTGCGTGGTTCTGGCCAACCGTGACATTTT
2592 GTGGTAACTGCCGAATATTGTGTTTCCGATAT
2593 TCTTTCTGCCGTCTTTCCGATCCTTTATCTCT
2594 GTCGTGAAGCTAAACTCCGTATCTCATGAACT
2595 GTTATTATCCGTCACAACAGTCTGTTCCTTCT
2596 GTCATGATGGGTTAATCACGTCAATGCACTCT
2597 CCAGCAACTAAAGAAGTTATCGAGTCATATCT
2598 CCAATAAAAATCTTCAACATACTCGCGTATCT
2599 GCCAATCATTTTAGGCTCGACATCTTCCCATT
2600 TTGTAGCTTTCTAGTAACTTGTCTACACAAGT
2601 TATCACGCCAACCGCCGAGGTTTGCTGCTGGT
2602 CAGCGCTGTAACAGGCGTATCCCTGCTTAGAT
2603 CTAACTAAACACATGAATATGAGTTGGGATGA
2604 TACGTTTTCACGTAGCGCAGTATCTGCATCAG
2605 TGACCCCGGCAAGATTTATTACGTATACCGCCC
2606 CAAAGGCCGGCGAACACGAGACCGACTTTCTC
2607 ACGAACGGTGCTCTCACCCGCCCCTATCTGTC
2608 TCGTTGCCCCACAACGCGGGGACCAACTGCAC
2609 CCTGCTGATCACCTTTGAAAAAATCCAAATAA
2610 CGGTGTTCGCATACCACAACAAATCTGTTACC
2611 CCGGTGTAGAGACGCAGAAGACGCCGTATATAT
2612 GAAATGGCGACAGTCACAGACGTTGATGCAAA
2613 GACCTTGCAATGATGCTCGGTTATAGCGGCAT
2614 GTAGCGTTCACCAGATGTATGGTTATGAGTAT
2615 TGCACGAACGATGCGGAAGTGTCTCTCGAATG
2616 TGGGCGGTGAAGCTGTAAATCTACGGTCATTG
2617 TCATCATACGATGATAATCGATATCCTTAGTC
2618 TTCACAGCAGATAAGTTTGATGTTACCGACCA
2619 GAAAAGAGAGACAACCCTCACATTTGCACGGT
2620 GATACCGTACCACCTTCCCAGCGCATATCAGC
2621 GAACTGCTCATCCTCTTACCCCTTAACTATGT
2622 CCAAGCCGGGTGAATGCCTCCCTCTGTGCGAC
2623 TAATAGTGCATGGGAAAGTTACGGCTCCATCTG
2624 AAATTATGGCTATGCACAAGTACGGTGCAGAC
2625 GTGATATATGCCCGATCAAAGATACCGCTAAT
2626 GTGTTGGTTGCCGCTGACCGGTTGCCAGTGTT
2627 AGCTGTTGTAACGAATGCTAGCGTCGTTGATG

2628 TTTTCCATCATGATCGATCGACCACAAGACCC
2629 TGATCGCCTGCATCTTCGACATACTTCTTGAA
2630 AAATCAGCAGTGCGGCGGGTTTCACTGGATTA
2631 GGCTTGCATTCCTCCGTCTGCGGCGTAGAGCA
2632 ACAACCGGGTGATAAGTGTGGTTTCTCCGTTT
2633 AGTTTGCCGCCGTTCATGATGCTGCCGATTGT
2634 GATGGGTTAACCGTTCTTGGTGGGGCTAGTGA
2635 GCTCAACATGCTGTCCTTGCCTGATATCCAGC
2636 ATGTATGCGAATGGTTACGCAGCTCAGTGTGAT
2637 GTCGTGAGAGTGGGTCATAGATACCTCACATT
2638 CATAGAAGAGACAAAAGGCAACTTGCCACAGG
2639 GGCTACACAGGGCCGGGCGGGGAGTGGGAGGA
2640 TGAAGTTGCAAACAGTGCCACTGACCGCATTT
2641 ACTCAGGGGCAATCTGTAGTTAATAGAGGAGA
2642 GCAATGGCGATGTAATGGATTGTTATCACCAT
2643 TGTCTAACGAGGCTGGCTGCGCGACTAAGCCT
2644 CGAAGCCGGCATCACCGATCGCGTGGATACACG
2645 GGGTGAGCAAGGCACGATTGTTCCAGGATTGT
2646 AATTTGCAGATCTGCAAAAAGGGGACGCTGTA
2647 TGCTAGCGACGCTCACTCACGTAGTGACTGGA
2648 TAATGCCGATGAATACCGGTCGAGACGTGTTT
2649 GCAGGCCGAAGAACGCCCCCGCGATATCCTCA
2650 GTTTAAGGCGGGAGGGGCTAGGCGGCTTTCTT
2651 GACAACCGTACCTGTGCTTCGTTTTCACTCAA
2652 GATGCAGGGGCATAAAAATGACACAGCGAATT
2653 TTTCGTTAATCTCTGCGTATTCTTTTGCTGTC
2654 AAGCGACCGCTCGAATGCTGGAGCGGGTTTCC
2655 GCCTGAGCTGTCTGGATAGCCAAATCCATTGC
2656 CAAATACAGCAACAGGCACTATTGATTTCACT
2657 GCATACCCTACCTGCTTAATGCCCTGTACCGT
2658 GATGGCAAGAGATACATGCTTGATTATCTCGT
2659 GCAGAGCGTGATCGTGTTGCATTGGCTCTTAA
2660 TTATGCCACCACTTAAGTTCCTTTTTTTTCAT
2661 GATATGAAGGACTTAAGCTGATGTCAGTTCTT
2662 TCCAAACCCATACCGCTAATCATGCTGCTAAT
2663 AGCGGCGCTGCTTTGAGATCAATGATAACATT
2664 ACTGCCATAGGGAGCCGAATACATACCTAGCT
2665 GCCAATGCCCACACTTTGTGGGCTTTTTTTAT
2666 ATCCCAGCGAGACTGGCCAGCAGTCCCATGTC
2667 CAGGTGATCCCACTTGCAGAGGTTGCCGCCAA
2668 GAGAGAAATGGTGGTCAGCGCTATTACAGCAT
2669 GTTACTTGCAGCCTCCCGAAGCCCTTTGACTAA
2670 TAGCTTTAATCGGTATGGCAACATTTATGAGT
2671 TCACATATCTTGACTGGATCCTGCTTGGAATC
2672 ATCGACTGTATACGCATCACTGAAAATTCCTGG
2673 GGTGATGACGTGCTTTGCAATTGCCAGAGCAA
2674 ATTCAAATACTGGAAACCATCCTTCAAGTAACG
2675 CTATGTTATTGCGGAATAGAAACTCACTGATT
2676 CATTGGCGGCACGGCAAACGCTCAGGATGTTA
2677 ATAATAAGCAAGGGATTGTTGAGAAGCTCAATGA
2678 TACAGGGCTTTATTAATGGGTGTCGGGTCACT
2679 TGTAATGACGATGACGGAATGTTGATTTCTCA
2680 GCACAAAAAACCCGGCAGCGTGGCCGGGTTAGG
2681 AATATCAGGAATTTATGGCAGCGAAAGCCAGC
2682 TGGCTGCTCATCTTCTATTTTTGACTTTGTAT
2683 ATCTGGACGCTGTTACTTGCTACAGAATACTT

2684 TTTGCGCGGGAGGCTGGCTGGGCCGAATCAAA
2685 ATGATCTATCTATACCATCTATCAGTTACTCA
2686 GTCGCGCTGCCGCCAGTGTAATCAAACATTGT
2687 AATAGATTGCGTGATAGTAATTGACCGTCGGCT
2688 GCGTGGCCTATTTAAGCATGAGCATTTACGGA
2689 GGCAATAGACACACCAACATCCGCCACGCAGA
2690 AAATATAAAGATAAAGTACCAACCCCTAAAGTG
2691 CTTGACAACCGAATCGGGCAGGGTGGCCCAGT
2692 CGTTAGCGTTTGGCGCTTACGAGTACCTTGAC
2693 CGATTTGCATTGACGCCAGTCGATATCCCGCC
2694 ACGAAGTGGACTTACGGCCGCACGGGCGCGAT
2695 ATATCATCGGCGCTCAGGGACTTTGTACAATT
2696 ATATTGATGTCAGGTATCCTCCGGGATTCGTC
2697 CGTCGGCTCGCCGAACATCAGACAGCGAGTCT
2698 CATTCCGCGCTCGTCACGCGCCCTTACAGCAA
2699 ACATTAACCTGATTTGGGCGCTTGCTGATTCT
2700 CTGTCGTATTCGGTAGTCCAGGAACGGTAACA
2701 AATATTGGGAGTCTATTGGTGGTACTTGGGGA
2702 TGTTGCGAAACAAACCTGAATATCGAACGTCAG
2703 CATATAAACATGCAGGGGCGTTATTCCTTCAC
2704 GGCACACAGCCTACCCGTGCCGAACTGCTGCT
2705 TGCTCATATTGTTTACTCTGTTGGGTTTTCTC
2706 TCAGGTAAGCCATGAAAGAGCCAAGGGTATAC
2707 GAGTTGCTGACAGTTATTGAGTTGTGCAATCT
2708 TAAAAACCTTTCTTCAAATTCTTTCCTATATTC
2709 GCTAGAAATTTAAGAACACATGTACCCTATCA
2710 ATCTAGTGCTCCTTTTTGGTCTATATCCAGCG
2711 TATATTTATAGAAATATCCAAGTTTATCATGAT
2712 GTTTACTGCTAGCAAGATAGCAAATACATCAT
2713 ATGCCGGGATGGTTATTGGGCTTGAAACATCT
2714 CTAGAAACAACGGGATATCTAGTGCTCCTTTT
2715 TGGAGGGCTGGGTGGTCTGCGCATTCCGATAA
2716 CATTTGTGCTGGGCGGGAATTACCGCAGATTT
2717 CCTCGCGGGGCTTCATATTAAGCAGTAACTGT
2718 AAAGCCAATGACCGTGACCACCCTAACGATGC
2719 AGTTTCACTCCCCCACCACGGAAAGGTATCTG
2720 TTAATTTTAATCAACTCCTGAAATTCAGTCAT
2721 AGAAAAATATGGCATTGCATTGCGCATATTCG
2722 TTGGTTAAATTTTCATTTGGGTCAATAAGCAA
2723 ACATCTTCCCCGTTTGGGTTCGCCAGACCATC
2724 TACGATACAATTAATCTCGGTCAATGTCTTGT
2725 TTCCTCATGAGCGCGTGAACTGGGTGGAGACA
2726 GGTTATGCATATCTATTGTTCACGCTTTAAAT
2727 GTAATGGCGGGTTTGGTGGTTTCGGTAACCGT
2728 AATTTGGACGCAGACACGATGAAGTGTCCTTG
2729 ATACATAAAACCAGATAATGCGCCGCCAGACT
2730 GAGGCTTTTTATTAAGGATTGAATATGGAATT
2731 AAAATAAAGTAATAGAAGTCATTAGCGTGTCT
2732 TCATTTCTACCTTTTGATTCTGAGGGATATGA
2733 TCAAAGCCATCATCATGCCCTTCGTTGTCGCT
2734 GTTATACCCCGCGCAAGGAGTGAAGCGTTGAC
2735 TATCTGAGATAGAATTTCATGATTCAGATTTA
2736 ATTCTGGATAGGACAAATAGGATGATTGTATC
2737 TTCATGGTCTCCCCCTCCCGGTCAGCGGGTTG
2738 ACTTGAATCATAGCCGTTAGCGCATCTTTAAC
2739 AATATTGGGAGTCTATTGGTGGTACTTGGGGT

2740 AGAAATGAGTAACGTTCAATTCCCGCCCCGTGT
2741 TTACGCGGGAGATATTGACTAGCAGATAGATT
2742 GGCTAGCCTCGAATATGGCGATGTCACTATGT
2743 GGTACAAAAAATATGCCCTTTGGTTTATGACCG
2744 CCTCCCGGTCAGCGGGTTGGGCACGTAGTTAA
2745 ACAATATTATCTCATTATCAGACTATAACGCA
2746 TTGATATTACCTAGCTCCCGCCAACCTGTTGTT
2747 ATCTCCGGAGAGTTCAGCTGGGAGGTGGTGTA
2748 ATCTTCAACCCAACACTCAACCATCGCGGTAG
2749 AGGAAGGCCAGTGTGTACATCAAGTCAACCTT
2750 TCAACCGGCTTAATGCTTAATCGTATTGATGA
2751 ATCATTTGCATAACTGAACGCTCCATCATCTA
2752 CATTGGTTACTAAGTCATAGAATATCCATGCA
2753 ATTGACATTGTGCCCGATTTTCACATTGTCACG
2754 GCCTTGCGGTCACTGATGATCCCGCCAGTCTG
2755 ATTGTCACGAAGCGGCCAAGTACCTACCAGAA
2756 ATCTAGTGCTCCTTTTGGGTCTATATCCAACCG
2757 ACGTAAAGTTAGCAAATTACATTTTATCCGAG
2758 CTGCTTGAGCAATAAACATCGCCTGCTGTACA
2759 TCACTTGATCCTCTGATGGTCGATATCCAGCG
2760 ATTATTGAGTTGCAAACTACTAGCAGCACAAA
2761 CTGACGCTAGCAAGGCCAAGGGTGATTTTGGT
2762 AAGCAGGATTTTCTATTACTACTGCATTATTT
2763 ATGGATGCTTGTTGGTATTATTTAATTCAGGA
2764 GATTGCATTCTCTTTCGCCACTGTCTCCATGT
2765 ATTCAATGAAAGAGTTGGGGTTTTCAACAGAA
2766 CCAGTAGGCGTTATCGGTCTGGTATTGATGGA
2767 ATGGAATAATTGTCATGACGTCGGATGGTGAG
2768 GGCGTGTTTCATCAATATATCTCCATCACACT
2769 CAGATTGCCAGCCGATCATTCACTTCATTGAT
2770 TAGTTACCCTGCTTCTCCTTCTTGATTGATTC
2771 AGTCTTCCATGTCTTCATCAAGTTTTGGCTCG
2772 ATGTGATTCCGGTCATCGATAATGCCCACTTA
2773 ACTTCTAAAAATTCAATGTAGGAGCTTACATA
2774 GTATTCCTCCATTCTGTTAAGACTTGGGAGAC
2775 ATATTGAAGTCAAGCGCTCAGGTGAACGAGGT
2776 ATAATTAATTGTTGTGCTTCATCCAACGTGTT
2777 CCCGACTCACCGCCGCCCTGATCCATCCCCTA
2778 AACAGCAAGTCTACGGCCTGCCGGATTACATT
2779 CCTGCGCCCGGTTGATGGCCGGTGAATCTCTT
2780 AATAGCGATGAGGTTAATAAATCTGATTTAAT
2781 ATAATTAACTGTTGTGATTCATCCAACGTGTC
2782 TTATAGTGATGTTAAAACAATTACTTCAATGA
2783 TATTAACTGGAGGTAACACCATGGCTCATCAA
2784 TCAATTATTAATTATAGTGTTATACTTATTTG
2785 GTTCCTGCTGGGTGTCAGTGGCGAACTCGGGC
2786 GACTTGGATGTTCCCGCCGACAGAATGACCGA
2787 TTAAGCCCAGCACCGAGAGCATCCCTAACGTT
2788 CATCCGATCCCCGCCTCTCGTTGACATCTTGC
2789 ATGACATGATAGATATTCAACATCTGGCGCAA
2790 AAATCAGTGGCGGTAAATATATAACTGACGAA
2791 GGAGCCAGATTGTGGCCGCTGCCGGCGGACTCG
2792 ATCGTAGGCAGCAGGATGCCTAGGTTCTTCTG
2793 GCACTTTTTGTTAATAAAGCATGGATGCTTAT
2794 TATTGGCTTCACGATACCTCGATGTACTTCAA
2795 GCACAGACATAACCGTCTATGCGGTACCAATT

2796 CTTTGTTACTTGTGCCGGATTTACTGACATGT
2797 ACAACCCGCTATCAGGATATTGCTGACCATTT
2798 GTTAATCAGCATGTAACCCACGAATTCTACCG
2799 AAGCGCTTATCACAATATCTGAGTTCGGTATG
2800 TTCCTTTTGAATGAGCATTCAACCTCATCTTC
2801 TCTTCCTTCTCTTATTTCTTTCTCAACCACAT
2802 ACCACGGCCCAGTACGGACAAGTAGCGACCGT
2803 TGGGCGCATGGTCTACCGGATTCGCTATTGCA
2804 GCGAAAAGCGTGACCACTTTGCTGTGCAATTG
2805 GCCGGGTTTTTTGTGCCTGTAATCTGGCAGTG
2806 ATATACAGAGAACGGCAGGAAGCGGGTTGTTC
2807 TGGGCACGGTCGGTATTTACATTATGTGTGAT
2808 GCGAGGCATAAATGTTAAACCTTGATACGTTA
2809 AGGAGGTTGTGGATAAATCGCGTAATGAGATGG
2810 ACTTGATAGTGATAATCAATCGGATACAGATT
2811 GAACGTTTCGCAGCCATCGACCAACGTCTGCA
2812 GTGAAGCGCTACCGGTTACACAATGCTTTTAA
2813 CGGGCTACGCTCTGCAATCAATAACTTGACCA
2814 CCAAATTCATCGCACGTCTCCTGCTGTGTACT
2815 TTAGCGGTATGGGTTTGGACAGCAATGATACC
2816 AGCTCAATCCAGAATGGGGATGCTATCTTATG
2817 AAAGATATCCTGCAGACGCACGGTATGCTGAT
2818 AACTTTCCAGAACCCCCGCGAACAGGGGCAAT
2819 GCTTTCTCGCTCCTCAAACCGATATTTAGCTG
2820 GCGGTGATGGTAAAAACTCATTTGGCGAAATA
2821 GATAACACCGAAATCAACTGCTGACAGCTCGA
2822 GTTTCACATTTAAAGAGGCTCAATCTTCAACT
2823 TGTCAGTGATTGGCGATGAGCGGGATTACAGC
2824 CTATCGACCCTGGCGAAATTGCTGACGCGCTT
2825 AGATAGGGTTTTCCGGTGCCGCGAAAATTCAG
2826 TCATCTTCATCACAAGCCGGTTTTTACTTTCC
2827 CATCTTCATGACCAGACGGTTTTTGCTTTCCT
2828 ATATCTCAAACGAGGATTATCACTCGGGGCCA
2829 TAAATTCAAACCTTGTCAGTCCCATTACGCCG
2830 TTGAGACTAATGACAAAGTTCCCCGCCGAGTA
2831 TTTGCGAGCTTTCGAAAGTGTGTTTTCAGACT
2832 ACGAGGCAGTACCCAGCCGAATGTGGTGTAGC
2833 GTTAGCCGCCATTCTGAATTGATGAGGGGTCAT
2834 ATTAGCTACGACCGCGTGACGAATCGTGCCAC
2835 TCATCAAGACGCAAGCGGTAAAATGGCGTGAT
2836 GTGGGTTTCGACCTCCCGCGCTGGTTTATTCC
2837 AGTTATATGAGCCGGATATTTGATGTTATTCA
2838 GCTACCGGGCGCGGTGATTTAGCGGTCTCTCT
2839 CGGTTATTTCTTGAGCCAATCTTTCTTTGTAT
2840 ATTATCGAGGCGTTGGGGCACATGCTAGACGC
2841 CAGAAGCGATTTTGTAGTTTCATTTCCCTACA
2842 AATAGACGGCTATAAGTTACAAATGCATTTTT
2843 ATTGGATTTGAGCCAGTTCACTTATGGAGTCA
2844 GATATCTTGACGTAATCCATTTGTGCCGGAAT
2845 ATCAGCCGCTTTCTTTTCAGATTGCCGCTTTT
2846 AAAAAGCTTACTGCGTAGTTTCGAGACTAAAG
2847 CGTGGGCAGATGATAACCGGGCGTCCAACACT
2848 TAATCGTGTTTTATACCCATATTAAACCTTAC
2849 TTAGCCAGCCAGCCGTTCATAAATGGCTGAAT
2850 TAAAGATTAACGGAAAATTGAGTTCATATGAA
2851 TTTTCTTCGTTCCTCAGAAAATACTCTCTTTG

2852 GTGTCATAGCAGGCCAAGGCTTGAACTATGAA
2853 GACCCGAAAGACATTGCCGACATCATTAGCAA
2854 CCGTTAAATCCGCCAGTTGAACCAGAGCCACC
2855 CAACCATACCCCCGCCGCCCCCACCTGAAATA
2856 AATGAAAATAGCACTAGATTATATTTCGTTGT
2857 TTTTGGGGTCGTGCCTATCATCACCTTGATAA
2858 GAATACCAGACGTGTTATTAGCTATTACACTAG
2859 ATAATTAATTGTTGTGCTTCATCCAACGTTCC
2860 ATTGGTGAGACCATCAGCAGGAATGCTACACA
2861 AAAAAGCACCGTATAATCATCCGAAGGTTGAC
2862 CTTGCCCGTTACCACTAACCATTATTTCAATT
2863 GAAAACGTTCTACAGTTTATTGAGTCTGTTTA
2864 TTTTAATATTATTAATTTCATCCCATTGCTGCT
2865 ATTATCAGCATGATAATGGCGTGTATGTCGAT
2866 AAGTCAAGTATTATTTCCACTTATAATAAATT
2867 TGAATTAATTACTTTAATGCCTAGGTACTTGA
2868 GATTCATATAGAATATGGTTTCGCCTGTGTCT
2869 ATGATGATCACAGCATCATCATTAAGCGGCAAC
2870 AGTGTATCGCTAACTCGATTTAACCCCACAAC
2871 CAACTGATGGATACGTTTCTCATGCTCCCAAG
2872 TGGTTATGCATATCTATTGCTCACGTTTTAAA
2873 CTTGTCCGTTGCCAGTAACCATTATTTCAATTG
2874 ATACTTTGTTTGAAGAAAAATTAGAAAATCAT
2875 CCCCTCAGAGTGTAGTGAGTCAAACATAGAGG
2876 TAAACCATTTCCTTGAACATGTATTTCAACTG
2877 ATTTTATCTGAGAGTGATGTTCGCGAACTAATT
2878 GCGCTTACGCTCACTCGCAGTCGATGGCATAC
2879 ATCGGAACCCCTCGACGTTACTATCCGACATA
2880 GCCCATGAAGCCCCACCCATTCCAATTAAATAA
2881 TAAACGCATTAATTTAGACTCACAGGCATTTTT
2882 CATTATATGCGTTAAAAGCTGACACGGTGCAA
2883 CTGTTACCCATATTTATAAGCTGATTCATATA
2884 TTTTAATATTATTAATTTCATCCCATTGCTGTTC
2885 AAGAGCCGCCTTCTTGTCTTTCGCGCGTTGTT
2886 ACTTATTTCATCGTCAACATACTGTTTAACTT
2887 TTGAGTTACAATGAAAAATGAAGCGTCTAGCG
2888 ATTTAAGTCGAGCGTGTGAAGTTTATGCAAAT
2889 TCTTGTGGAAAGAACACACGTTACTGATGATT
2890 ATTTCGGTGTTGACGCGTGCTGGTGTCATGGA
2891 TCCTCAGATATTAAAGATATATAAACATTAGT
2892 AATAGCTAATAATACTTCTGGGATTCCAGATA
2893 ATTTGATTGTGCTGGCCCTGCGCGATGCGGTAG
2894 TTTTGGGGTCGTGTCTATCCTCACCCTGATAA
2895 GCGCTTACGCTCACTCGCAGTCGATGGCATCC
2896 ATCGGAACCCCTCGACGTTACTAAAATTGTTA
2897 GAATGGAGATTATTTCGAAACTTGGTGATGGC
2898 ACTTGCATCCCTGCAACTCTTCACATCCAGCG
2899 AGTTTGTTTATCAATTATTTCAACCATACTTT
2900 CTTGACCGTTACCAGTAACCATTATTTCAATT
2901 ATATGATCCCCTTTATACCTCTATTAATGGGT
2902 TTTAACTAAATCTGAAGATTTTAAATTGTTAG
2903 GTTTCACATTTAAAGAGGCACAGTCTTCAACA
2904 ACCGCCTTTACTCCGCTTTCTCTTATTGTACT
2905 CTCAGGTGCGGCTGCGATGAGTTTGGCATCAT
2906 TATCCGGCCAATGAGATTGCCGAATCTTACAT
2907 TGGAGACAACAAAGTCGTTGAAATCTCATACT

2908 ACCGGCCTGACCGCGCAGAACTCCGCCGCTCA
2909 TGCCAATACGAGCCTCAGGGCTACGTCTATCC
2910 AATGGCACATGAGTAGAACTAATATCCCAGCAG
2911 TGTCTACTGGCTTCTTTTCTTCTTCGCGCTTTG
2912 ATATAATATCTCTGATGTGTACGCTTCCATTAT
2913 TATTCTGCATCGGCCTAGCTGGTTCTGGCTCT
2914 ATCATTCATTAAGTCCTGGCCCTCGTCACTGC
2915 GTTTAAAGATAATGGCTCGATTATTCGTCCTT
2916 CATCACAATGCTCTGGATGACGCAATTCATCA
2917 TTNTATTCTTACTAAAGATATCTACTCAGATAAT
2918 CATCACCATTCAGTGAGTTAACAACTGACTCT
2919 GCGAGCAGCATCAAAGCCCTGAGAATTGCTAC
2920 TTACGCGGGAGATATTGACTAGCAGGTAGATG
2921 TTGACGTCACGCTGTACTACACAAAATCAATT
2922 TGATGGGGCAGGGTAAGTTTGCTGCTCAGATG
2923 GGGATCAGCACGCCGCCTTGCTTCATGATGTA
2924 AGATAGCACTCATTAATACCATTTCATTAATT
2925 GATCACTTATCTCAGTGGGAAAATCAGCTTTC
2926 TCACTTGAGCCTCTGATGGTCTATATCCAGCG
2927 TGGAAGGATTCGAATATAACGAGTTGTGGAGT
2928 CACTACAGCCATGATTTTCTGGCTAAAGAATA
2929 ATGCAGCACACGGAACGGCTCACCGTCCGCCT
2930 CAAGGGCCGCTTTGTTACTTGTTCTAGCAGTT
2931 AACTCTCCGGAGATACCGGAAGGTTGGAAGCT
2932 AGAGTAAACGACTCAGATGCAGTTTTTGCATA
2933 TTGACGTCACGCTGTACTACACAAAATCAACG
2934 GCTTTTTTGTGCAGCAAGTTTTGATTTCACGC
2935 TTGTCGGTACCGGTGTCAGCATGTCTCACTCA
2936 GGGCAGGTAACATACAATTGGATTTCCCAATT
2937 GCAGCAACATCTCTCAGCAGCTAACTGGGCTT
2938 CGTGGCCCTCGAATAAAGGTCAGCTCTCAGAT
2939 GGTTCATATAAAATATGGTTTCGCCTGTATTG
2940 TCTTCAGTACTCACGCCCTGAATGTAGAAAAC
2941 CTCTGTGACGGTCGCAGCCTTGAAGTTAAGCC
2942 ACTGATGGCCATATTGCCCGTATCAGAAGTAT
2943 CATCCATCATGTCAAATCGCTCACGACACTTA
2944 TTTGCGACATCAAGAATCGAACAAGTGGGCGA
2945 TCAAATTCATGGAAAGGAACATCTTGGCTTCAT
2946 CTATTGGCTCGGTAAAATGGCATGTAGGCGTT
2947 CTGAATGCCTCGATTTTATTGAAGCATCTGGC
2948 TAGCAATCAGTCAGAATGGCGCTGTCATTGCC
2949 GTTTTCTTGAGTGACAGACTGGCAGTGTTATC
2950 CACGGGCCTGCAAGTAAAGAAAGCTGTAGGCT
2951 GTCAATCTCATCAGTGGAACGTACTGTTATTAT
2952 AATTGAAATACCACTTATCCCGCCCGGCCTTGG
2953 CTGGAGCGGCAAGGCGCTACCGAGTGGATTAA
2954 AATCAATTGGTGCCGTAGTTCTTAAGGGTGAT
2955 AACTAAGGACTCTCATGAGCATTAAAATTTCTG
2956 TGAATTTTGACGATGCTGGCTGTTTGGCTTTT
2957 TTGCGTACTGCGGATTCTTCCACCAAGAGAAG
2958 ATCAGGAAACACGTTTCAGTTGGACAGCTACA
2959 CAGGTGCAACAGGGACTTTAGGATAGAAAAG
2960 AACGAACCCACGTAGAATTGCCATCACCGCCGGG
2961 GCCTAGGCAGTCAGAGGCGATGAAGGGCGTGC
2962 AACAACGTACAACTCATCGTCAATAATCATTA
2963 TGTGGAATATGGCTAAGTCCAACTGAATGATG

2964 AGAGATGATAACCATCATGATTATTAGCATGA
2965 ACCTCCATCGGTCGCTCGGGAGTTTTCATGTG
2966 GCTGATACGTGGCGGCTTATTCAGCCCAATTA
2967 ATCAGTGGATTAACTGAAGAGTGGCGCGACTGG
2968 GTTATAACCAGCGCTTTTCTATGCCATATATT
2969 CTGGCGCGAGTACCTTCGTCCATTTCATCAATT

Table S3. CRISPR spacer pattern based clustering of *Y. pseudotuberculosis* strains. The clustering was carried out as described in the article main text, Materials and Methods. The most ancient spacers in the columns are at left side, and the most recent spacers, *i.e.*, the leader proximal ones, at right. Multilocus sequence type (ST) according to MLST Databases at (http://mlst.warwick.ac.uk/mlst/dbs/Ypseudotuberculosis, see Laukkanen-Ninios et al., Env Microbiol. 13: 3114-3127, 2011). Ns, no sequence information available. The *Y. similis* and *Y. wautersii* strains were excluded from the clustering.

Cluster	Strain name	Species	ST	O-Serotype	Host group	Host	Year	Continent	Country	Location	YP1	YP2	YP3
[illegible]	[illegible]	[illegible]	[illegible]	[illegible]	[illegible]	[illegible]	[illegible]	[illegible]	[illegible]	[illegible]	[illegible]	[illegible]	[illegible]

22	4360	Yptb		1b	Mammal	Human	2000	Europe	Sweden		[illegible]		[illegible]
22	2813/1986	Yptb		1b	Bird	Pigeon	1986	Europe	Finland		[illegible]	[illegible]	[illegible]
23	K22	Yptb	40	5a	Mammal	human		Asia	Japan		[illegible]	[illegible]	[illegible]
23	CK0428	Yptb	72	5a	Mammal	monkey	1988	Asia	Japan		[illegible]	[illegible]	[illegible]
24	YT14	Yptb	14		Bird	red-winged blackbird		North America	Usa		[illegible]	[illegible]	[illegible]
24	IP32683	Yptb	14	12	Mammal	human		Europe	France		[illegible]	[illegible]	[illegible]
24	H305-36/80	Yptb	43	1b	Mammal	deer	1980	Oceania	Australia		[illegible]	[illegible]	[illegible]
25	PC84-72	Yptb	44	4a	Mammal	pig		Asia	Japan		[illegible]	[illegible]	[illegible]
25	D1040	Yptb	44	2b	Mammal	dog		Asia	Japan	Shimane	[illegible]	[illegible]	[illegible]
25	PC004	Yptb	44	2c	Mammal	pig		Asia	Japan	Shimane	[illegible]	[illegible]	[illegible]
26	PST1813	Yptb	14	4							[illegible]	[illegible]	[illegible]
26	H1486/84K	Yptb	14	3	Mammal	human	1984	Europe	Germany		[illegible]	[illegible]	[illegible]
27	SP-940813	Yptb	8	15	Mammal	human	1994	Asia	Korea		[illegible]	[illegible]	[illegible]
27	WP-931108	Yptb	6	15	Mammal	water	1993	Asia	Korea		[illegible]	[illegible]	[illegible]
28	1430/8/2004	Yptb		1b	Environm	Carrot	2004	Europe	Finland	Haapavesi	[illegible]	[illegible]	[illegible]
28	2181/10/2006	Yptb		1b	Environm	Dirt sample from storage room floor	2006	Europe	Finland	Hämä	[illegible]	[illegible]	[illegible]
28	283	Yptb	14	1b	Mammal	human		North America	Canada		[illegible]	[illegible]	[illegible]
28	2874/2003	Yptb		1b	Environm	Kitchen bench surface	2003	Europe	Finland	Kotka	[illegible]	[illegible]	[illegible]
28	2484/2006	Yptb		1b	Environm	Potato waste on storage room floor	2006	Europe	Finland	Loppi	[illegible]	[illegible]	[illegible]
28	2823/10/2004	Yptb		1b	Mammal	Straw	2004	Europe	Finland	Haapavesi	[illegible]	[illegible]	[illegible]
29	PC708	Yptb	3	1b	Mammal	pig		Asia	Japan		[illegible]	[illegible]	[illegible]
29	DD382	Yptb		1b	Mammal	dog		Asia	Japan		[illegible]	[illegible]	[illegible]
29	T-489-1	Yptb		1b	Mammal	pig		Asia	Japan	Tottori	[illegible]	[illegible]	[illegible]
29	TR-83181	Yptb	3	1b	Mammal	Racoon dog		Asia	Japan	Tottori	[illegible]	[illegible]	[illegible]
30	301 Kope	Yptb	48	4a	Mammal	horse		Europe	Denmark		[illegible]	[illegible]	[illegible]
30	H-3831	Yptb	48	4a	Mammal	human	1980	Asia	Russia		[illegible]	[illegible]	[illegible]
31	J81	Yptb	47	13	Mammal	rabbit		Asia	China		[illegible]	[illegible]	[illegible]
31	R103-2	Ysim	45	5b	Mammal	Rabbit		Asia	China		[illegible]	[illegible]	[illegible]
32	IP32684	Yptb	41	12	Bird	bird		Europe	France		[illegible]	[illegible]	[illegible]
32	2815	Yptb	7	27							[illegible]	[illegible]	[illegible]
	IP32463	Yptb	18	5	mammal	guinea pig		Europe	Switzerland		[illegible]	[illegible]	[illegible]
	IP32881	Yptb	18	3	mammal	monkey		Europe	Switzerland		[illegible]	[illegible]	[illegible]
	IP32831	Yptb	18	3	mammal	hare		Europe	France		[illegible]	[illegible]	[illegible]
34	H73888	Yptb	2	1b	Mammal	human	1988	Europe	Germany		[illegible]	[illegible]	[illegible]
34	YT32	Yptb	26				1981				[illegible]	[illegible]	[illegible]
	IP33177	Yptb	26	1	environment	cabbage		Asia	Russia	Siberia	[illegible]	[illegible]	[illegible]
34	MW112	Yptb	5	7	Environm	water	1990	Asia	Japan		[illegible]	[illegible]	[illegible]
	IP32544	Yptb	19	3	mammal	pig		Africa	South Africa		[illegible]	[illegible]	[illegible]
	IP32938	Yptb	19	3	mammal	cattle		South America	Argentina		[illegible]	[illegible]	[illegible]
	IP33380	Yptb	32	3	mammal	human		Asia	Russia	Siberia	[illegible]	[illegible]	[illegible]
	YT18	Yptb	19	1a	Mammal	Rat		North America	Usa		[illegible]	[illegible]	[illegible]
	YT22	Yptb	19	1	Mammal	human	1988	Europe	Germany		[illegible]	[illegible]	[illegible]
	[illegible]	Yptb	1	15	Mammal	human	1993	Asia	Korea	Seoul	[illegible]	[illegible]	[illegible]
	PT892	Yptb	52	2b	mammal	pig	1987	Asia	Japan	Shimane	[illegible]	[illegible]	[illegible]
	No.5	Yptb	54	2b	mammal	goat		Oceania	New Zealand		[illegible]	[illegible]	[illegible]
35	CR565-0010	Yptb									[illegible]	[illegible]	[illegible]
35	H3960003	Yptb									[illegible]	[illegible]	[illegible]
35	YT18	Yptb	19	1a	Mammal	Rat		North America	Usa		[illegible]	[illegible]	[illegible]
35	YT22	Yptb	19	1	Mammal	human	1988	Europe	Germany		[illegible]	[illegible]	[illegible]
35	2884	Yptb	19	3							[illegible]	[illegible]	[illegible]
35	BF-1	Yptb		1a	Mammal	buffalo		South America	Brazil		[illegible]	[illegible]	[illegible]
35	G4890	Yptb	19	3	Mammal	cattle	1988	Oceania	Australia		[illegible]	[illegible]	[illegible]
35	Vlessel	Yptb	57	3	Mammal	human		Europe	Belgium		[illegible]	[illegible]	[illegible]
35	IP32802	Yptb	19	3	Mammal	Pig		Europe	Italy		[illegible]	[illegible]	[illegible]
35	2034/A/2006	Yptb		1b	Mammal	Pig stool	2006	Europe	Finland		[illegible]	[illegible]	[illegible]
36	C71	Yptb	67	4b	Mammal	Cat		Asia	Japan	Shimane	[illegible]	[illegible]	[illegible]
36	Pa6726	Yptb	60	6	Mammal	human	1984	Asia	Japan		[illegible]	[illegible]	[illegible]
36	No.151	Yptb	35	6	Mammal	mouse		Asia	Japan		[illegible]	[illegible]	[illegible]
36	Toyama65	Yptb	37	5a	Mammal	pig	1987	Asia	Japan		[illegible]	[illegible]	[illegible]
36	CK10700	Yptb	65	4a	Mammal	racoon dog	1990	Asia	Japan		[illegible]	[illegible]	[illegible]
37	R30	Yptb	3	4a	Mammal	mouse	1988	Asia	Japan		[illegible]	[illegible]	[illegible]
37	87310	Yptb	30	2b	Bird	bird					[illegible]	[illegible]	[illegible]
37	D54	Yptb	4	5a	Mammal	dog	1981	Asia	Japan		[illegible]	[illegible]	[illegible]
37	R30-2	Yptb	36	5a	Mammal	mouse	1988	Asia	Japan		[illegible]	[illegible]	[illegible]
37	PT892	Yptb	52	2b	mammal	pig	1987	Asia	Japan	Shimane	[illegible]	[illegible]	[illegible]
37	R220	Yptb	31	1b	Mammal	racoon dog		Asia	Japan		[illegible]	[illegible]	[illegible]
37	Wa382	Yptb		1b	Mammal	racoon dog		Asia	Japan		[illegible]	[illegible]	[illegible]
37	MW Tarigun	Yptb		1b	Environm	well water		Asia	Japan	Shimane	[illegible]	[illegible]	[illegible]
38	PST26	Yptb	43								[illegible]	[illegible]	[illegible]
38	IP33290	Yptb		1				Europe	France?		[illegible]	[illegible]	[illegible]
38	3808/2000	Yptb		1b	Mammal	Brown hare	2000	Europe	Finland		[illegible]	[illegible]	[illegible]
38	2887	Yptb	43	1b	Mammal	hare	?	Europe	Italy		[illegible]	[illegible]	[illegible]
38	H308-36/80	Yptb	43	1b	Mammal	hare	1980	Europe	Germany		[illegible]	[illegible]	[illegible]
38	3487	Yptb	43	1b	Mammal	Hare		Europe	Italy		[illegible]	[illegible]	[illegible]
38	2613/79	Yptb		1b	Mammal	Human	1979	Europe	Finland	Turku	[illegible]	[illegible]	[illegible]
38	Tyligal	Yptb	43	1b	Mammal	Human		Europe	Belgium		[illegible]	[illegible]	[illegible]
38	Y.P7/7	Yptb	43	1b	Mammal	Human		Europe	Belgium		[illegible]	[illegible]	[illegible]
38	885/81	Yptb		1b	Mammal	Human	1981	Europe	Finland	Oulu	[illegible]	[illegible]	[illegible]
38	3683	Yptb		1b	Mammal	Human	1984	Europe	Finland	Oulu	[illegible]	[illegible]	[illegible]
38	0D7772	Yptb	43	1b	Bird	pet bird	1977	Europe	Denmark		[illegible]	[illegible]	[illegible]
38	0796/K2/1	Yptb	43	1b	Bird	pet bird	1982	Europe	Denmark		[illegible]	[illegible]	[illegible]
38	8357L	Yptb	43	1b	Bird	pet bird		Europe	Denmark		[illegible]	[illegible]	[illegible]
39	H883-36/87	Yptb	12	1a	Mammal	human	1987	Europe	Italy		[illegible]	[illegible]	[illegible]
40	MW145-2	Yptb	50	1b	Environm	water	1991	Asia	Japan		[illegible]	[illegible]	[illegible]
40	Wa738	Yptb	33	1b	Bird	duck		Asia	Japan		[illegible]	[illegible]	[illegible]
40	Pa3606	Yptb	2	1b	Mammal	human	?	Asia	Japan		[illegible]	[illegible]	[illegible]
40	Pa3587	Yptb		1b	Mammal	Human		Asia	Japan	Shimane	[illegible]	[illegible]	[illegible]
40	MW145-2	Yptb	50	1b	Environm	ml. water		Asia	Japan	Shimane	[illegible]	[illegible]	[illegible]
41	H14184	Yptb	8		Mammal	human	1984	Europe	Germany		[illegible]	[illegible]	[illegible]
41	Y248	Yptb	26		Mammal	Hare	1988	Europe	Germany		[illegible]	[illegible]	[illegible]
41	YT17	Yptb	8	1a	Mammal	human	1984	Europe	Germany		[illegible]	[illegible]	[illegible]
41	YT30	Yptb	42	1a	Mammal	Mouse					[illegible]	[illegible]	[illegible]
41	YT6	Yptb	14								[illegible]	[illegible]	[illegible]
41	Ypa717	Yptb									[illegible]	[illegible]	[illegible]
41	Pa3422	Yptb	15	4b	Mammal	human	1982	Asia	Japan		[illegible]	[illegible]	[illegible]
41	CN3	Yptb	17	14	Mammal	rat		Asia	China		[illegible]	[illegible]	[illegible]
41	80422	Yptb	1	15	Mammal	human		Asia	Korea		[illegible]	[illegible]	[illegible]
41	Pa3423	Yptb	61	3	Mammal	human	1982	Asia	Japan		[illegible]	[illegible]	[illegible]
41	2888		85	1a	Bird	bird		Europe	Italy		[illegible]	[illegible]	[illegible]
41	2800/1988	Yptb		1a	Bird	Jack daw	1988	Europe	Finland		[illegible]	[illegible]	[illegible]
41	CK8011	Yptb	54	4a	Mammal	racoon dog	1990	Asia	Japan		[illegible]	[illegible]	[illegible]
41	806	Yptb	8	6	Mammal	rat		Asia	China		[illegible]	[illegible]	[illegible]
41	IP31833	Yptb	48		Mammal	Sheep		Europe	England		[illegible]	[illegible]	[illegible]
42	YT08	Yptb	42		Mammal	Human		North America	Usa		[illegible]	[illegible]	[illegible]
42	YT10	Yptb	42		Mammal	patagonian cavy		North America	Usa		[illegible]	[illegible]	[illegible]
42	YT20	Yptb	42		Mammal	Ground squirrel		North America	Usa		[illegible]	[illegible]	[illegible]
42	YT21	Yptb	42		Mammal	Monkey		North America	Usa		[illegible]	[illegible]	[illegible]
42	YT26	Yptb	42	1b	Mammal	Human		Europe	France		[illegible]	[illegible]	[illegible]
42	YT31	Yptb	42	1a	Mammal	mouse					[illegible]	[illegible]	[illegible]
42	IP32953	Yptb		1b	Mammal	Human		Europe	France		[illegible]	[illegible]	[illegible]
43	YT11	Yptb	14		Mammal	Monkey		North America	Usa		[illegible]	[illegible]	[illegible]
43	YT13	Yptb	14		Bird	Toucan		North America	Usa		[illegible]	[illegible]	[illegible]
43	Y80	Yptb	14								405.404.	[illegible]	[illegible]
43	Ypa727	Yptb									[illegible]	[illegible]	[illegible]
43	No.388	Yptb	24	2b	Mammal	human	1981	Asia	Japan		[illegible]	[illegible]	[illegible]
43	378	Yptb	56	2c	Mammal	hare	1983	Asia	Japan		[illegible]	[illegible]	[illegible]
43	4008/2002	Yptb		15	Mammal	Hare	2002	Europe	Finland		[illegible]	[illegible]	[illegible]
	Y028	Ywau	96		Mammal	badger		Europe	Germany		[illegible]	[illegible]	[illegible]
43	10-218/91	Yptb	21	15	Mammal	human	1993	Asia	Korea		[illegible]	[illegible]	[illegible]
43	Ypa148.88	Yptb									[illegible]	[illegible]	[illegible]
43	Rieter	Yptb	62	4a	Mammal	monkey	1988	Asia	Japan		[illegible]	[illegible]	[illegible]
	CK3086	Yptb	62	3	mammal	racoon dog	1990	Asia	Japan	Okayama	[illegible]	[illegible]	[illegible]
43	No.141	Yptb	39	7	Mammal	mouse	1974	Asia	Japan		[illegible]	[illegible]	[illegible]
43	R2081-2	Yptb	75	7	Mammal	mouse	1982	Asia	Japan		[illegible]	[illegible]	[illegible]
43	R2088-1	Yptb	79	9	Mammal	mouse	1980	Asia	Japan		[illegible]	[illegible]	[illegible]
43	R2031	Yptb	71	11	Mammal	mouse	1980	Asia	Japan		[illegible]	[illegible]	[illegible]
43	CK3108	Yptb		1b	Environm	River water		Asia	Japan	Okayama	[illegible]	[illegible]	[illegible]
43	MW332-1	Yptb	81	10	Environm	water	1991	Asia	Japan		[illegible]	[illegible]	[illegible]
43	WP-931205	Yptb	20	11	Environm	water	1993	Asia	Korea		[illegible]	[illegible]	[illegible]
